# Supplementary material for: Improving best practice for patients receiving hospital discharge letters: a realist review
Source: BMJ Open. 2019 Jun 9;9(6):e027588. doi: 10.1136/bmjopen-2018-027588 (PMC6561435; doi:10.1136/bmjopen-2018-027588)
Supplement: Supplementary data [file bmjopen-2018-027588supp001.pdf]

Search Terms and Sources Searched

Source	Search terms
MEDLINE	1. written[All Fields] AND ("patient discharge"[MeSH Terms]
	2. ("patient"[All Fields] AND "discharge"[All Fields])
	3. ("patient discharge"[All Fields] OR "discharge"[All Fields]) AND ("communication"[MeSH Terms])
	4. ("communication"[All Fields]) AND ("patient discharge"[MeSH Major Topic]) AND ("patients"[MeSH Terms])
	5. ("patients"[All Fields] OR "patient"[All Fields]) OR ("letter"[Publication Type] OR "correspondence as topic"[MeSH Terms])
	6. ("correspondence"[All Fields]) AND ("patients"[MeSH Terms])
	7. ("patients"[All Fields] OR "patient"[All Fields]) AND "patient discharge"[MeSH Major Topic]) AND ("communication"[MeSH Terms])
	8. ("communication"[All Fields]) OR (receiving[All Fields]) AND ("letter"[Publication Type]) OR ("correspondence as topic"[MeSH Terms])
	9. ("letters"[All Fields]) AND ("patients"[MeSH Terms] OR "patients"[All Fields] OR "patient"[All Fields]) AND ("patient discharge"[MeSH Major Topic])
	10. ("patients"[MeSH Terms] OR "patients"[All Fields] OR "patient"[All Fields]) AND ((copies[All Fields]) AND "patient discharge"[MeSH Major Topic]))
	11. ((("patient discharge"[MeSH Major Topic] OR "patient discharge"[MeSH Terms]) AND letter[Other Term]) AND ("patients"[MeSH Terms] OR "patients"[All Fields] OR "patient"[All Fields])
	12. 1 OR 2 OR 3 OR 4 OR 5 OR 6 OR 7 OR 8 OR 9 OR 10 OR 11
Web of Science	1. Written patient discharge communication
	2. Patients receiving letters
	3. Patients receiving discharge letters
	4. Patient copies of written information
	5. 1 OR 2 OR 3 OR 4
Department of Health	1. Discharge communication
	2. Patient letters

	3. Patients receiving letters
Royal College of Physicians	1. Discharge communication<br>2. Patient letters<br>3. Patients receiving letters<br>4. Patient copy<br>5. Patient copies<br>6. Patients receiving written information
