# Supplementary material for: Improving best practice for patients receiving hospital discharge letters: a realist review
Source: BMJ Open. 2019 Jun 9;9(6):e027588. doi: 10.1136/bmjopen-2018-027588 (PMC6561435; doi:10.1136/bmjopen-2018-027588)
Supplement: Supplementary data [file bmjopen-2018-027588supp002.pdf]

Studies Found from Scoping Search

	Author	Year	Title	Document type
1	D N Wood, A Deshpande, M Wijewardena, and S S Gujral	2006	A Study of How Urology Out-Patients like to Receive Clinical Information	Published article
2	A Liapi, P J Robb, and A Akthar	2006	Copying clinic letters to patients: a survey of patient attitudes	Published article
3	S Baxter, K Farrell, C Brown, J Clarke, and H Davies	2008	Where have all the copy letters gone? A review of current practice in professional–patient correspondence	Published article
4	D D Pothier, P Nakivell, and C E J Hall	2007	What do Patients Think about being Copied into their GP Letters?	Published article
5	S L Todhunter, P J Clamp, S Gillett, and D D Pothier	2010	Readability of out-patient letters copied to patients: can patients understand what is written about them?	Published article
6	Royal College of Physicians	2013	Standards for the clinical structure and content of patient records	Guidelines
7	Royal College of Physicians	2017	Writing letters to patients – what's the big deal?	Short website entry
8	A J Choudhry, Y M K Baghdadi, A E Wagie, E B Habermann, S F Heller, D H Jenkins, D C Cullinane, M D Zielinski	2016	Readability of discharge summaries: with what level of information are we dismissing our patients?	Published article
9	M O'Reilly, M R Cahill, and I J Perry	2006	Writing to patients: a randomised controlled trial	Published article
10	Y Krishna, and B E Damato	2005	Patient attitudes to receiving copies of outpatient clinic letters from the ocular oncologist to the referring ophthalmologist	Published article

			and GP	
11	B R O'Driscoll, J Koch, and C Paschalides	2003	Most patients want copies of letters from outpatient clinics and find them useful	BMJ letter
12	H Hadjistavropoulos, H Biem, D Sharpe, M Bourgault-Fagnou, and J Janzen	2008	Patient perceptions of hospital discharge: reliability and validity of a Patient Continuity of Care Questionnaire	Published article
13	M Thornber	2009	Copying referral letters	BJGP letter
14	Department of Health	2000	The NHS Plan	Report
15	P White, A Singleton, and R Jones	2004	Copying referral letters to patients: the views of patients, patient representatives and doctors	Published article
16	NHS England	2016	Standards for the communication of patient diagnostic test results on discharge from hospital	Guidance
17	R Lin, R Gallagher, M Spinaze, H Najoumian, C Dennis, R Clifton-Bligh, and G Tofler	2014	Effect of a patient-directed discharge letter on patient understanding of their hospitalisation	Published article
18	S Vaidyanathan, C A Glass, B M Soni, J Bingley, G Singh, J W H Watt, and P Sett	2001	Doctor ± Patient Communication: Do people with spinal cord injury wish to receive written information about their medical condition from the physicians after an outpatient visit or after a readmission in the spinal	Published article

			unit?	
19	J Flacker, W Park, and A Sims,	2007	Hospital Discharge Information and Older Patients: Do They Get What They Need?	Published article
20	J S Albrecht, A L Gruber-Baldini, J M Hirshon, C H Brown, R Goldberg, J H Rosenberg, A C Comer, and J P Furuno,	2014	Hospital Discharge Instructions: Comprehension and Compliance Among Older Adults	Published article
21	B M Buurman, K J Verhaegh, M Smeulers, H Vermeulen, S E Geerlings, S Smorenburg, and S E de Rooij	2016	Improving handoff communication from hospital to home: the development, implementation and evaluation of a personalized patient discharge letter	Published article
22	Department of Health	2003	Copying letters to Patients: Good practice guidelines	Guidelines
23	J Main	2008	Copying in or copping out?	BMJ letter
24	C D Shee	2008	Try it and see	BMJ letter
25	B McKinstry	2008	Copying patients in is not as simple as it seems	BMJ letter
26	D Jelley, and T van Zwanenberg	2000	Copying general practitioner referral letters to patients: a study of patients' views	Published article
27	K Treacy, J S Elborn, J Rendall, and J M Bradley	2008	Copying letters to patients with cystic fibrosis (CF): Letter content and patient perceptions of benefit	Published article
