# Supplementary material for: Improving best practice for patients receiving hospital discharge letters: a realist review
Source: BMJ Open. 2019 Jun 9;9(6):e027588. doi: 10.1136/bmjopen-2018-027588 (PMC6561435; doi:10.1136/bmjopen-2018-027588)
Supplement: Supplementary data [file bmjopen-2018-027588supp003.pdf]

Sources searched (step 2)

	Sources	Results
1	MEDLINE	1596
2	EMBASE	558
3	CINAHL	100
4	DARE	2
5	ASSIA	47
6	Web of Science	205
7	ZETOC	29
8	AMED	26
9	NHS Digital (HSCIC)	0
10	NHS Evidence (public domain only)	244
11	DH	2
12	NICE Guidelines	0
13	Cochrane database of systematic reviews	21
14	EPPI-CENTRE	20
15	SCOPUS	38
16	Google Scholar	6
17	OpenGrey	3
18	Greynet sources	0
19	ProQuest dissertations and theses	210
20	General Medical Council	0
21	Royal College of Physicians	5
22	Local Medical Committees (West Midlands)	0
23	Clinical Commissioning Groups (West Midlands)	0
24	SIGN	0
25	NHS Improvement	1
	TOTAL RESULTS	3113

Full List of Search Strategies for each source

Search strategy for electronic databases (MEDLINE and AMED)

1. letter$
2. summaries$
3. Correspondence
4. patient copies$
5. patient letter
6. communication (MESH term if MEDLINE)
7. patient$ receiving
8. written information
9. discharge document$
10. patient-directed letter
11. personalised letter
12. personal letter
13. personalized letter
14. copy letter
15. 1 OR 2 OR 3 OR 4 OR 5 OR 6 OR 7 OR 8 OR 9 OR 10 OR 11 OR 12 OR 13 OR 14
16. Hospital discharge (MESH if MEDLINE) AND 15
17. Patient discharge (MESH if MEDLINE) AND 15
18. 16 OR 17
19. 18 and patients (MESH HEADING if MEDLINE)
20. Patients adj3 receiving adj3 letter*
21. Patients adj3 receiving adj3 discharge adj letter*
22. patient adj3 cop$ of written adj information
23. written adj3 patient adj discharge adj communication
24. secondary to primary adj care adj3 communication
25. hospital adj3 GP adj3 communication
26. writing adj3 to adj3 patients
27. 20 OR 21 OR 22 OR 23 OR 24 OR 25 OR 26
28. 27 OR 19
29. patient discharge letter
30. discharge communication
31. discharge letter
32. discharge summary
33. discharge summaries
34. 29 OR 30 OR 31 OR 32 OR 33 OR 34 OR 10 OR 11 OR 12 OR 13 OR 14
35. 34 OR 28
36. discharge correspondence
37. copy correspondence
38. doctor letter
39. copy letter
40. 36 OR 37 OR 38 OR 39
41. 40 OR 35

Search strategy for EMBASE and DARE (adapted due to high search results in EMBASE and simpler searching filtration system in DARE):

1. patient directed letter
2. personalised letter
3. personal letter
4. personalized letter
5. copy letter
6. Patients adj3 receiving adj3 letter*
7. Patients adj3 receiving adj3 discharge adj letter*
8. patient adj3 cop$ of written adj information
9. written adj3 patient adj discharge adj communication
10. secondary to primary adj care adj3 communication
11. hospital adj3 GP adj3 communication
12. writing adj3 to adj3 patients
13. patient discharge letter
14. discharge communication
15. discharge letter
16. patient discharge letter
17. discharge correspondence
18. copy correspondence
19. doctor letter
20. copy letter
21. 1 OR 2 OR 3 OR 4 OR 5 OR 6 OR 7 OR 8 OR 9 OR 10 OR 11 OR 12 OR 13 OR 14 OR 15 OR 16 OR 17 OR 18 OR 19 OR 20
22. 21 OR discharge summary (DARE ONLY)

Search strategy for CINAHL (adapted due to high search results):

1. patient discharge letter AND patient discharge from hospital (CINAHL MH "Hand Off (Patient Safety)")
2. personalised letter AND patient discharge from hospital (CINAHL MH "Hand Off (Patient Safety)")
3. copy letter AND patient discharge from hospital (CINAHL MH patient discharge summaries)
4. discharge communication AND patient discharge from hospital (CINAHL MH patient discharge summaries)
5. discharge correspondence AND patient discharge from hospital (CINAHL MH patient discharge summaries)
6. copy correspondence AND patient discharge from hospital (CINAHL MH patient discharge summaries)
7. doctor letter AND patient discharge from hospital (CINAHL MH patient discharge summaries)
8. copy letter
9. 1 OR 2 OR 3 OR 4 OR 5 OR 6 OR 7 OR 8

ASSIA and Web of Science and ZETOC and NHS evidence search strategy and NHS improvement and Cochrane database

1. (patient discharge letter) AND (discharge from hospital) AND "patient discharge" (*for NHS EVIDENCE & improvement & SCOPUS- AND written)

2. (personalised letter) AND (discharge from hospital) AND "patient discharge" (*for NHS EVIDENCE & improvement & SCOPUS - AND written)
3. (copy letter) AND (discharge from hospital) AND "patient discharge" (*for NHS EVIDENCE & improvement - AND written)
4. (doctor letter) AND (discharge from hospital) AND "patient discharge" (*for NHS EVIDENCE & improvement - AND written)
5. 1 OR 2 OR 3 OR 4
6. (FOR NHS EVIDENCE & improvement ONLY) copying hospital discharge letters to patients

ProQuest very high results (adapted terms)

1. personalised patient discharge letter AND "patient discharge" AND written AND patient information AND copy AND personalised AND copy letter AND doctor letter AND discharge communication copy OR letter "discharge from hospital"Google Scholar search: (many thousands of results when using above terms)

1. (personalised patient discharge letter) AND (discharge from hospital) AND "patient discharge" AND written AND patient information AND copy AND personalised AND copy letter AND doctor letter AND discharge communication AND written AND patient copy

Broad searches for X, Y, Z (websites and sources without indexing or electronic searching) e.g. Department of health

Used for: HSCIC and EPPI-CENTRE And Open Grey

The below search terms were entered into GMC yielding several hundred results. As results from GMC must be exported singularly these were screened on the webpage. The searches found no relevant results.

1. Discharge communication
2. Discharge
3. Patient discharge
4. Discharge letter
5. Discharge letters
6. Discharge summary
7. Discharge summaries
8. Patient letters
9. Patients receiving letters
10. Patients receiving letter
11. Patient copy
12. Copying patients
13. Patient copies
14. Patients receiving written information
15. Hospital discharge
16. Discharge correspondence
17. Discharge document
18. Discharge information
19. Patient discharge information

20. Copy letter
21. Doctor letter
22. Personal letter
23. Copy correspondence
24. Patient involvement
25. Patient access to records
26. Health informatics

*searches adapted in NHS digital due to huge amount of irrelevant results around admission statistics. No relevant results could be found.
