# Supplementary material for: Improving best practice for patients receiving hospital discharge letters: a realist review
Source: BMJ Open. 2019 Jun 9;9(6):e027588. doi: 10.1136/bmjopen-2018-027588 (PMC6561435; doi:10.1136/bmjopen-2018-027588)
Supplement: Supplementary data [file bmjopen-2018-027588supp004.pdf]

List of Included Texts (full)

1. Ackermann S, Bingisser MB, Heierle A, Langewitz W, Hertwig R, Bingisser R. Discharge communication in the emergency department: physicians underestimate the time needed. Swiss Med Wkly. 2012;142:w13588.
2. Adams DC, Bristol JB, Poskitt KR. Surgical discharge summaries: improving the record. Ann R Coll Surg Engl. 1993;75(2):96-9.
3. Advancing effective communication, cultural competence, and patient- and family-centered care : a roadmap for hospitals. Joint Commission. 2014 http://www.jointcommission.org/assets/1/6/aroadmapforhospitalsfinalversion727.pdf.
4. Aguayo-Albasini JL, Garcia Garcia ML, Flores-Pastor B, Liron-Ruiz R. The importance of the discharge summary reports. Cir Esp. 2014;92(8):574-5.
5. Allan K, Ribbons B. Nurses combine IT and nursing skills to improve discharge communication. Aust Nurs J. 2006;14(1):30.
6. Antoniou A, Saunders M, Bourner R, Crouch L. would you like to see yours? The Bulletin of the Royal College of Surgeons of England. 2007;89(2):62-4.
7. Baumann W, Schussler L, Bertram M, Benser J, Kumpers S, Hermes-Moll K. Oncologists' letters for breast cancer patients. Oncology Research and Treatment. 2016;39:184-5.
8. Baxter S, Farrell K, Brown C, Clarke J, Davies H. Where have all the copy letters gone? A review of current practice in professional-patient correspondence. Patient Educ Couns. 2008;71(2):259-64.
9. Bench S, Day T, Griffiths P. Effectiveness of critical care discharge information in supporting early recovery from critical illness. Crit Care Nurse. 2013;33(3):41-52.
10. Bench SD, Heelas K, White C, Griffiths P. Providing critical care patients with a personalised discharge summary: a questionnaire survey and retrospective analysis exploring feasibility and effectiveness. Intensive Crit Care Nurs. 2014;30(2):69-76.
11. Bench S, Day T, Heelas K, Hopkins P, White C, Griffiths P. Evaluating the feasibility and effectiveness of a critical care discharge information pack for patients and their families: a pilot cluster randomised controlled trial. BMJ Open. 2015;5(11):e006852.
12. Boaden R, Harris C. Copying letters to patients—will it happen? : Oxford University Press; 2005.
13. Brockbank K. Copying patient letters - Making it work. Clinical Governance. 2005;10(3):231-40.
14. Brodie T, Lewis D. A survey of patient views on receiving vascular outpatient letters. European Journal of Vascular and Endovascular Surgery. 2010;39(1):5-10.
15. Brown CE, Roberts NJ, Partridge MR. Does the use of a glossary aid patient understanding of the letters sent to their general practitioner? Clinical medicine (London, England). 2007;7(5):457-60.
16. Buurman BM, Verhaegh KJ, Smeulers M, Vermeulen H, Geerlings SE, Smorenburg S, et al. Improving handoff communication from hospital to home: the development, implementation and evaluation of a personalized patient discharge letter. International Journal for Quality in Health Care. 2016;28(3):384-90.
17. Cannaby A-M. Improving the process of hospital discharge for medical patients [Ph.D.]. Ann Arbor: University of Leicester (United Kingdom); 2003.
18. Carol Lim KK, Chan SK, Chew EL, Anita Lim AF, Sararaks S, Ainul H, et al. Handoff communication - Let's do it right. Medical Journal of Malaysia. 2010;65:8.
19. Chantler C, Johnson J. Patients should receive copies of letters and summaries. BMJ : British Medical Journal. 2002;325(7360):388-.

20. Charlett SD, Bajaj Y, Kelly G. Writing to patients with the results of routine tests: A measure to improve access to outpatient clinics. Otorhinolaryngologist. 2009;2(3):73-4.
21. Choudhry AJ, Baghdadi YM, Wagie AE, Habermann EB, Heller SF, Jenkins DH, et al. Readability of discharge summaries: with what level of information are we dismissing our patients? Am J Surg. 2016;211(3):631-6
22. Damian D, Tattersall MH. Letters to patients: improving communication in cancer care. Lancet. 1991;338(8772):923-5.
23. Davies JM, Batuyong E, Lupichuk SM, Hilsden R, Eliasziw M, Easaw JC. Cohort study evaluating the impact of a discharge letter (DL) compared with usual care on adherence to surveillance following treatment for stage II/III colorectal cancer (CRC). Journal of Clinical Oncology Conference. 2012;30(4 SUPPL. 1).
24. Department of Health. The NHS Plan. 2000 http://webarchive.nationalarchives.gov.uk.
25. Department of Health. Copying letters to patients: good practice guidelines. 2003 http://webarchive.nationalarchives.gov.uk/
26. Discharge planning : best practice in transitions of care. The Queen's Nursing Institute. 2016 https://www.qni.org.uk/wpcontent/uploads/2016/09/discharge_planning_report_2015.pdf.
27. Dooher P, Syed A, Liu J, Chopra A, Bradpiece H, Jenkins S, et al. Copying letter to patients-distress or satisfaction? Eur J Cancer. 2012;48:S151.
28. Exploring patient participation in reducing health-care-related safety risks. 2013 http://www.euro.who.int/__data/assets/pdf_file/0010/185779/e96814.pdf.
29. Fayers T, Abdullah W, Walton V, Wilkins MR. Impact of written and photographic instruction sheets on patient behavior after cataract surgery. J Cataract Refract Surg. 2009;35(10):1739-43.
30. Fenton C, Al-Ani A, Trinh A, Srinivasan A, Marion K, Hebbard G. Impact of providing patients with copies of their medical correspondence: a randomised controlled study. Intern Med J. 2017;47(1):68-75.
31. Generic Standards Mar 2002. 2002 http://www.healthcareimprovementscotland.org/his/idoc.ashx?docid=ea8d290a-9504-4c21-a889-fc479d530a51&version=-1.
32. Guidelines on regional immediate discharge documentation for patients being discharged from secondary into primary care. Guidelines and Audit Implementation Network (GAIN). 2011 https://www.rqia.org.uk/RQIA/files/73/734a792f-f9d4-47f0-830f-31f9db51c82a.pdf**.**
33. Hahn-Goldberg S, Okrainec K, Damba C, Huynh T, Lau D, Maxwell J, et al. Implementing Patient-Oriented Discharge Summaries (PODS): A Multisite Pilot Across Early Adopter Hospitals. Healthc Q. 2016;19(1):42-8.
34. Hallowell N. Providing letters to patients. Patients find summary letters useful. Bmj. 1998;316(7147):1830.
35. Hayes KS. Literacy for health information of adult patients and caregivers in a rural emergency department. Clin Excell Nurse Pract. 2000;4(1):35-40.
36. HPOE: A Compendium of Implementation Guides 2011. 2011 http://www.hpoe.org/Reports-HPOE/hpoe_compendium_2011.pdf.
37. Hoek AE, De Ridder MA, Bayliss A, Patka P, Rood PP. Effective strategy for improving instructions for analgesic use in the emergency department. Eur J Emerg Med. 2013;20(3):210-3.
38. Holm H, Viktil KK. Patient satisfaction with drug reconciliation at discharge from hospital. Int J Clin Pharm. 2013;35 (6):1284.

39. Jelley D, van Zwanenberg T, Walker C. Copying letters to patients : Concerns of clinicians and patients need to be addressed first. BMJ : British Medical Journal. 2002;325(7376):1359-.
40. Knight AH, Mayon-White V. Writing to patients--annual review reports. Diabet Med. 1991;8(6):591.
41. Krishna Y, Damato BE. Patient attitudes to receiving copies of outpatient clinic letters from the ocular oncologist to the referring ophthalmologist and GP. Eye (London, England). 2005;19(11):1200-4.
42. Lepping P, Paravastu SC, Turner J, Billings P, Minchom P. Copying GP letters to patients: a comprehensive study across four different departments in a district general hospital. Health Inform J. 2010;16(1):58-62.
43. Liapi A, Robb PJ, Akthar A. Copying clinic letters to patients: a survey of patient attitudes. The Journal of Laryngology & Otology. 2006;121(6):588-91.
44. Lim CKK, Lim AAF, Ainul Nadziha MH, Roslinah A, Sararaks S, Chan SK, et al. Boleh balik! Medical Journal of Malaysia. 2010;65:69.
45. Lin R, Tofler G, Spinaze M, Dennis C, Clifton-Bligh R, Nojoumian H, et al. Patient-directed discharge letter (PADDLE)-a simple and brief intervention to improve patient knowledge and understanding at time of hospital discharge. Heart Lung and Circulation. 2012;21:S312.
46. Lin R, Gallagher R, Spinaze M, Najoumian H, Dennis C, Clifton-Bligh R, et al. Effect of a patient-directed discharge letter on patient understanding of their hospitalisation. Internal Medicine Journal. 2014;44(9):851-7.
47. Lin MJ, Tirosh AG, Landry A. Examining patient comprehension of emergency department discharge instructions: Who says they understand when they do not? Intern. 2015;10(8):993-1002.
48. Lorenzati B, Quaranta C, Perotto M, Tartaglino B, Lauria G. Discharge communication is an important underestimated problem in emergency department. Intern. 2016;11(1):157-8
49. Main J. Copying in or copping out? Bmj. 2008;337:a2688.
50. Making time in general practice : freeing GP capacity by reducing bureaucracy and avoidable consultations, managing the interface with hospitals and exploring new ways of working. NHS Alliance. 2015 http://www.nhsalliance.org/wp-content/uploads/2015/10/Making-Time-in-General-Practice-FULL-REPORT-01-10-15.pdf.
51. McConnell D, Butow P, Tattersall M. Audiotapes and letters to patients: the practice and views of oncologists, surgeons and general practitioners. British Journal of Cancer. 1999;79(11-12):1782.
52. McEniry B, Pillay I. How do patients in a rural setting respond to receiving a copy of their general practitioners letter? Ir Med J. 2008;101(3):84-5.
53. McKinstry B. Copying patients in is not as simple as it seems. Bmj. 2008;337:a2687.
54. Menon GJ, Dutton GN. Writing to our patients. Br J Ophthalmol. 1999;83(7):765.
55. Mrduljas Dujic N, Zitnik E, Pavelin L, Bacic D, Boljat M, Vrdoljak D, et al. Writing letters to patients as an educational tool for medical students. BMC Med Educ. 2013;13:114.
56. Mrduljas-Djujic N, Pavlicevic I, Marusic A, Marusic M. Students letters to patients as a part of education in family medicine. Acta Med. 2012;41(1):52-8.
57. NHS England . Standards for the communication of patient diagnostic test results on discharge from hospital. 2016. https://improvement.nhs.uk/uploads/documents/discharge-standards-march-16.pdf.
58. Nixon J, Courtney P. Copying clinic letters to patients. Rheumatology (Oxford). 2005;44(2):255-6.

59. O'Driscoll BR, Koch J, Paschalides C. Copying letters to patients: Most patients want copies of letters from outpatient clinics and find them useful. BMJ : British Medical Journal. 2003;327(7412):451-.
60. O'Reilly M, Cahill MR, Perry IJ. Writing to patients: a randomised controlled trial. Clin Med. 2006;6(2):178-82.
61. Paravastu S, Lepping P, Billings P. Copying clinic letters to surgical patients. The Bulletin of the Royal College of Surgeons of England. 2007;89(8):288-90.
62. Partridge MR, Roberts NJ. Writing to patients. Clin Med. 2006;6(3):319.
63. Perera KY, Ranasinghe P, Adikari AM, Balagobi B, Constantine GR, Jayasinghe S. Medium of language in discharge summaries: would the use of native language improve patients' knowledge of their illness and medications? J Health Commun. 2012;17(2):141-8
64. Perkins P, Jordan A, Prentice W, Regnard C. Copying letters to patients: a survey of patients and GPs views. Palliat Med. 2007;21(4):355-6.
65. Physicians RCo. Standards for the clinical structure and content of patient records. 2013 https://www.rcplondon.ac.uk/projects/outputs/standards-clinical-structure-and-content-patient-records.
66. Physicians RCo. Writing letters to patients – what's the big deal? 2017 https://www.rcplondon.ac.uk/news/writing-letters-patients-what-s-big-deal.
67. Pierce L. How to choose and develop written educational materials. Rehabilitation Nursing. 2010;35(3):99-105.
68. Pinder E, Jefferys S, Loeffler M. Patient Satisfaction: Receiving a copy of the GP letter following fracture or elective orthopaedic clinic. BMJ Quality Improvement Reports. 2013;2(2).
69. Polster D. Patient discharge information. Nursing. 2015;45(5):42-9.
70. Pothier DD, Nakivell P, Hall CE. What do patients think about being copied into their GP letters? Ann R Coll Surg Engl. 2007;89(7):718-21.
71. PRSB. Outpatient letter standard Consultation survey. 2017.
72. Rao M, Fogarty P. What did the doctor say? J Obstet Gynaecol. 2007;27(5):479-80.
73. Reddick B, Holland C. Reinforcing discharge education and planning. Nurs Manage. 2015;46(5):10-4.
74. Regalbuto R, Maurer MS, Chapel D, Mendez J, Shaffer JA. Joint Commission requirements for discharge instructions in patients with heart failure: is understanding important for preventing readmissions? J Card Fail. 2014;20(9):641-9.
75. Reilly MM. Let's set the record straight: preparing the discharge summary and the patient's instruction sheet. Nursing. 1979;9(1):56-61.
76. Reilly MO, Cahill M, Perry IJ. Writing to patients: 'putting the patient in the picture'. Ir Med J. 2005;98(2):58-60.
77. Roberts NJ, Partridge MR. How useful are post consultation letters to patients? BMC Medicine. 2006;4:2-.
78. Saidinejad M, Zorc J. Mobile and web-based education: delivering emergency department discharge and aftercare instructions. Pediatr Emerg Care. 2014;30(3):211-6.
79. Samuels-Kalow M, Rhodes K, Uspal J, Reyes Smith A, Hardy E, Mollen C. Unmet Needs at the Time of Emergency Department Discharge. Acad Emerg Med. 2016;23(3):279-87
80. Sandler DA, Heaton C, Garner ST, Mitchell JR. Patients' and general practitioners' satisfaction with information given on discharge from hospital: audit of a new information card. Bmj. 1989;299(6714):1511-3.
81. Sandler DA, Mitchell JR, Fellows A, Garner ST. Is an information booklet for patients leaving hospital helpful and useful? Bmj. 1989;298(6677):870-4.

82. Saunders NC, Georgalas C, Blaney SP, Dixon H, Topham JH. Does receiving a copy of correspondence improve patients' satisfaction with their out-patient consultation? J Laryngol Otol. 2003;117(2):126-9.
83. Sharma D, O'Brien S, Hardy K. Copying letters to patients: What patients think - A questionnaire survey. Clinician in Management. 2007;15(2):75-8.
84. Shee CD. Try it and see. Bmj. 2008;337:a2786.
85. Singh S, Budeda B, Housden P. Do patients want copies of their GP letters?–our experience with 7250 patients. Int J Clin Pract. 2007;61(8):1407-9.
86. Smith PEM. Letters to patients: sending the right message. BMJ : British Medical Journal. 2002;324(7338):685-.
87. Somov P, Madden T, Wong K, Hamm R. Security Concerns About Copying Clinical Letters to Patients. The Bulletin of the Royal College of Surgeons of England. 2013;95(1):33-4.
88. Sparkler 1 - Transitions of care in elderly patients. 2014 http://emahsn.org.uk/images/Section%208%20-%20Resource%20hub/Sparks%20and%20Sparklers/Sparkler_1_v6_SP1V1_FINAL_pdf_01-08-14.pdf.
89. Synthesis and conceptual analysis of the SDO programme's research on continuity of care. National Institute for Health Research (NIHR). 2010 http://www.netscc.ac.uk/hsdr/files/project/SDO_FR_08-1813-248_V01.pdf.
90. Tattersall R. Writing for and to patients. Diabet Med. 1990;7(10):917-9.
91. Taylor DM, Cameron PA. Discharge instructions for emergency department patients: What should we provide? Journal of Accident and Emergency Medicine. 2000;17(2):86-90.
92. Thornber M. Copy them in. Bmj. 2008;337.
93. Todhunter SL, Clamp PJ, Gillett S, Pothier DD. Readability of out-patient letters copied to patients: can patients understand what is written about them? J Laryngol Otol. 2010;124(3):324-7.
94. Tomkins CS, Braid JJ, Williams HC. Do dermatology outpatients value a copy of the letter sent to their general practitioner? In what way and at what cost? Clin Exp Dermatol. 2004;29(1):81-6.
95. Treacy K, Elborn JS, Rendall J, Bradley JM. Copying letters to patients with cystic fibrosis (CF): letter content and patient perceptions of benefit. Journal of cystic fibrosis : official journal of the European Cystic Fibrosis Society. 2008;7(6):511-4.
96. The Newcastle upon Tyne Hospitals NHS Foundation Trust Sharing Letters with Patients Policy. 2013.
97. Vaidya G. Copying letters to patients: Are we ready yet? Hospital Medicine. 2004;65(8):454-5.
98. Verhaegh KJ, Buurman BM, Veenboer GC, de Rooij SE, Geerlings SE. The implementation of a comprehensive discharge bundle to improve the discharge process: a quasi-experimental study. Neth J Med. 2014;72(6):318-25.
99. Walji M, Loeffelholz J, Valenza JA. A human-centered design of a dental discharge summary (DDS) for patients. AMIA Annu Symp Proc. 2007;Annual Symposium Proceedings/AMIA Symposium.:1146.
100. Warren J, Adnan M, Orr M. Iterative refinement of SemLink to enhance patient readability of discharge summaries. Stud Health Technol Inform. 2013;188:128-34.
101. Wimsett J, Harper A, Jones P. Review article: Components of a good quality discharge summary: a systematic review. Emerg Med Australas. 2014;26(5):430-8.

102. Zavala S, Shaffer C. Do patients understand discharge instructions? J Emerg Nurs. 2011;37(2):138-40.
103. Zeng-Treitler Q, Kim H, Hunter M. Improving patient comprehension and recall of discharge instructions by supplementing free texts with pictographs. AMIA Annu Symp Proc. 2008;Annual Symposium Proceedings/AMIA Symposium.:849-53.
