# Supplementary material for: Improving best practice for patients receiving hospital discharge letters: a realist review
Source: BMJ Open. 2019 Jun 9;9(6):e027588. doi: 10.1136/bmjopen-2018-027588 (PMC6561435; doi:10.1136/bmjopen-2018-027588)
Supplement: Supplementary data [file bmjopen-2018-027588supp005.pdf]

CMO Table

	Context	Mechanism	Outcome	Effect assessment	Does it "work" or not?
CMOC1	patient not offered letter	patient feels less involved in care	reduced patient autonomy	negative	does not work
CMOC2	patient offered opportunity to receive letter(s)/patient choice respected	patient feels more informed and involved in care	increased patient autonomy and increased involvement of patients in treatment, care and communications	positive	does work
CMOC3	large clear posters displaying patients right to choose and importance of correct contact information	patient realises they should inform hospital of address changes and preferences	lowered risk of confidentiality breach	positive	does work
CMOC4	NHS drive for patient-led care (influence or context)	clinicians increasingly offering patient choice of receiving letter/sharing information with patients	increased patient empowerment	positive	does work
CMOC5	clinician views letters to patients are beneficial e.g. increases transparency, compliance, trust, patient satisfaction, patient understanding and recall	clinician feels patient should be offered letter	potential increase in patient autonomy & satisfaction	positive	does work
CMOC6	Clinicians views letters to patients as not beneficial e.g. letter not comprehensible to patient, medico-legal issues, increased cost and staff workload, patient harm (anxiety, distress, and confusion) and issues around confidentiality	clinician feels patient should not be offered letter	no patient autonomy	N/A	unclear
CMOC7	NHS guidance that all hospital-GP correspondence should be copied to patient as a "right" where appropriate and if patients agree (unless risk of serious harm or legal issues)	clinicians increasingly offering patient choice of receiving letter	increased use of NHS resources to send letters but patient benefits through increased understanding & potential reduction in patient queries (costs balanced)	positive	does work
CMOC8	Data Protection Act 1998 (UK)	Patients may become aware of their right to know what is written & stored about them	Patients informed of their stored electronic information (increased transparency)	positive	does work
CMOC9	doctors copy patients letters	patient trusts doctor more	improved doctor-patient relationship	positive	does work
CMOC10	patients offered choice of receiving letters	increased no. of patients choosing to receive letters	Increased administrative staff workload and costs of printing & posting letters	negative	unclear

	Context	Mechanism	Outcome	Effect assessment	Does it "work" or not?
CMOC11	patients offered choice of receiving letters	increased no. of patients choosing to receive letters	reduced queries and GP visits and reduced hospital re-admissions (limited evidence)	positive	does work
CMOC12	structured discharge letters written clearly in plain English (pref. 5th grade level) with medical jargon defined, no value judgements of patients and minimal abbreviations	patients understand letter	increased patient knowledge	positive	does work
CMOC13	doctors provided training in letter writing & record keeping (contextual influence) leading to doctors write letters of higher quality and more appropriate for patients	patients understand letter	Increased patient knowledge/potential increase in doctor confidence in letter writing	positive	does work
CMOC14	patients preference for letter copies acknowledged and patients offered choice of receiving letter	patients feel able to express their preference	decreased strain on resources & increased patient autonomy & satisfaction	positive	does work
CMOC15	patients provided written & verbal information	patients reflect on written record of information for reference	increased patient knowledge of care plan, recall and acceptance of illness or condition	positive	does work
CMOC16	Human Rights Act (1998) and Race Revelations Act (2000) - clinicians equally offer all patients letter copies regardless of background	clinician feels all patients should be offered letter	increased equality and accessibility of information to patients	positive	does work
CMOC17	Use of pictures/pictographs/cartoons with written information	patients understand letter	Patient benefits from improved understanding e.g. adherence to agreed care plan	positive	does work
CMOC18	verbal information only	patient may not be able to retain information	reduced patient recall	negative	does not work
CMOC19	professionals who are not involved/limited involvement with patient writes letter	professional does not understand patient plan	letter quality reduced/increased risk of harm	negative	does not work
CMOC20	patient hospital visit of sensitive nature and/or patient lacks capacity e.g. psychotic episode, dementia	patient finds letter distressing and/or confusing	harm to patient	negative	does not work
CMOC21	Patient letter written above patient educational level or in a language the patient does not read	patient finds letter difficult to understand	patient is confused with no increased knowledge of care/possible misinterpretation of care instructions	negative	does not work
CMOC22	letter contains inaccurate information	patient identifies inaccuracies	patient notifies hospital/GP of inaccuracies and corrections are made leading to improved record keeping	positive	does work

	Context	Mechanism	Outcome	Effect assessment	Does it "work" or not?
CMOC23	patients receives discharge letter	patient does not understand entirety of letter	patient sources answers (internet, GP, friend or relative)	positive	does work
CMOC24	Patient specific letter sent to patient	patient finds letter clear	improved patient comprehension/patient may use letter as aid to explain condition to family and friends	positive	does work
CMOC25	Patient specific letter sent to patient		increased staff workload and costs	negative	does not work
CMOC26	Patient specific letter sent to patient	Patient identifies information sent to GP and patient is different	medico-legal concerns could be raised over letter discrepancies and any withheld information	negative	does not work
CMOC27	hospital sends patient discharge letter without verifying patient contact details without notifying patient	hospital worker does not identify and correct incorrect information	potential breach of patient confidentiality	negative	does not work
CMOC28	hospital routinely checks patient addresses and sends discharge letters to patients marked confidential using full name	hospital worker identifies and corrects incorrect information	patient receives letter, minimal risk of patient confidentiality breach	positive	does work
CMOC29	patient receives discharge letter	patient may feel they have questions relating to letter	patient contacts health provider with queries (evidence suggests minimal impact and queries)	positive	unclear
CMOC30	discharge letter/summary dictated in front of patient	patients query any inaccuracies	letter less likely to contain inaccuracies	positive	does work
CMOC31	Hospital gives patient discharge letter/summary to deliver to GP	patient may find they are unable to make delivery	GP does not always receive letter/summary	negative	does not work
CMOC32	Patient receives letter not written at appropriate level for them	patient feels confused and does not understand letter	GP spends time reassuring patient and explaining letter to ease patient upset	negative	does not work
CMOC33	Patients have anxiety that doctors talk about things behind their backs	patients who receive letter feel reassured that there is no hidden information	decreased patient anxiety and improved doctor-patient relationship through transparency	positive	does work
CMOC34	patients receives discharge letter	Patients feel they are important to clinician	patient is impressed with letter and feels clinician has an interest in them	positive	does work
CMOC35	choice about whether letter is sent to patient	clinician feels letters would be a disaster and inappropriate for patients	patients do not receive letters	N/A	unclear
CMOC36	patients receives discharge letter		no impact on patient	N/A	unclear
CMOC37	patients receives discharge letter with bad news	Patient finds letter initially distressing	letter causes initial distress but final outcome that patient finds letter helpful and aids recall and acceptance of condition	positive	does work

	Context	Mechanism	Outcome	Effect assessment	Does it "work" or not?
CMOC38	letter sent to patient containing information not discussed with patient or abnormal results	patient feels distressed and anxious reading letter	patient harm/unethical practice	negative	does not work
CMOC39	patient worried about diagnosis and receives letter	patient understanding helped by letter	patient feels less anxious due to being more informed	positive	does work
CMOC40	patients preference for letter copies not acknowledged		patient may receive letter who didn't want one leading to decreased patient satisfaction	negative	does not work
CMOC41	(best practice) patients offered choice of receiving letters/opt-in system	patients enabled to decide on letter preference	patients may or may not receive letter depending on their preference resulting in higher patient satisfaction	positive	does work
CMOC42	patients who feel copies of letters are not necessary for themselves		patient not given letter so patient satisfied, secondary outcomes: costs and time saved	positive	does work
CMOC43	patients receives discharge letter where appropriate	patient understands letter (high evidence)	patient finds letter informative and helpful	positive	does work
CMOC44	patients receives discharge letter where appropriate	patient feels involved in care plan	patient ensures follow up plan is followed and books any necessary tests etc.	positive	does work
CMOC45	patients receives discharge letter where appropriate	patient feels letter is important	letter forms permanent record of hospital visit and kept for future reference	positive	does work
CMOC46	patients receives discharge letter for breaking good news	patient reminded of discussion	patient feels reassured and has "peace of mind"	positive	does work
CMOC47	patients receives discharge letter where appropriate (patient choice)	patient likes receiving letter	patient satisfaction increased	positive	does work
CMOC48	patients receives copy of discharge letter where appropriate	patient becomes aware of what GP knows	Patient reassured that GP knows about visit	positive	does work
