# Supplementary material for: Improving best practice for patients receiving hospital discharge letters: a realist review
Source: BMJ Open. 2019 Jun 9;9(6):e027588. doi: 10.1136/bmjopen-2018-027588 (PMC6561435; doi:10.1136/bmjopen-2018-027588)
Supplement: Author's manuscript [file bmjopen-2018-027588.draft_revisions.pdf]

BMJ Open is committed to open peer review. As part of this commitment we make the peer review history of every article we publish publicly available.

When an article is published we post the peer reviewers' comments and the authors' responses online. We also post the versions of the paper that were used during peer review. These are the versions that the peer review comments apply to.

The versions of the paper that follow are the versions that were submitted during the peer review process. They are not the versions of record or the final published versions. They should not be cited or distributed as the published version of this manuscript.

BMJ Open is an open access journal and the full, final, typeset and author-corrected version of record of the manuscript is available on our site with no access controls, subscription charges or pay-per-view fees (http://bmjopen.bmj.com).

If you have any questions on BMJ Open's open peer review process please email info.bmjopen@bmj.com

BMJ Open

# Improving best practice for patients receiving hospital discharge letters: a realist review



| | |
|---|---|
| Journal: | *BMJ Open* |
| Manuscript ID | bmjopen-2018-027588 |
| Article Type: | Research |
| Date Submitted by the Author: | 30-Oct-2018 |





| | |
|---|---|
| Complete List of Authors: | Weetman, Katharine; University of Warwick Warwick Medical School<br>Wong, Geoffrey; University of Oxford, Nuffield Department of Primary Care Health Sciences<br>Scott, Emma; University of Warwick Warwick Medical School<br>MacKenzie, Eilidh; University of Warwick Warwick Medical School<br>Schnurr, Stephanie; University of Warwick, Centre for Applied Linguistics<br>Dale, Jeremy; University of Warwick, Warwick Medical School |





| | |
|---|---|
| Keywords: | communication, hospital discharge, patient information, discharge letter, realist review |



SCHOLARONE™
Manuscripts

# Improving best practice for patients receiving hospital discharge letters: a realist review

**Katharine Weetman[1], Geoff Wong[2], Emma Scott[3], Eilidh MacKenzie[4], Stephanie Schnurr[5], Jeremy Dale[6]**

1. Unit of Academic Primary Care, Division of Health Sciences, Warwick Medical School, University of Warwick, Coventry, UK, CV4 7AL, **Doctoral Researcher**
2. Nuffield Department of Primary Care Health Sciences, Medical Sciences Division, University of Oxford, Oxford, UK, OX2 6GG, **Clinical Research Fellow**
3. Unit of Academic Primary Care, Division of Health Sciences, Warwick Medical School, University of Warwick, Coventry, UK, CV4 7AL, **Senior Research Fellow**
4. Warwick Medical School, University of Warwick, Coventry, UK, CV4 7AL, **Medical Student**
5. Centre for Applied Linguistics, University of Warwick, Coventry, UK, CV4 7AL, **Associate Professor**
6. Unit of Academic Primary Care, Division of Health Sciences, Warwick Medical School, University of Warwick, Coventry, UK, CV4 7AL, **Professor**

Correspondence to:
Katharine Weetman
Warwick Medical School, University of Warwick, Coventry, UK, CV4 7AL
K.Weetman@warwick.ac.uk
+44 (0)2476 151077

Word count, excluding title page, abstract, bibliography, figures & tables:

6,691

Word count, excluding title page, headings, abstract, references, figures & tables and quotes:

5,452

**Key words:** Communication; discharge letter; hospital discharge; patient information; realist review

**Study area:** General health



For peer review only - http://bmjopen.bmj.com/site/about/guidelines.xhtml

## TITLE

Improving best practice for patients receiving hospital discharge letters: a realist review


## ABSTRACT

**Objective:** To understand how outcomes are achieved from adult patients receiving copies of hospital discharge letters.

**Design:** A realist review conducted in three phases: (1) literature scoping to develop an initial programme theory (PT) relating to copying patients into written discharge communication from hospital inpatient and outpatient settings; (2) structured searching, retrieval and extraction of evidence; and (3) analysis and synthesis to refine the PT with stakeholder (general practitioners and health service commissioners) and patient input.

**Eligibility criteria:** Studies and publications reporting evidence that met criteria for relevance to the PT. Studies relating solely to mental health or children aged <18yrs were excluded.

**Analysis:** Data was extracted and analysed using a realist logic of analysis. Texts were annotated for concepts relating to context, mechanism and outcome configurations (CMOCs).

**Results:** 3113 documents were screened, and following appraisal and hand-searching 103 source texts were included. Stakeholders and patients contributed to refining the PT. The final PT included 48 CMOCs for how patients receiving discharge letters affects outcomes. Key concepts that affected the likelihood of beneficial outcomes were: patient choice; comprehension, queries and recall; personalised or patient-directed discharge letters; patient to deliver letter; dictating letters in front of patients; confidentiality; patient harm; clinician views; cost and resources; doctor-patient relationships and autonomy. Two key findings were that patient understanding is possibly greater than clinicians perceive, and that patients tend to express strong preference for receiving their letters. Clinician attitudes were identified as a barrier to initiatives for wider sharing of discharge letter with patients, which may need to be addressed through organisational policies and direction.

**Conclusions:** This review forms a starting point for explaining outcomes associated with whether or not patients receive discharge letters. It suggests several ways in which current processes might be modified to support improved practice and patient experience.


**Strengths and limitations of this study:**

- First study to review and develop theories about patients receiving discharge letters.
- The engagement of patients, GPs and policy makers in refining the programme theory increased relevance and rigour of the theory.
- The programme theory is likely to be applicable and relevant to multiple healthcare settings.
- The exclusion criteria imposed restrictions on the programme theory such that evidence relating to children, those with mental health problems or lacking capacity is not considered.
- Only sources written in the English Language were included.



For peer review only - http://bmjopen.bmj.com/site/about/guidelines.xhtml

## INTRODUCTION

### Background

*Discharge communication* may follow an inpatient or outpatient discharge; it typically comprises written discharge information in the form of a discharge letter or summary. It is a well-established practice that the physician who is to follow up patient care, typically the GP or equivalent [(1)], should receive written *discharge communication* from the discharging physician; this practice supports continuity of care between specialist services and primary care. Patients are sometimes included in this communication, and while within the UK this is considered to be ‘good practice’ [(2)], is not standardised.

The *Department of Health* in the UK describes patient copies of letters as a “right” and recommend patients should be copied in where appropriate as a “rule”, unless there is risk of harm [(2, 3)]. This practice was intended to support patient understanding and wellbeing, increase patient safety and the quality of information sent, and improve doctor-patient relationships [(2, 3)]. More recently, the *Academy of Medical Royal Colleges (AMRC)* released the “please write to me” [(4)] initiative. The initiative’s purpose is to encourage doctors to write to directly patients in simple plain English to increase understanding. Despite these initiatives and guidelines, studies within and outside the UK report both benefits [(5-13)] (e.g. high patient satisfaction), and drawbacks [(9, 10, 13-16)] (e.g. patient confusion) of patients receiving their letters. Hence, many patients do not receive copies of such letters, but the reasons for this and the subsequent consequences remain unclear [(2, 3, 17)].

We recently published a protocol [(18)] for this review which fully details the background and methods we used. As summarised in the protocol paper:

> “Whether or not it is beneficial for patients to receive written discharge communication, and, if so, for whom, when, how, why, and whether this should be a direct copy or personalised letter remains equivocal. We could find no review specific to this question; we only found reviews of copying letters in general [(19, 20)].” [(18)]

Thus, the evidence on patients receiving discharge letters is unclear and it was concluded that consolidation of the evidence through a realist review is required.

## METHODS

A realist review is a, ‘theory-driven, interpretative approach to the synthesis of evidence’ [(21)]. Synthesising evidence involves interrogating data sources to develop, refine and test *context, mechanism,* and *outcome* configurations (CMOCs). "Context may be conceptualised as external factors that influence mechanisms [(22)]. “Mechanisms" are hidden, context causal forces that produce "outcomes".[(22)] Following Pawson [(22-25)], CMOCs should be configured and consolidated to build and develop a realist *programme theory* or theorised explanation of how an intervention *works* or not. The intervention under scrutiny ‘patients receiving discharge letters’ was defined by the review team as ‘the patient being given or sent any form of written (paper or digital) hospital discharge communication; this could be a direct copy, patient-directed letter, or a combination.’

The protocol [(18)] considers realist review methodology in depth and argues a realist approach is apt and useful approach for this review. Briefly, we argued that: a realist review has the potential to identify *how* positive outcomes may be reproduced



For peer review only - http://bmjopen.bmj.com/site/about/guidelines.xhtml

and has capacity to account for *complexity.* The intervention is complex in that the *form* of discharge communication can vary and the *quality* of communication is highly context-dependent.

The review design (Figure 1) was informed by previous literature, consists of six steps [22, 26, 27] and is further described in the protocol paper [18].

*Figure 1 Review design*

The aim of the review is to understand and explain how the different outcomes are produced from adult patients receiving written discharge letters. Outcomes may be simplified into desired/beneficial or 'positive' (e.g. satisfaction) and undesired/detrimental or 'negative' (e.g. anxiety). The objectives are to conduct a realist review of the intervention (patients receiving copies of discharge communication); to develop a programme theory (PT); and to make best practice recommendations for the intervention.

The research questions (RQs) are as follows:

RQ1: What positive and negative outcomes have been reported on patients receiving written discharge communication?

RQ2: What are the important contexts which are associated with whether the mechanisms produce the different outcomes, and why?

**Programme theory development (step 1)**

The task of locating existing theories to develop an initial rough PT was achieved through a scoping search. Theories were sought which contributed toward understanding how patients receiving discharge letters *works* or not. Search terms were based on the intervention (e.g. patient cop(y)ies). Published resources and healthcare websites were searched to ascertain a range of evidence (see *Supplementary file 1*). Sources were selected based on their "relevance" [22-24] to the PT; where *relevance* concerns 'does the [source] address the theory under test?' [23]. Crucially, the whole source did not need to inform the PT but we considered the relevance and contribution of sections of the document [7]. During this phase, research team judgement was needed to decide the stopping point for programme theory development as was the need to balance the degree of comprehensiveness and practicalities [18].

Twenty seven documents were selected from the scoping search (see *Supplementary file 2*). All documents were then interrogated and coded for any CMOCs, concepts, or theories which could inform development of a PT. These were consolidated to form Figure 2, the initial PT.



For peer review only - http://bmjopen.bmj.com/site/about/guidelines.xhtml

*Figure 2 Initial programme theory*

For peer review only



For peer review only - http://bmjopen.bmj.com/site/about/guidelines.xhtml

## Search strategy (step 2)

The electronic searching was purposive and guided by the initial PT. A search strategy was developed which was piloted and adapted for MEDLINE until a diverse and relevant range of search results were yielded (target 500-3,000). Thus, there was no strict search "threshold" and the most important criterion for search results to meet was *relevance* [(22, 23)]. In line with a realist approach, searching was iterative, and the strategy was refined for each database (see *Supplementary file 3*). Sources included electronic databases, healthcare sites, and grey literature.

The search strategy was not intended to be exhaustive, but provided a large enough overview to be meaningful for PT development [(23)]. Evidence was searched up until September 2017; publications were monitored thereafter but no new evidence affected the PT. In total, 3113 documents were selected for screening.

## Selection and appraisal of documents (step 3)

Inclusion or exclusion of source evidence for the review were according to the following criteria:

**Inclusion criteria:**

- Full text or section of source had *relevance* [(22, 23)] to informing the PT
- Relate to inpatients/outpatients discharged from hospital to GP (or equivalent)
- Relate to discharge where discharge letter is sent to GP (may also be copied to patient)
- Source written or published in English

**Exclusion criteria:**

- Specific to discharge to units/physicians other than GPs (or equivalent), e.g. another hospital
- Specific to discharge of patients who lack cognitive capacity, e.g. dementia, or where there may be higher risk of harm, e.g. mental health discharge
- Lack of written communication having taken place, e.g. telephone only
- Specifically relate to patients <18 years
- Source not written or published in English

The criteria were developed to include evidence that encompassed a variety of patients and be relevant across different healthcare settings. The exclusion criteria posed limitations on the review; children under 18 (where the parent would often be the letter recipient), patients with particularly specialised communicative needs (e.g. patients without capacity) or where the intervention may have a higher potential risk of causing harm (e.g. psychiatric discharge documents) were excluded. The communication needs of some of these patients may be more complex and variable within and between groups and therefore was not possible within review scope. The first exclusion criterion states patient discharge communication to those other than GPs or family or community physicians was excluded. This is because the review specifically focussed on discharge communication to GPs and patients rather than referrals or care-handovers. Furthermore, the review aimed to develop a theory for patients receiving discharge communication and inclusion of hospital-hospital discharge may have reduced clarity and produced a less focussed theory.

Once KW had screened the documents by title and abstract, second reviewer EM screened a random 10% test selection; as recommended by Wong et al. [(26)]. Inter-reviewer agreement was set at kappa measure K≥.8 [(28)]. A result K<.8 would require all documents to be second screened. Inter-reviewer agreement was calculated as sufficient (K=0.82). In the first screening phase, 611 duplicates were removed and 2,341 documents excluded; this left 161 documents.



For peer review only - http://bmjopen.bmj.com/site/about/guidelines.xhtml

The full texts of these 161 documents were then screened, primarily for relevance [22, 23] by KW, with EM screening a random 10% sample. Inter-reviewer agreement was again high (K=0.92). Eighty eight documents were excluded at this stage leaving 73 for inclusion.

In addition, hand-searching of bibliographies, 'cited by' searching, and contacting experts was undertaken. This identified a further 30 relevant documents, creating a total of 103 documents. *Supplementary file 4* provides the final document list. The selection process is summarised in Figure 3.

*Figure 3 PRISMA [29] diagram (document selection process)*

**Data extraction and analysis (step 4)**

A "hybrid" [26, 30] approach to data extraction was undertaken. This allowed extraction of both descriptive document characteristics and annotation of CMOC ideas for synthesis and integration into the PT [22, 23]. The *Excel* data extraction form (see *Supplementary file 5*) was designed iteratively to record pertinent document details. Final columns included: author(s), year, geographical information, healthcare system, design aim, no. of participants intervention, clinical speciality, inclusion and exclusion criteria, findings/conclusions, rigour/quality assessment [22, 23], topic focus, form of discharge communication e.g. discharge summary, participant mix, staff mix, and relevance score [22-24].

Documents were also annotated in NVivo for CMOCs and PT ideas. Annotations were guided by the initial PT devised in step 1.

**Data synthesis (step 5)**

During step 5, data and annotations of PT ideas and CMOs were consolidated. A realist analytic approach, following the work of Pawson [22-24, 31], was used to interrogate the theory during data synthesis. Pawson [22-24, 31] presents several different frameworks for synthesising data evidence. We selected the framework [23] entitled "synthesis to consider the same theory in comparative settings", which involves five analytical strategy steps. This framework was chosen as it assumes theories sometimes "work" and "do not work" according to the particular setting; Pawson et al.[23] describe this as 'aim[ing] to make sense of the patterns of winners and losers'. Hence, this framework is suitable for the research questions which focus on cause and context of positive outcomes "winners" and negative outcomes "losers". Thus, data synthesis was grounded on the assumption that the intervention *outcomes* of the intervention may differ according to *context*.

The following realist analytical strategy steps [23] were undertaken simultaneously

1. Juxtaposition of data sources – align sources to build upon/clarify each other
2. Reconciliation of data discrepancies – explore reasons for data disparities
3. Adjudication of data – data quality consideration of trustworthiness/relevance
4. Consolidation of data - inference of Mechanisms for outcomes
5. Situation of evidence - consideration of intervention settings

'Juxtaposition of data sources' was achieved using NVivo 'nodes'. Annotations were labelled and coded as nodes. The nodes were named according to ideas or concepts around the PT and often formed groupings of mechanisms, outcomes and contexts. NVivo node coding resulted in 19 nodes seen in Table 1.



For peer review only - http://bmjopen.bmj.com/site/about/guidelines.xhtml

*Table 1 Coding nodes*

| Node name | No. of different sources coded | Total no. of sections of text coded |
|---|---|---|
| Autonomy | 5 | 5 |
| Clinician context (views) | 23 | 57 |
| Confidentiality | 12 | 15 |
| Context (when it does not work) | 29 | 46 |
| Context (when it does work) | 54 | 107 |
| Cost/resources | 20 | 33 |
| Dictate in front of patient | 3 | 5 |
| Doctor patient relationship | 5 | 7 |
| GP preference | 4 | 8 |
| NHS policy or contextual standards (international) | 30 | 51 |
| Outcomes (positive) | 58 | 128 |
| Outcomes (negative) | 22 | 28 |
| Patient as delivery method | 2 | 2 |
| Patient harm | 24 | 33 |
| Patient letters | 18 | 34 |
| Patient preference | 37 | 94 |
| Patient recall | 11 | 12 |
| Queries and contact | 10 | 12 |
| Understanding | 46 | 88 |

During, 'reconciliation of data discrepancies' [22, 23, 26] and 'adjudication of data' [22, 23, 26], NVivo was used for scanning and comparing data to identify disparities. Adjudicating and situating evidence was important to reconcile discrepancies [22, 23, 26]. Following node coding, a CMOC table was constructed (see *Supplementary file 6)* for consolidation of data and annotations. During table completion, we identified CMOCs where the intervention does and does not work.

## Programme theory refinement (step 6)

Review step 6 was to consider stakeholder perspectives to test and refine the PT in light of the synthesised data [23]. Stakeholder views are for "checking" that the PT aligns with real-life experiences [7]. We consulted three groups: local policy makers and health service commissioners, GPs and service-users/patients. These groups were selected due to accessibility and their differing roles. Groups were contacted through University links.



For peer review only - http://bmjopen.bmj.com/site/about/guidelines.xhtml

# RESULTS

## Document characteristics

The 103 evidence sources were from 16 countries across various continents with most emanating from England (54%), the US (17%), and Australia (7%). Healthcare settings were split between insurance style systems (23%) and publically funded systems (77%), such as the NHS. The date range of the sources was from 1979-2017 and the total number of participants detailed across the research studies was 16,383; this included staff and patient participants but there was not enough detail across all of the studies to quantify the participant type proportions. Most had been published in the 10 years prior to the search: 1970-1979 (1%), 1980-1989 (2%), 1990-1999 (7%), 2000-2009 (40%), and 2010-2017 (50%).

The evidence covered a wide range of specialties. Most specified inclusion of adult patients only (over 18 years) but often did not detail the exact patient ages in the write up; a few studies focussed on elderly patients. Information relating to patient demographics e.g. gender, was often not found in the sources and hence these were not summarised. Many sources instead focused on the speciality under consideration in the document and clinical presentations of interest to that speciality e.g. abnormal ECGs [(32)]. Participants who were staff included medical students, doctors of all training grades, nurses, GPs, non-specified hospital staff, and non-clinical staff. However, the majority of documents (66%) either did not provide staff participant details or they were irrelevant e.g. guideline document, no participants. The type of discharge communication that the evidence related to was varied: direct copies (48%), discharge instructions (13%), pictures (1%), personal discharge packs (1%), personalised letters (13%), information booklets (9%), multiple types of discharge communication (7%), and other (11%). Where the sources came from showed some variation such as Department of Health archive (3%) and conference listing (5%) but the greatest number of sources were from journals (68%).

## Quality and document rigour

The findings of this review in the following sections should be considered in light of the quality and rigour appraisal results of included documents to avoid over-interpretation of the findings. During data extraction in step four, documents were quality appraised for *rigour* and evaluated for *relevance* [(22, 23)]. The concept of *rigour* is defined as 'whether the methods used to generate the relevant data are credible and trustworthy' [(21)]. *Relevance* and *rigour* were scored on a scale from very low to very high and factors such as document type e.g. opinion piece or scientific trial paper, were considered. It is acknowledged that the appraisal process was subjective. Documents were not excluded solely based on rigour as extracts of documents with a lower quality score may still have valid contributions [(9)]. The full quality appraisal results are in the data extraction table (see *Supplementary file 5*).

The quality of evidence varied, with 53% of sources graded as medium or above for relevance and 80% for rigour. The remainder were graded medium/low (relevance=9%, rigour=18%), low (relevance=24%, rigour=1%) or very low (relevance=14%, rigour=1%). Information relating to setting and context was not always found or was insufficient. The source type was mixed: discussion pieces (20%), survey-based study (19%), guideline documents (13%), conference abstract (7%), review (5%), interview-based study (5%), experimental study (5%), pilot study (5%), randomised controlled trial or randomised intervention study (5%), non-randomised intervention study (3%), report document (3%), cohort study (2%), mixed methods (not covered above) (1%) and other e.g. PhD thesis (8%). Notably, the most common type was "discussion piece"; these were often solely based on the opinion of one individual and so did not always provide strong evidence.



For peer review only - http://bmjopen.bmj.com/site/about/guidelines.xhtml

In some areas, evidence relating to the PT were thin, these included: negative outcomes, intervention cost, recent clinician views, doctor-patient relationships, personalised patient letters and nil intervention. Thus, some CMOCs were constrained by source data. Evidence was also thin in relation to data disparities, for example the reasons *why* conflicting attitudes or results occurred were often not described. Although, context and outcome information was generally well supported, mechanisms were frequently omitted. Hence, where possible, the expertise of the research team was drawn on to infer reasons for disparities and what the likely mechanism(s) were within any CMOC. Hence, the CMOCs and PT presented in the following sections are based on source data where possible but have also been supported by stakeholder feedback and research team inferences.

**Context-mechanism-outcome configurations**

The following section provides an overview of theories in the form of a narrative of how patients receiving discharge letters does or does not work, as informed by the included sources. The sub-headed themes emerged during data interrogation and consolidation. Sections contain references to CMOCs, quotations from data texts, and references. Quotations have been chosen which illustrate the described theories and highlight key elements of CMOCs. The full table of 48 CMOCs is found in *Supplementary file 6*.

*Patient preference/choice*

Allowing patients to make their own choice for receiving letters may: reduce unnecessary resource strain [CMOC14], only take minimal time [12], make patients feel more involved in their care [2, 12, 33-37] [CMOC2], increase satisfaction [34, 38-42] [CMOC14, CMOC41, CMOC47] and aid information acceptance [43]:

> ‘‘*I wanted to know as much as possible about what was going on with my body*” [43](p.73)

> “*Sometimes for whatever reason you don't fully take on board what the doctor has told you. I found the letter useful to read over and digest properly what was written*" [2](p.3)

Many patients report that receiving letters is useful [2, 7, 36, 37, 39, 44-46]. Patients may show friends/family to help them better understand their condition/treatment [43, 47-51]. Patients may use letters as a reference/reminder for the consultation [42, 43]:

> *“My mind went blank after seeing the doctor and the letter reminded me of what had been said”* [36](p.83)

Across a range of specialties and settings, the reported patient preference for receiving copies of their discharge letter is generally high (79%-97%) [7, 11, 34, 35, 42-44, 46, 52-55]. However, not all patients may find letters helpful [40] or necessary [7, 34, 49], and some may not want to be reminded of their diagnosis [34], which could decrease satisfaction, and generate queries [56] if these patients were sent letters without a choice [CMOC40]. Hence, several studies argue in favour of respecting patient choice and suggest the patient’s right to “opt out” needs to be addressed [7, 38, 39, 43, 46, 47, 53, 57-59][CMOC41]. In situations where the patient is not offered a choice, such as third party information or risk of harm [2], the clinician should be able to justify this decision [60]. In relation to sensitive information or social diseases, patients generally do not object to this being included in the letter as long as it has ‘some relevance’ [60].

*Comprehension, queries and recall*

There was considerable evidence, particularly from patient viewpoints, to support the view that the majority of patients may understand their letters [7, 11, 33-36, 44-46, 50, 56, 60-64] and



For peer review only - http://bmjopen.bmj.com/site/about/guidelines.xhtml

hence find the letter beneficial and helpful [44, 45, 65] [CMOC7, CMOC39, CMOC44]. Moreover, a letter copy which is understood can reassure patients they are being listened to [44, 45, 65] [CMOC34, CMOC48]. Patient understanding of discharge instructions may increase their knowledge [49] and this might reduce unnecessary or avoidable hospital readmissions [1, 48, 64, 66-69], help patient acceptance of their illness [65], and reduce patient anxiety [70] thereby supporting patient wellbeing [47, 54, 60, 71] [CMOC39]:

> *"I found the letter very comforting and reassuring"* [72](p.58)

Although there may be a risk that patients receiving letters is associated with an increase in queries to seek clarification about what has been stated [33], several sources indicated that this occurs to a minimal extent [11, 33, 44, 57, 60] [CMOC29]. Examples of patients not understanding letters are often described as a "small proportion" [45] or low percentage '7%' [34].

If patients are provided verbal information only, they may fail to retain the information [36, 42] [CMOC18] which can decrease recall and adherence [CMOC21]:

> *"By the time I have got home, I have forgotten half of what was said in clinic."* [44](p.255)

Due to this, combining written and verbal information [73] may improve patient understanding [36, 51, 74-78] [CMOC15, CMOC18], increase patient's involvement in their care [36, 47] and compliance [46, 59] [CMOC11, CMOC43], and improve recall [40, 42, 43, 46, 56, 75, 79, 80] [CMOC5, CMOC15].

Contexts which may increase likelihood of triggering patient understanding include: letter language translation [38, 76, 81], writing the letter at a 5th or 6th grade reading age level (11 years) [16, 38, 75, 82] [CMOC12], use of glossary [47, 83], pictures, pictographs or equivalent [75, 84, 85] (particularly for low literacy or illiterate patients) [15, 48, 64, 66] [CMOC17], lay explanations for 'technical terms' [47, 60] and writing in plain English with minimal jargon/abbreviations [35, 39, 60, 75, 82, 86] [CMOC12].

A couple of sources suggested training doctors on writing patient letters can mean they produce letters more meaningful to patients [13, 72] [CMOC13]. However, the evidence around training in relation to the intervention was limited and needs further research.

*Personalised or patient-directed discharge letters*

Producing a letter which is comprehensible and useful to both GPs and patients has been recognised to be an issue [46]. Patient-directed or personalised patient letters have been proposed [CMOC24, CMOC36], and patients often rate these letters as 'helpful' [5, 49, 87] and that this may heighten satisfaction [49], and improve understanding [45]:

> *"Simplifying written communication has also been shown to improve patient comprehension."* [5](p.855)

However, personalised letters have the potential to lead to resource consumption [46] [CMOC25], staff time depletion [46, 88] and patient anxiety that they have been given different information to their GP [88] [CMOC26]. For these reasons, further research which weighs the benefits of personalised patient letters against the drawbacks and costs is needed.

*Patient to deliver letter*

The context of patients delivering letters seems to have few reported positive outcomes. Posting and electronic transferral of letters may be preferable as:



For peer review only - http://bmjopen.bmj.com/site/about/guidelines.xhtml

*"It is not considered good practice to send the discharge summary home with the patient as there is no guarantee that the information will be passed on to the general practitioner"* [89](p.7)[CMOC31].

*Dictating letters in front of patients*

Evidence for this concept was somewhat thin. One study suggested that dictating letters in front of patients can make patients feel less in need of a copy of the letter [11]. Another paper suggested this practice may also provide a context that triggers patients to challenge inaccuracies, improving letter quality [83] [CMOC22, CMOC30]:

"*The content of letters to GPs is sometimes incorrect and this may be remedied by dictating the letter in front of the patient."* [83]

*Confidentiality*

There are concerns and legal implications surrounding potential confidentiality breaches associated with patients receiving letters, particularly when they are sent out in the post [15, 35, 42, 49, 52, 54, 58, 90]. One recent paper [52] (2013), which looked at confidentiality, continued to stress risks around postal communication and the importance of secure information transfer:

"*There is a substantial risk of breaching patient confidentiality when distributing correspondence by post. A well-designed security arrangement is therefore required to ensure the safety of confidential information."* [52](p.35)

Some documents [52, 57, 58, 60, 91] suggested ways to reduce potential risk of confidentiality breach through communication platforms and the processes involved in sending letters e.g. verifying patient contact details before sending letter [60] [CMOC3, CMOC27, CMOC28].

*Patient harm*

Patient anxiety or "harm" in general are often cited as reasons for clinicians not wanting to copy letters, particularly in "bad news" settings [17, 36, 41, 43, 46, 47, 49, 56, 61, 92] [CMOC6]. Letter inaccuracies can cause concern leaving patients feeling confused or anxious [45] [CMOC19]. Nevertheless, the letter can reassure the patients their problems are being handled [54] and initial anxiety can settle or be nullified by the usefulness of the letter [44, 46, 49, 60, 93] [CMOC37]. Moreover, one study published in the *Lancet* in 1991 suggested patient letters in "bad news" settings may be more useful than "good news":

"*Patients who had received bad news found the letter significantly more useful in helping them to understand and remember what they had been told during the consultation than did patients receiving good news… almost half the patients receiving bad news found their letter distressing to some extent; however, with 1 exception, all patients were pleased to have received it."* [39] (Pp.924-925)

Although the above paper was published in 1991, there has been no recent evidence or system changes to dispute the notion that "bad news" letters may be of particular use to the patient. Hence, despite risk of initial "harm", "bad news" letters should perhaps not be avoided.

Practical and feasible suggestions were found in some documents for minimising harm or anxiety: not copying letters with information not previously disclosed to the patient [2, 43, 60] [CMOC38], abstain from use of value judgements e.g. pleasant lady [36], avoid copying letters where there are 'problems of privacy at home' and/or where the patient lacks capacity [2] [CMOC20], and checking the patient consents to a letter [60].

*Clinician views*



For peer review only - http://bmjopen.bmj.com/site/about/guidelines.xhtml

GP and hospital clinician views were described both as in favour [12, 33, 44, 49, 59, 65, 94] [CMOC5, CMOC16] and not in favour of patients receiving discharge letters across a range of specialities [33, 35, 36, 40, 44, 49, 50, 54, 65, 88, 95, 96] [CMOC6, CMOC35]. The response section [12, 14, 15] to a BMJ article [97] (2008) on patient letters demonstrates the clinician view dichotomy as two practitioners argue for and against patients receiving letters:

> "*Generally, doctors who are sceptical about copying letters to patients seem not to have tried it, whereas those who send copies routinely are enthusiastic."* [12](p.1370)

> "*My colleagues and I have had to explain to alarmed and bewildered patients who have received copies of their correspondence the meaning of phrases…"* [15](p.1369)

Practitioner perceived benefits found in the sources [CMOC5] included: improved patient understanding [49, 65, 88], increased transparency [50] [CMOC33], improved trust/doctor-patient relationship [12, 47, 49, 88], dispelling fears of 'secretive relationships' between clinicians and heightened sense of patient importance [49]. In addition, the patients' right to view the information was noted [88] [CMOC7, CMOC16]. A common practitioner concern of the intervention across specialties was letter comprehensibility and patient understanding [15, 33, 35, 36, 43, 47, 49, 50, 54, 88, 98, 99] [CMOC6]. Other concerns included: cost of additional materials/staff time [15, 33, 44, 45, 52, 88] [CMOC23, CMOC32], patient anxiety [47, 49] [CMOC6, CMOC19], increased patient queries [33] [CMOC29], potential confidentiality breaches [49] [CMOC6, CMOC27], and that letters would be oversimplified [49, 65, 88, 99]. An attitudinal issue found in two oncology papers [50, 88] published 17 years' apart was the view that letters are tools to be used between doctors only [CMOC6]. Additionally, juniors can learn from and mimic superiors and also not send letters to patients [100].

Confusion around 'letter comprehensibility' and lack of 'patient understanding' were the commonest clinician reservations relating to the intervention [15, 33, 35, 36, 43, 47, 49, 50, 54, 88, 98, 99]. However, as covered in the *comprehension* section, patients are often reported as *understanding* their letters [11, 33, 44, 57, 60] and furthermore they tend to express strong preference for receiving such letters [7, 11, 34, 35, 42-44, 46, 52-55]. Thus, it may be inferred from the evidence that patient understanding of letters is higher than clinicians' perceive. The following from a recent (2016) abstract concisely summarises an example of patient and clinician view disparity:

> "*While some oncologists assess the copy letters as inappropriate for supplemental patient-oncologist-communication, breast cancer patients regard this tool as predominantly gainful. Oncologists appear to stick to their traditional perspective which perceives the copy letter mainly as a communication tool from doctor to doctor*." [50] (p.185)

Notably, most of the evidence reporting clinician views was published from 2002-2008 and current evidence on clinician perspectives remains limited. Moreover, although sources often referred to conflicting clinician views, information on *why* attitudes differ was extremely thin. Overall, better understanding of current clinician views on copying discharge letters to patients is required. Further research should address reasons behind different viewpoints to include patients and practitioners.

*Cost and resources*

The estimated costs associated with the intervention varied [47] but this must be considered in the context that included documents spanned a wide time range and thus factors such as inflation need to be considered. In addition, robust health economic analyses were not found in the included sources. Documents [15, 33, 42, 54, 83] referred to "cost" or financial implications [CMOC25] of sending letters in different ways such as use of consumables [33, 45, 54, 55, 101][CMOC10], and secretarial [33, 44, 45, 54] [CMOC10] and clinician time required [45]. A few sources [34, 36, 44, 46, 55, 60, 102], including guideline documents and research papers, suggested that benefits were such that associated costs were minimal, or



For peer review only - http://bmjopen.bmj.com/site/about/guidelines.xhtml

even reduced by patients being more informed from receiving discharging communication [CMOC7, CMOC25, CMOC42]. However, as these views were based on personal comment or studies with weak methodologies, the true cost consequences remain unknown.

*Autonomy*

One source suggested that when patients are not given letters, they may feel less involved in their care, resulting in reduced patient autonomy [103] [CMOC1, CMOC6]:

> *"To refuse to provide such information if this is the patient's wish is to deny their autonomy."* [103] (p.388)

Conversely, some evidence was found that providing patients with written discharge letters is their "right" [3, 58], may create a sense of involvement, and increase patient autonomy and satisfaction [103, 104] [CMOC2, CMOC4, CMOC5, CMOC8, CMOC14].

*Doctor-patient relationships*

Very few documents were found which considered the intervention in terms of the doctor-patient relationship. However, the limited evidence that was found indicated that patients receiving letters has the potential to improve communication, trust and the doctor-patient relationship [CMOC9] [33, 43, 47].

**Stakeholder perspectives**

As detailed in step 6, the final review step was to refine the programme theory through stakeholder perspectives. Three groups were consulted: local commissioners, GPs and service-users/patients. Groups were relatively small and not representative of the general population; due to feasibility it was not possible to achieve diverse and representative group samples.

All group discussions were centred on the programme theory; members were encouraged to critique and feedback on the PT diagram. Between all the groups, concepts not covered or explored in adequate detail in the PT diagram were raised in regards to: the importance of comprehensible language and terminology, difficulty and problems retaining verbal information only e.g. where anaesthesia used, patient choice of receiving letters, illegibility of handwritten discharge communication, critical context of prior patient communication of a high quality to increase likelihood of understanding discharge letters, issues around personalised patient letters considering NHS resource availability, and concerns around writing a letter which meets the needs of both GP and patient. The commissioner and GP representatives also emphasised the importance of patient safety and that this should be central to best practice recommendations. In addition, the patient group reported reading a letter about themselves written in third person was peculiar. The patient group felt patient letters were very important for patients taking responsibility for their health in line with the NHS promotion of patient-centred and patient-led care.

Several different members across the various groups commented that in practice, patients do not always receive their letters, despite this process being best practice. Recommendations were suggested to rectify this by the commissioner members to include: clinicians should assume when writing letters that they could be made available to the patient, early clinician and student training in good letter writing and record keeping, and that hospitals should support the initiative e.g. audits.



For peer review only - http://bmjopen.bmj.com/site/about/guidelines.xhtml

## Cycling of review steps

As a realist review is an iterative process, following the initial six step cycle, steps may be repeated in order to attain “theoretical saturation” [22, 24]. There was substantial overlap between documents which is demonstrated by the fact there are 103 documents in the review but only 48 CMOCs. Furthermore, as described in step two, new publications were followed and consulted for evidence and provided no new or conflicting programme theory knowledge. Thus, it was deemed that “theoretical saturation” in accordance with Pawson’s realist review methodology [22-25] was attained and no further searching or step cycling was required.

## Resultant programme theory

The PT was systematically updated and finalised following review steps 1-6 (Figure 4). Thus, several changes were made to the initial theory (Figure 2) to produce the resultant theory below (Figure 4) and these are summarised here.

The feasibility of providing a personal patient letter with current NHS setting of resource provision was removed from the PT. Therefore, the personalised letter section of the PT instead focuses on alternative healthcare settings e.g. insurance-based, which is the setting where the majority of research on this type of intervention was conducted. In addition, the box of contextual influences was deleted, and the points integrated into the overall diagram. Patient outcomes throughout the PT were simplified and clarified, for example, the outcomes such as ‘empowered patients’ and  ‘reduce patient anxiety’ were simplified to the outcome ‘improved patient well-being’. The final PT has more refined and distinct pathways for when the patient does and does not receive letters and the subsequent respective outcomes. Furthermore, contexts for when the patient *does* receive their letter(s) were condensed into an aligned grouping of five key contexts for when the intervention may be theorised to *work* and four key contexts for when the intervention may be theorised *not to work.* In the resultant PT, CMOCs have been “grouped” where overlap was apparent, for example, all resources are labelled simply as “resources” as most of the data concurrently referred to financial, administrative and clinician time resources.

The resultant theory is an overview of all the CMOCs identified in this review but is acknowledged that the below diagram is unlikely to be entirely comprehensive.



For peer review only - http://bmjopen.bmj.com/site/about/guidelines.xhtml

*Figure 4 Resultant Programme Theory*



For peer review only - http://bmjopen.bmj.com/site/about/guidelines.xhtml

## DISCUSSION

### Statement of principal findings

This review of 103 sources summarises and expands upon existing evidence by moving beyond "benefits" and "drawbacks" of patients receiving letters alone, and considering contexts of *when* as well as *how* the intervention works. Although the review focuses on the UK health system, our use of realist review has enabled identification of findings that may be transferable to other healthcare settings.

RQ1 asked about positive and negative outcomes of the intervention. Positive outcomes include: increased patient satisfaction [34, 38-42], improved doctor-patient relationship and trust [33, 43, 47], heightened patient knowledge [32, 75], improved letter and record quality [2, 13, 33, 45], and reduced anxiety [17]. Negative outcomes include patient queries [47], confusion [33, 49, 54, 61], and anxiety [47, 49]. RQ2 enquired after the important contexts for triggering these outcomes. Important contexts for positive outcomes include: letters written in plain English with minimal abbreviations [60], lay explanations or simplified terms in brackets for medical jargon e.g. myocardial infarction (heart attack) [47, 60], written information provided alongside verbal explanation, no new information in letter [2] or value judgements [36], letter translation [38, 76, 81] where relevant, training clinicians on letter writing practice [13, 72], use of pictures and glossaries where relevant [75, 84, 85], letters only given to patients who choose to have them [47, 60], and where there is no identified risk of harm [2] or confidentiality breach [60].

This review has produced two key findings, which are important but not wholly surprising. The first is that the reviewed evidence indicates that patient "understanding" of their discharge letters is greater than clinicians perceive [7, 11, 15, 33-36, 43-47, 49, 50, 56, 60-64, 88, 98, 99]. However, reasons behind patient and clinician perceived comprehension discrepancies were unclear. It is important to situate the first finding in terms of the study exclusion criteria and participant diversity across the evidence reviewed, for example, it is likely that patients who participate in research on this topic have a greater level of interest and literacy than those who did not participate. One or a number of demographic groups not involved in the studies, either by choice not to participate or by exclusion, may have accounted for a portion of those who clinicians perceive to have low understanding. Thus, evidence for low patient understanding was lacking, and this requires further research. The second key finding is that in a number of contexts, patients expressed preference for receiving correspondence [7, 11, 34, 35, 42-44, 46, 52-55]. Patients can continue to use the letter(s) to refer to beyond discharge [34, 93] as a medication list reminder, and to share with friends/relatives as desired [34, 81]. Nevertheless, patient *choice* should still be acknowledged as the review did find evidence that not *all* patients want their letters; a practical way of addressing this would be to check with the patient that they want a letter in the first instance [7, 34, 40, 47, 49, 60].

Systems for monitoring patient letters, e.g. the Newcastle Trust Policy for auditing and sharing letters with patients [60] seems prudent moving forward [17]. This is of particular relevance in the NHS given that guidelines for copying letters have been widely available since 2003 [2] and yet in practice, many patients do not receive letters [42, 47, 52, 79, 105]. Given the wider context of a drive for patient-led care and patient-centred communication and decision-making [103], this review is timely and relevant. The review findings have the potential to influence policy and improve practice. The results demonstrate how care can be improved through patient choice and good quality letter provision. However, current clinician views [33, 35, 36, 44, 49, 50, 65, 88] and hierarchical mimicking of practices of seniors [100] pose a barrier [40] to implementation and need addressing.

### Review limitations

For this review we followed the RAMESES quality and publication standards for realist reviews [106, 107]. Quality assessment and analysis is to a degree dependent on



For peer review only - http://bmjopen.bmj.com/site/about/guidelines.xhtml

reviewer skills and reflexivity [108, 109]. Furthermore, analysis and inferences were ‘subjective and interpretative’ [110, 111]. However, because the steps we have taken for this review are transparent, other review teams can see and make judgements on result plausibility.

The resultant PT is limited by the quality and content of evidence reviewed. Some of the evidence found in sources was markedly thin, particularly in relation to costing information, recent clinician viewpoints, personalised letter copies, and influence on the doctor-patient relationship. Furthermore, there were a greater number of CMOCs relating to positive outcomes than negative outcomes, that is, when the intervention *does* work than *doesn't.* This may be rationalised by publication bias towards positive findings. Similarly, CMOCs for patients *not* receiving letters (nil intervention) were thin. Consequently, these evidence limitations constrained the detail available in the resultant PT in these areas.

The review is also limited by the inclusion and exclusion criteria; not all patient groups could be considered. Furthermore, the PT is limited by the representativeness of patient groups within sources. The usefulness of the programme theory to a general health population is affected by the participation bias; certain groups are more and less likely to participate and ethnic minorities, illiterate patients and other hard to reach groups are likely under-represented. It is essential to take this into account when considering the weight of the review recommendations for informing best practice of discharge communication.

It is hoped the PT may be used as a starting point for future research and be useful and practicable for informing policy and guidelines. It is acknowledged that the review is not exhaustive. However, this is not the intention of a realist review [112].

**Suggestions for future research**

Further research is needed to define the cost benefits of copying patients into discharge letters. Potential barriers such as clinician views and the current limited available clinician training on letter writing should be addressed. Since patient and clinician views were sometimes conflicting, a study which parallels both views alongside the same patient cases to understand reasons for any discrepancies would be useful and may provide valuable insights. Furthermore, the PT would benefit from further refinement given the identified limitations and in particular from more data from alternative settings.

## CONCLUSION

The resultant PT forms a basis for explaining how, when, why and for whom this intervention does and does not work. The resultant PT makes suggestions for how best practice of patients receiving discharge letters may be improved, although it should be considered that evidence for some aspects of the PT was lacking.

The review's key findings are that patient understanding is possibly greater than clinicians' perceive, patient choice is instrumental to increasing likelihood of desired patient outcomes, and clinician views of the intervention is a barrier to practice implementation. This barrier could be addressed through clinician training and organisational initiatives which guide, mandate, and monitor the intervention. Without hospital organisational support, it is unlikely this practice will be consistently adopted into practice given the barriers identified in the review. Given the drive for patient–centred care, involving patients in their healthcare communication is not something that should be applied inconsistently.

In conclusion, this review outlines how the intervention of patients receiving their discharge letters may *work* to increase the likelihood of positive effects.



For peer review only - http://bmjopen.bmj.com/site/about/guidelines.xhtml

For peer review only

**Author contributions:** KW was the lead reviewer. EM completed the second reviewer tasks. All authors contributed toward the programme theory through discussions. KW is responsible for the design and drafting of the initial manuscript. GW, ES, SS and JD critically reviewed and edited the final manuscript. All authors read and approved the final manuscript.

**Acknowledgements:** Samantha Johnson, our information specialist, for critically appraising the search strategy.

**Funding statement:** This work is supported by the Economic and Social Research Council (ESRC) and Clinical Commissioning Groups (CCGs) of Coventry & Rugby and South Warwickshire. Funding for the open access charges for publication of this protocol was provided by the RCUK fund.

**Competing interests:** None known

## References

1.NHS England. Standards for the Communication of Patient Diagnostic Test Results on Discharge from Hospital. 2016 https://improvement.nhs.uk/uploads/documents/discharge-standards-march-16.pdf.
2.*Department of Health*. Copying letters to patients: good practice guidelines. 2003 http://webarchive.nationalarchives.gov.uk/.
3.*Department of Health*. The NHS Plan. 2003 http://webarchive.nationalarchives.gov.uk. .
4.The Academy of medical Royal Colleges. Please, write to me: Writing outpatient clinic letters to patients. *AOMRC*. 2018 https://www.aomrc.org.uk/reports-guidance/please-write-to-me-writing-outpatient-clinic-letters-to-patients-guidance/.
5.Lin R., Gallagher R., Spinaze M., Najoumian H., Dennis C., Clifton-Bligh R., Tofler G. Effect of a patient-directed discharge letter on patient understanding of their hospitalisation. *Internal medicine journal*. 2014;44(9):851-7.
6.Santana M.J., Holroyd-Leduc J., Flemons W.W., O'Beirne M., White D., Clayden N., Forster A.J., Ghali W.A. The seamless transfer of care: a pilot study assessing the usability of an electronic transfer of care communication tool. *American Journal of Medical Quality*. 2014;29(6):476-83.
7.O'Driscoll B.R., Koch J., Paschalides C. Copying letters to patients: Most patients want copies of letters from outpatient clinics and find them useful. *Bmj*. 2003;327(7412):451.
8.White P. Copying referral letters to patients: prepare for change. *Patient Education & Counseling*. 2004;54(2):159-61.
9.Marzanski M., Musunuri P., Coupe T. Copying letters to patients: A study of patient's views. *Psychiatric Bulletin*. 2005;29(2):56-8.
10.Minhas R. Does copying clinical or sharing correspondence to patients result in better care? *International Journal of Clinical Practice*. 2007;61(8):1390-5.
11.Pothier D.D., Nakivell P., Hall C.E. What do patients think about being copied into their GP letters? *Annals of the Royal College of Surgeons of England*. 2007;89(7):718-21.
12.Shee C.D. Try it and see. *Bmj*. 2008;337:a2786.
13.Thornber M. Copy them in. *Bmj*. 2008;337.
14.Main J. Copying in or copping out? *Bmj*. 2008;337:a2688.
15.McKinstry B. Copying patients in is not as simple as it seems. *Bmj*. 2008;337:a2687.
16.Choudhry A.J., Baghdadi Y.M., Wagie A.E., Habermann E.B., Heller S.F., Jenkins D.H., Cullinane D.C., Zielinski M.D. Readability of discharge summaries: with what level of information are we dismissing our patients? *American Journal of Surgery*. 2016;211(3):631-6.



For peer review only - http://bmjopen.bmj.com/site/about/guidelines.xhtml

17. Boaden R., Harris C. Copying letters to patients—will it happen? : Oxford University Press; 2005. Available from https://academic.oup.com/fampra/article/22/2/141/522310
18. Weetman K., Wong G., Scott E., Schnurr S., Dale J. Improving best practice for patients receiving hospital discharge letters: a realist review protocol. *BMJ Open*. 2017;7(11).
19. Harris C., Boaden R. Copying letters to patients: the view of patients and health professionals. *J Health Serv Res Policy*. 2006;11(3):133-40.
20. Minhas R. Does copying clinical or sharing correspondence to patients result in better care? *Int J Clin Pract*. 2007;61(8):1390-5.
21. Brennan N., Bryce M., Pearson M., Wong G., Cooper C., Archer J. Understanding how appraisal of doctors produces its effects: a realist review protocol. *BMJ Open*. 2014;4(6).
22. Pawson R., Greenhalgh T., Harvey G., Walshe K. Realist review--a new method of systematic review designed for complex policy interventions. *J Health Serv Res Policy*. 2005;10 Suppl 1:21-34.
23. Pawson R., Greenhalgh, T., Harvey, G. & Walshe, K. . Realist synthesis: an introduction.' *ESRC Research Methods Programme*. 2004 Available at: https://goo.gl/1Rz2Ry.
24. Pawson R. Digging for nuggets: how 'bad' research can yield 'good' evidence. *International Journal of Social Research Methodology*. 2006;9(2):127-42.
25. Pawson R., editor Middle range theory and program theory evaluation: From provenance to practice. Transaction Publishers; 2010.
26. Wong G., Brennan N., Mattick K., Pearson M., Briscoe S., Papoutsi C. Interventions to improve antimicrobial prescribing of doctors in training: the IMPACT (IMProving Antimicrobial presCribing of doctors in Training) realist review. *BMJ Open*. 2015;5(10).
27. Ford J.A., Wong G., Jones A.P., Steel N. Access to primary care for socioeconomically disadvantaged older people in rural areas: a realist review. *BMJ Open*. 2016;6(5):e010652.
28. Centre for Reviews and Dissemination. Systematic Reviews: CRD's guidance for undertaking reviews in health care. 2008 https://www.york.ac.uk/media/crd/Systematic_Reviews.pdf.
29. Moher D., Liberati A., Tetzlaff J., Altman D.G. Preferred reporting items for systematic reviews and meta-analyses: the PRISMA statement. *Bmj*. 2009;339.
30. Wiese A., Kilty C., Bergin C., Flood P., Fu N., Horgan M., Higgins A., Maher B., O'Kane G., Prihodova L., Slattery D., Bennett D. Protocol for a realist review of workplace learning in postgraduate medical education and training. *Systematic Reviews*. 2017;6:10.
31. Pawson R. *Evidence-based policy: a realist perspective*: Sage; 2006.
32. Ackermann S., Heierle A., Bingisser M.B., Hertwig R., Padiyath R., Nickel C.H., Langewitz W., Bingisser R. Discharge Communication in Patients Presenting to the Emergency Department With Chest Pain: Defining the Ideal Content. *Health Communication*. 2016;31(5):557-65.
33. Brockbank K. Copying patient letters - Making it work. *Clinical Governance*. 2005;10(3):231-40.
34. Krishna Y., Damato B.E. Patient attitudes to receiving copies of outpatient clinic letters from the ocular oncologist to the referring ophthalmologist and GP. *Eye (Lond)*. 2005;19(11):1200-4.
35. Treacy K., Elborn J.S., Rendall J., Bradley J.M. Copying letters to patients with cystic fibrosis (CF): letter content and patient perceptions of benefit. *J Cyst Fibros*. 2008;7(6):511-4.
36. Tomkins C.S., Braid J.J., Williams H.C. Do dermatology outpatients value a copy of the letter sent to their general practitioner? In what way and at what cost? *Clinical & Experimental Dermatology*. 2004;29(1):81-6.
37. Perkins P., Jordan A., Prentice W., Regnard C. Copying letters to patients: a survey of patients and GPs views. *Palliative medicine*. 2007;21(4):355-6.



For peer review only - http://bmjopen.bmj.com/site/about/guidelines.xhtml

38.Advancing effective communication, cultural competence, and patient- and family-centered care : a roadmap for hospitals. *Joint Commission*. 2014 http://www.jointcommission.org/assets/1/6/aroadmapforhospitalsfinalversion727.pdf.
39.Damian D., Tattersall M.H. Letters to patients: improving communication in cancer care. *Lancet*. 1991;338(8772):923-5.
40.Saunders N.C., Georgalas C., Blaney S.P., Dixon H., Topham J.H. Does receiving a copy of correspondence improve patients' satisfaction with their out-patient consultation? *Journal of Laryngology & Otology*. 2003;117(2):126-9.
41.Menon G.J., Dutton G.N. Writing to our patients. *British Journal of Ophthalmology*. 1999;83(7):765.
42.Antoniou A., Saunders M., Bourner R., Crouch L. would you like to see yours? *The Bulletin of the Royal College of Surgeons of England*. 2007;89(2):62-4.
43.Fenton C., Al-Ani A., Trinh A., Srinivasan A., Marion K., Hebbard G. Impact of providing patients with copies of their medical correspondence: a randomised controlled study. *Internal medicine journal*. 2017;47(1):68-75.
44.Nixon J., Courtney P. Copying clinic letters to patients. *Rheumatology*. 2005;44(2):255-6.
45.Pinder E., Jefferys S., Loeffler M. Patient Satisfaction: Receiving a copy of the GP letter following fracture or elective orthopaedic clinic. *BMJ Qual Improv Rep*. 2013;2(2).
46.Brodie T., Lewis D. A survey of patient views on receiving vascular outpatient letters. *European Journal of Vascular and Endovascular Surgery*. 2010;39(1):5-10.
47.Baxter S., Farrell K., Brown C., Clarke J., Davies H. Where have all the copy letters gone? A review of current practice in professional-patient correspondence. *Patient Education & Counseling*. 2008;71(2):259-64.
48.Regalbuto R., Maurer M.S., Chapel D., Mendez J., Shaffer J.A. Joint Commission requirements for discharge instructions in patients with heart failure: is understanding important for preventing readmissions? *Journal of Cardiac Failure*. 2014;20(9):641-9.
49.O'Reilly M., Cahill M.R., Perry I.J. Writing to patients: a randomised controlled trial. *Clinical Medicine*. 2006;6(2):178-82.
50.Baumann W., Schussler L., Bertram M., Benser J., Kumpers S., Hermes-Moll K. Oncologists' letters for breast cancer patients. *Oncology Research and Treatment*. 2016;39:184-5.
51.Sandler D.A., Heaton C., Garner S.T., Mitchell J.R. Patients' and general practitioners' satisfaction with information given on discharge from hospital: audit of a new information card. *Bmj*. 1989;299(6714):1511-3.
52.Somov P., Madden T., Wong K., Hamm R. Security Concerns About Copying Clinical Letters to Patients. *The Bulletin of the Royal College of Surgeons of England*. 2013;95(1):33-4.
53.Rao M., Fogarty P. What did the doctor say? *Journal of Obstetrics & Gynaecology*. 2007;27(5):479-80.
54.Jelley D., van Zwanenberg T., Walker C. Copying letters to patients : Concerns of clinicians and patients need to be addressed first. *Bmj*. 2002;325(7376):1359.
55.Sharma D., O'Brien S., Hardy K. Copying letters to patients: What patients think - A questionnaire survey. *Clinician in Management*. 2007;15(2):75-8.
56.Hallowell N. Providing letters to patients. Patients find summary letters useful. *Bmj*. 1998;316(7147):1830.
57.Lepping P., Paravastu S.C., Turner J., Billings P., Minchom P. Copying GP letters to patients: a comprehensive study across four different departments in a district general hospital. *Health informatics journal*. 2010;16(1):58-62.
58.Singh S., Budeda B., Housden P. Do patients want copies of their GP letters?–our experience with 7250 patients. *International journal of clinical practice*. 2007;61(8):1407-9.
59.Smith P.E.M. Letters to patients: sending the right message. *Bmj*. 2002;324(7338):685.
60.The Newcastle upon Tyne Hospitals NHS Foundation Trust. The Newcastle upon Tyne Hospitals NHS Foundation Trust: Sharing Letters with Patients Policy. 2013.



For peer review only - http://bmjopen.bmj.com/site/about/guidelines.xhtml

61.Dooher P., Syed A., Liu J., Chopra A., Bradpiece H., Jenkins S., Patel A. Copying letter to patients-distress or satisfaction? *European Journal of Cancer*. 2012;48:S151.
62.Synthesis and conceptual analysis of the SDO programme's research on continuity of care. *National Institute for Health Research (NIHR)*. 2010 http://www.netscc.ac.uk/hsdr/files/project/SDO_FR_08-1813-248_V01.pdf.
63.Lin M.J., Tirosh A.G., Landry A. Examining patient comprehension of emergency department discharge instructions: Who says they understand when they do not? *Internal & Emergency Medicine*. 2015;10(8):993-1002.
64.Lorenzati B., Quaranta C., Perotto M., Tartaglino B., Lauria G. Discharge communication is an important underestimated problem in emergency department. *Internal & Emergency Medicine*. 2016;11(1):157-8.
65.Bench S., Day T., Griffiths P. Effectiveness of critical care discharge information in supporting early recovery from critical illness. *Critical Care Nurse*. 2013;33(3):41-52.
66.Saidinejad M., Zorc J. Mobile and web-based education: delivering emergency department discharge and aftercare instructions. *Pediatric Emergency Care*. 2014;30(3):211-6.
67.Verhaegh K.J., Buurman B.M., Veenboer G.C., de Rooij S.E., Geerlings S.E. The implementation of a comprehensive discharge bundle to improve the discharge process: a quasi-experimental study. *Netherlands Journal of Medicine*. 2014;72(6):318-25.
68.HPOE: A Compendium of Implementation Guides 2011. 2011 http://www.hpoe.org/Reports-HPOE/hpoe_compendium_2011.pdf.
69.Cannaby A.-M. *Improving the process of hospital discharge for medical patients* [Ph.D.]. Ann Arbor: University of Leicester (United Kingdom); 2003.
70.Ackermann S., Bingisser M.B., Heierle A., Langewitz W., Hertwig R., Bingisser R. Discharge communication in the emergency department: physicians underestimate the time needed. *Swiss Medical Weekly*. 2012;142:w13588.
71.Charlett S.D., Bajaj Y., Kelly G. Writing to patients with the results of routine tests: A measure to improve access to outpatient clinics. *Otorhinolaryngologist*. 2009;2(3):73-4.
72.Mrduljas-Djujic N., Pavlicevic I., Marusic A., Marusic M. Students letters to patients as a part of education in family medicine. *Acta Medica Academica*. 2012;41(1):52-8.
73.Generic Standards. 2002 http://www.healthcareimprovementscotland.org/his/idoc.ashx?docid=ea8d290a-9504-4c21-a889-fc479d530a51&version=-1.
74.Exploring patient participation in reducing health-care-related safety risks. 2013 http://www.euro.who.int/__data/assets/pdf_file/0010/185779/e96814.pdf.
75.Taylor D.M., Cameron P.A. Discharge instructions for emergency department patients: What should we provide? *Journal of Accident and Emergency Medicine*. 2000;17(2):86-90.
76.Perera K.Y., Ranasinghe P., Adikari A.M., Balagobi B., Constantine G.R., Jayasinghe S. Medium of language in discharge summaries: would the use of native language improve patients' knowledge of their illness and medications? *Journal of Health Communication*. 2012;17(2):141-8.
77.Carol Lim K.K., Chan S.K., Chew E.L., Anita Lim A.F., Sararaks S., Ainul H., Roslinah A., Tan L.S., Low L.L., Azman A.B., Maimunah A.H. Handoff communication - Let's do it right. *Medical Journal of Malaysia*. 2010;65:8.
78.Reilly M.M. Let's set the record straight: preparing the discharge summary and the patient's instruction sheet. *Nursing*. 1979;9(1):56-61.
79.Hoek A.E., De Ridder M.A., Bayliss A., Patka P., Rood P.P. Effective strategy for improving instructions for analgesic use in the emergency department. *European Journal of Emergency Medicine*. 2013;20(3):210-3.
80.Sandler D.A., Mitchell J.R., Fellows A., Garner S.T. Is an information booklet for patients leaving hospital helpful and useful? *Bmj*. 1989;298(6677):870-4.



For peer review only - http://bmjopen.bmj.com/site/about/guidelines.xhtml

81.Wimsett J., Harper A., Jones P. Review article: Components of a good quality discharge summary: a systematic review. *Emergency Medicine Australasia*. 2014;26(5):430-8.
82.Reddick B., Holland C. Reinforcing discharge education and planning. *Nursing Management*. 2015;46(5):10-4.
83.Roberts N.J., Partridge M.R. How useful are post consultation letters to patients? *BMC Medicine*. 2006;4:2-.
84.Fayers T., Abdullah W., Walton V., Wilkins M.R. Impact of written and photographic instruction sheets on patient behavior after cataract surgery. *Journal of Cataract & Refractive Surgery*. 2009;35(10):1739-43.
85.Zeng-Treitler Q., Kim H., Hunter M. Improving patient comprehension and recall of discharge instructions by supplementing free texts with pictographs. *AMIA* 2008;Annual Symposium Proceedings/AMIA Symposium.:849-53.
86.Samuels-Kalow M., Rhodes K., Uspal J., Reyes Smith A., Hardy E., Mollen C. Unmet Needs at the Time of Emergency Department Discharge. *Academic Emergency Medicine*. 2016;23(3):279-87.
87.Lin R., Tofler G., Spinaze M., Dennis C., Clifton-Bligh R., Nojoumian H., Gallagher R. Patient-directed discharge letter (PADDLE)-a simple and brief intervention to improve patient knowledge and understanding at time of hospital discharge. *Heart Lung and Circulation*. 2012;21:S312.
88.McConnell D., Butow P., Tattersall M. Audiotapes and letters to patients: the practice and views of oncologists, surgeons and general practitioners. *British Journal of Cancer*. 1999;79(11-12):1782.
89.Guidelines on regional immediate discharge documentation for patients being discharged from secondary into primary care. *Guidelines and Audit Implementation Network (GAIN)*. 2011 https://www.rqia.org.uk/RQIA/files/73/734a792f-f9d4-47f0-830f-31f9db51c82a.pdf.
90.McEniry B., Pillay I. How do patients in a rural setting respond to receiving a copy of their general practitioners letter? *Ir Med J*. 2008;101(3):84-5.
91.Polster D. Patient discharge information. *Nursing*. 2015;45(5):42-9.
92.Bench S., Day T., Heelas K., Hopkins P., White C., Griffiths P. Evaluating the feasibility and effectiveness of a critical care discharge information pack for patients and their families: a pilot cluster randomised controlled trial. *BMJ Open*. 2015;5(11):e006852.
93.Mrduljas Dujic N., Zitnik E., Pavelin L., Bacic D., Boljat M., Vrdoljak D., Pavlicevic I., Dvornik A., Marusic A., Marusic M. Writing letters to patients as an educational tool for medical students. *BMC Medical Education*. 2013;13:114.
94.Adams D.C., Bristol J.B., Poskitt K.R. Surgical discharge summaries: improving the record. *Annals of the Royal College of Surgeons of England*. 1993;75(2):96-9.
95.Partridge M.R., Roberts N.J. Writing to patients. *Clinical Medicine*. 2006;6(3):319.
96.Reilly M.O., Cahill M., Perry I.J. Writing to patients: 'putting the patient in the picture'. *Ir Med J*. 2005;98(2):58-60.
97.Richards T. Copy them in. *Bmj*. 2008;337.
98.Buurman B.M., Verhaegh K.J., Smeulers M., Vermeulen H., Geerlings S.E., Smorenburg S., de Rooij S.E. Improving handoff communication from hospital to home: the development, implementation and evaluation of a personalized patient discharge letter. *International Journal for Quality in Health Care*. 2016;28(3):384-90.
99.Todhunter S.L., Clamp P.J., Gillett S., Pothier D.D. Readability of out-patient letters copied to patients: can patients understand what is written about them? *J Laryngol Otol*. 2010;124(3):324-7.
100.Paravastu S., Lepping P., Billings P. Copying clinic letters to surgical patients. *The Bulletin of the Royal College of Surgeons of England*. 2007;89(8):288-90.
101.Tattersall R. Writing for and to patients. *Diabetic Medicine*. 1990;7(10):917-9.
102.Rao P., Andrei A., Fried A., Gonzalez D., Shine D. Assessing quality and efficiency of discharge summaries. *American Journal of Medical Quality*. 2005;20(6):337-43.
103.Chantler C., Johnson J. Patients should receive copies of letters and summaries. *Bmj*. 2002;325(7360):388.



For peer review only - http://bmjopen.bmj.com/site/about/guidelines.xhtml

104. Vaidya G. Copying letters to patients: Are we ready yet? *Hospital Medicine*. 2004;65(8):454-5.
105. Discharge planning : best practice in transitions of care. *The Queen's Nursing Institute*. 2016 https://www.qni.org.uk/wp-content/uploads/2016/09/discharge_planning_report_2015.pdf.
106. RAMSES. Ramses Guidelines. 2013 http://www.ramesesproject.org/.
107. Wong G., Greenhalgh T., Westhorp G., Buckingham J., Pawson R. RAMESES publication standards: realist syntheses. *BMC Medicine*. 2013;11(1):21.
108. Rycroft-Malone J., Burton C., Hall B., McCormack B., Nutley S., Seddon D., Williams L. Improving skills and care standards in the support workforce for older people: a realist review. *BMJ Open*. 2014;4(5).
109. Rycroft-Malone J., McCormack B., Hutchinson A.M., DeCorby K., Bucknall T.K., Kent B., Schultz A., Snelgrove-Clarke E., Stetler C.B., Titler M. Realist synthesis: illustrating the method for implementation research. *Implementation Science*. 2012;7(1):33.
110. Wong G., Greenhalgh T., Pawson R. Internet-based medical education: a realist review of what works, for whom and in what circumstances. *BMC Medical Education*. 2010;10(1):12.
111. McMahon T., Ward P.R. HIV among immigrants living in high-income countries: a realist review of evidence to guide targeted approaches to behavioural HIV prevention. *Systematic Reviews*. 2012;1:56.
112. Wong G. The Internet in Medical Education: A Worked Example of a Realist Review. *Synthesizing Qualitative Research* http://dx.doi.org/10.1002/9781119959847.ch5: John Wiley & Sons, Ltd; 2011. p. 83-112.



For peer review only - http://bmjopen.bmj.com/site/about/guidelines.xhtml

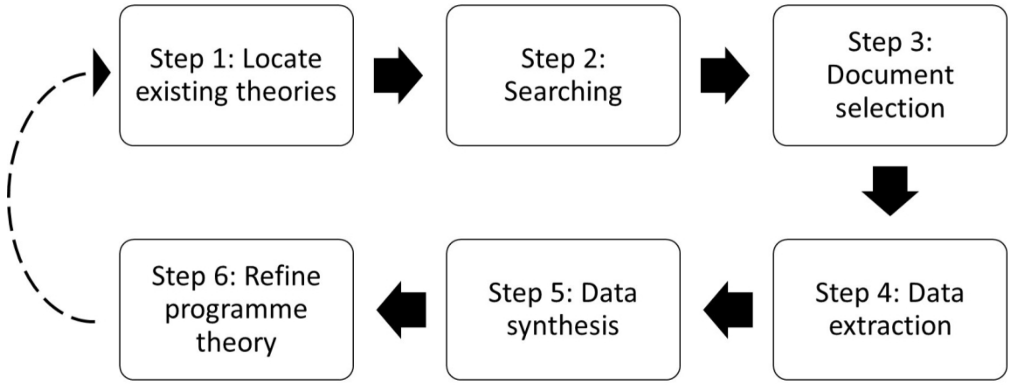
Step 1: Locate existing theories
Step 2: Searching
Step 3: Document selection
Step 4: Data extraction
Step 5: Data synthesis
Step 6: Refine programme theory

Figure 1

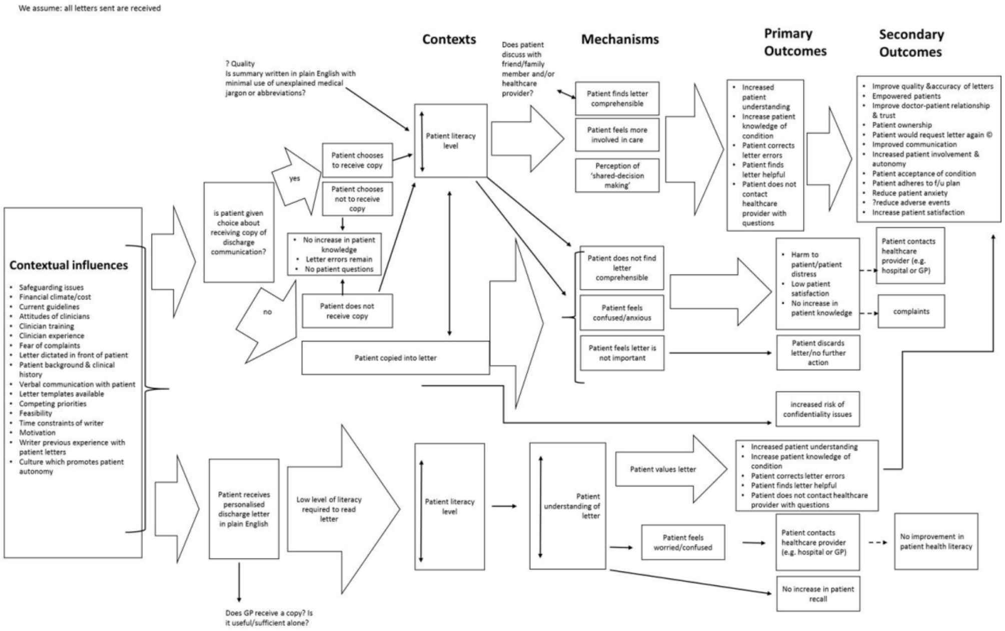
We assume: all letters sent are received
Contexts
Mechanisms
Primary Outcomes
Secondary Outcomes
Contextual influences
• Safeguarding issues
• Financial climate/cost
• Current guidelines
• Attitudes of clinicians
• Clinician training
• Clinician experience
• Fear of complaints
• Letter dictated in front of patient
• Patient background & clinical history
• Verbal communication with patient
• Letter templates available
• Competing priorities
• Feasibility
• Time constraints of writer
• Motivation
• Writer previous experience with patient letters
• Culture which promotes patient autonomy
is patient given choice about receiving copy of discharge communication?
yes
no
? Quality
Is summary written in plain English with minimal use of unexplained medical jargon or abbreviations?
Patient chooses to receive copy
Patient chooses not to receive copy
• No increase in patient knowledge • Letter errors remain • No patient questions
Patient does not receive copy
Patient copied into letter
Patient literacy level
Does patient discuss with friend/family member and/or healthcare provider?
Patient finds letter comprehensible
Patient feels more involved in care
Perception of 'shared-decision making'
• Increased patient understanding • Increase patient knowledge of condition • Patient corrects letter errors • Patient finds letter helpful • Patient does not contact healthcare provider with questions
• Improve quality &accuracy of letters • Empowered patients • Improve doctor-patient relationship & trust • Patient ownership • Patient would request letter again © • Improved communication • Increased patient involvement & autonomy • Patient acceptance of condition • Patient adheres to f/u plan • Reduce patient anxiety • ?reduce adverse events • Increase patient satisfaction
Patient does not find letter comprehensible
Patient feels confused/anxious
Patient feels letter is not important
• Harm to patient/patient distress • Low patient satisfaction • No increase in patient knowledge
Patient contacts healthcare provider (e.g. hospital or GP)
complaints
Patient discards letter/no further action
increased risk of confidentiality issues
Patient receives personalised discharge letter in plain English
Low level of literacy required to read letter
Patient literacy level
Patient understanding of letter
Patient values letter
• Increased patient understanding • Increase patient knowledge of condition • Patient corrects letter errors • Patient finds letter helpful • Patient does not contact healthcare provider with questions
Patient feels worried/confused
Patient contacts healthcare provider (e.g. hospital or GP)
No improvement in patient health literacy
No increase in patient recall
Does GP receive a copy? Is it useful/sufficient alone?

Figure 2

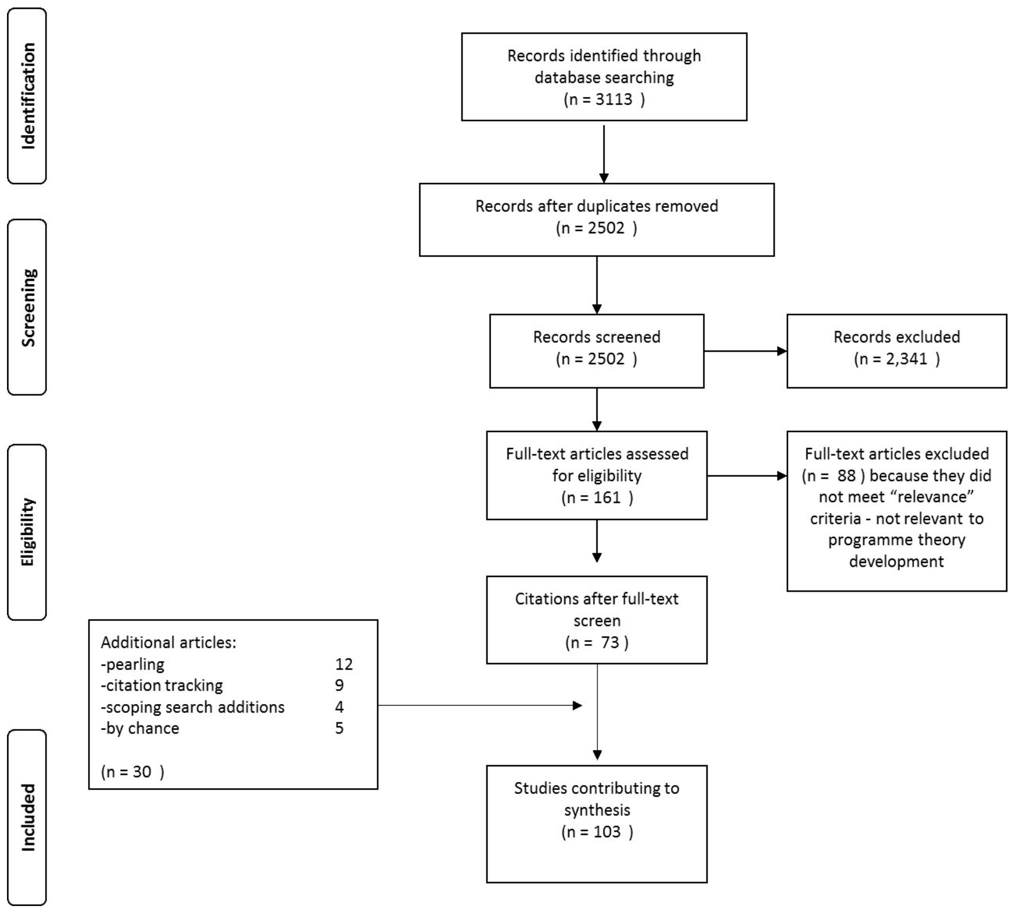
Identification
Screening
Eligibility
Included
Records identified through database searching (n = 3113 )
Records after duplicates removed (n = 2502 )
Records screened (n = 2502 )
Records excluded (n = 2,341 )
Full-text articles assessed for eligibility (n = 161 )
Full-text articles excluded (n = 88 ) because they did not meet "relevance" criteria - not relevant to programme theory development
Citations after full-text screen (n = 73 )
Additional articles:
-pearling 12
-citation tracking 9
-scoping search additions 4
-by chance 5
(n = 30 )
Studies contributing to synthesis (n = 103 )

Figure 3

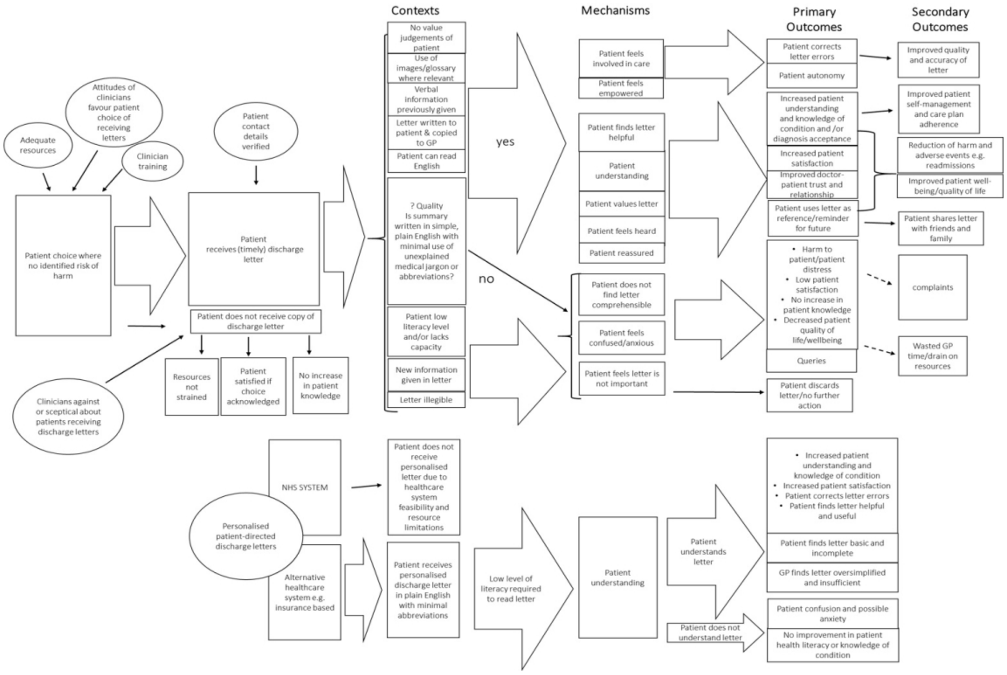
Contexts
Mechanisms
Primary Outcomes
Secondary Outcomes
Adequate resources
Attitudes of clinicians favour patient choice of receiving letters
Clinician training
Patient contact details verified
Patient choice where no identified risk of harm
Patient receives (timely) discharge letter
Patient does not receive copy of discharge letter
Resources not strained
Patient satisfied if choice acknowledged
No increase in patient knowledge
Clinicians against or sceptical about patients receiving discharge letters
No value judgements of patient
Use of images/glossary where relevant
Verbal information previously given
Letter written to patient & copied to GP
Patient can read English
? Quality Is summary written in simple, plain English with minimal use of unexplained medical jargon or abbreviations?
Patient low literacy level and/or lacks capacity
New information given in letter
Letter illegible
yes
no
Patient feels involved in care
Patient feels empowered
Patient finds letter helpful
Patient understanding
Patient values letter
Patient feels heard
Patient reassured
Patient does not find letter comprehensible
Patient feels confused/anxious
Patient feels letter is not important
Patient corrects letter errors
Patient autonomy
Increased patient understanding and knowledge of condition and /or diagnosis acceptance
Increased patient satisfaction
Improved doctor-patient trust and relationship
Patient uses letter as reference/reminder for future
• Harm to patient/patient distress
• Low patient satisfaction
• No increase in patient knowledge
• Decreased patient quality of life/wellbeing
Queries
Patient discards letter/no further action
Improved quality and accuracy of letter
Improved patient self-management and care plan adherence
Reduction of harm and adverse events e.g. readmissions
Improved patient well-being/quality of life
Patient shares letter with friends and family
complaints
Wasted GP time/drain on resources
NHS SYSTEM
Personalised patient-directed discharge letters
Alternative healthcare system e.g. insurance based
Patient does not receive personalised letter due to healthcare system feasibility and resource limitations
Patient receives personalised discharge letter in plain English with minimal abbreviations
Low level of literacy required to read letter
Patient understanding
Patient understands letter
Patient does not understand letter
• Increased patient understanding and knowledge of condition
• Increased patient satisfaction
• Patient corrects letter errors
• Patient finds letter helpful and useful
Patient finds letter basic and incomplete
GP finds letter oversimplified and insufficient
Patient confusion and possible anxiety
No improvement in patient health literacy or knowledge of condition

Figure 4

**Search Terms and Sources Searched**

| Source | Search terms |
|---|---|
| MEDLINE | 1. written[All Fields] AND ("patient discharge"[MeSH Terms]<br>2. ("patient"[All Fields] AND "discharge"[All Fields])<br>3. ("patient discharge"[All Fields] OR "discharge"[All Fields]) AND ("communication"[MeSH Terms])<br>4. ("communication"[All Fields]) AND ("patient discharge"[MeSH Major Topic]) AND ("patients"[MeSH Terms])<br>5. ("patients"[All Fields] OR "patient"[All Fields]) OR ("letter"[Publication Type] OR "correspondence as topic"[MeSH Terms])<br>6. ("correspondence"[All Fields]) AND ("patients"[MeSH Terms])<br>7. ("patients"[All Fields] OR "patient"[All Fields]) AND "patient discharge"[MeSH Major Topic]) AND ("communication"[MeSH Terms])<br>8. ("communication"[All Fields]) OR (receiving[All Fields]) AND ("letter"[Publication Type]) OR ("correspondence as topic"[MeSH Terms])<br>9. ("letters"[All Fields]) AND ("patients"[MeSH Terms] OR "patients"[All Fields] OR "patient"[All Fields]) AND ("patient discharge"[MeSH Major Topic])<br>10. ("patients"[MeSH Terms] OR "patients"[All Fields] OR "patient"[All Fields]) AND ((copies[All Fields]) AND "patient discharge"[MeSH Major Topic]))<br>11. ((("patient discharge"[MeSH Major Topic] OR "patient discharge"[MeSH Terms]) AND letter[Other Term]) AND ("patients"[MeSH Terms] OR "patients"[All Fields] OR "patient"[All Fields])<br>12. 1 OR 2 OR 3 OR 4 OR 5 OR 6 OR 7 OR 8 OR 9 OR 10 OR 11 |
| Web of Science | 1. Written patient discharge communication<br>2. Patients receiving letters<br>3. Patients receiving discharge letters<br>4. Patient copies of written information<br>5. 1 OR 2 OR 3 OR 4 |
| Department of Health | 1. Discharge communication<br>2. Patient letters |

For peer review only

| | |
|---|---|
| | 3. Patients receiving letters |
| Royal College of Physicians | 1. Discharge communication<br>2. Patient letters<br>3. Patients receiving letters<br>4. Patient copy<br>5. Patient copies<br>6. Patients receiving written information |

For peer review only

**Studies Found from Scoping Search**

| | Author | Year | Title | Document type |
|---|---|---|---|---|
| 1 | D N Wood, A Deshpande, M Wijewardena, and S S Gujral | 2006 | A Study of How Urology Out-Patients like to Receive Clinical Information | Published article |
| 2 | A Liapi, P J Robb, and A Akthar | 2006 | Copying clinic letters to patients: a survey of patient attitudes | Published article |
| 3 | S Baxter, K Farrell, C Brown, J Clarke, and H Davies | 2008 | Where have all the copy letters gone? A review of current practice in professional–patient correspondence | Published article |
| 4 | D D Pothier, P Nakivell, and C E J Hall | 2007 | What do Patients Think about being Copied into their GP Letters? | Published article |
| 5 | S L Todhunter, P J Clamp, S Gillett, and D D Pothier | 2010 | Readability of out-patient letters copied to patients: can patients understand what is written about them? | Published article |
| 6 | Royal College of Physicians | 2013 | Standards for the clinical structure and content of patient records | Guidelines |
| 7 | Royal College of Physicians | 2017 | Writing letters to patients – what's the big deal? | Short website entry |
| 8 | A J Choudhry, Y M K Baghdadi, A E Wagie, E B Habermann, S F Heller, D H Jenkins, D C Cullinane, M D Zielinski | 2016 | Readability of discharge summaries: with what level of information are we dismissing our patients? | Published article |
| 9 | M O'Reilly, M R Cahill, and I J Perry | 2006 | Writing to patients: a randomised controlled trial | Published article |
| 10 | Y Krishna, and B E Damato | 2005 | Patient attitudes to receiving copies of outpatient clinic letters from the ocular oncologist to the referring ophthalmologist | Published article |

For peer review only

| | | | | |
|---|---|---|---|---|
| | | | and GP | |
| 11 | B R O'Driscoll, J Koch, and C Paschalides | 2003 | Most patients want copies of letters from outpatient clinics and find them useful | BMJ letter |
| 12 | H Hadjistavropoulos, H Biem, D Sharpe, M Bourgault-Fagnou, and J Janzen | 2008 | Patient perceptions of hospital discharge: reliability and validity of a Patient Continuity of Care Questionnaire | Published article |
| 13 | M Thornber | 2009 | Copying referral letters | BJGP letter |
| 14 | Department of Health | 2000 | The NHS Plan | Report |
| 15 | P White, A Singleton, and R Jones | 2004 | Copying referral letters to patients: the views of patients, patient representatives and doctors | Published article |
| 16 | NHS England | 2016 | Standards for the communication of patient diagnostic test results on discharge from hospital | Guidance |
| 17 | R Lin, R Gallagher, M Spinaze, H Najoumian, C Dennis, R Clifton-Bligh, and G Tofler | 2014 | Effect of a patient-directed discharge letter on patient understanding of their hospitalisation | Published article |
| 18 | S Vaidyanathan, C A Glass, B M Soni, J Bingley, G Singh, J W H Watt, and P Sett | 2001 | Doctor ± Patient Communication: Do people with spinal cord injury wish to receive written information about their medical condition from the physicians after an outpatient visit or after a readmission in the spinal | Published article |

For peer review only

| | | | | |
|---|---|---|---|---|
| | | | unit? | |
| 19 | J Flacker, W Park, and A Sims, | 2007 | Hospital Discharge Information and Older Patients: Do They Get What They Need? | Published article |
| 20 | J S Albrecht, A L Gruber-Baldini, J M Hirshon, C H Brown, R Goldberg, J H Rosenberg, A C Comer, and J P Furuno, | 2014 | Hospital Discharge Instructions: Comprehension and Compliance Among Older Adults | Published article |
| 21 | B M Buurman, K J Verhaegh, M Smeulers, H Vermeulen, S E Geerlings, S Smorenburg, and S E de Rooij | 2016 | Improving handoff communication from hospital to home: the development, implementation and evaluation of a personalized patient discharge letter | Published article |
| 22 | Department of Health | 2003 | Copying letters to Patients: Good practice guidelines | Guidelines |
| 23 | J Main | 2008 | Copying in or copping out? | BMJ letter |
| 24 | C D Shee | 2008 | Try it and see | BMJ letter |
| 25 | B McKinstry | 2008 | Copying patients in is not as simple as it seems | BMJ letter |
| 26 | D Jelley, and T van Zwanenberg | 2000 | Copying general practitioner referral letters to patients: a study of patients' views | Published article |
| 27 | K Treacy, J S Elborn, J Rendall, and J M Bradley | 2008 | Copying letters to patients with cystic fibrosis (CF): Letter content and patient perceptions of benefit | Published article |

For peer review only

**Sources searched (step 2)**

| | Sources | Results |
|---|---|---|
| 1 | MEDLINE | 1596 |
| 2 | EMBASE | 558 |
| 3 | CINAHL | 100 |
| 4 | DARE | 2 |
| 5 | ASSIA | 47 |
| 6 | Web of Science | 205 |
| 7 | ZETOC | 29 |
| 8 | AMED | 26 |
| 9 | NHS Digital (HSCIC) | 0 |
| 10 | NHS Evidence (public domain only) | 244 |
| 11 | DH | 2 |
| 12 | NICE Guidelines | 0 |
| 13 | Cochrane database of systematic reviews | 21 |
| 14 | EPPI-CENTRE | 20 |
| 15 | SCOPUS | 38 |
| 16 | Google Scholar | 6 |
| 17 | OpenGrey | 3 |
| 18 | Greynet sources | 0 |
| 19 | ProQuest dissertations and theses | 210 |
| 20 | General Medical Council | 0 |
| 21 | Royal College of Physicians | 5 |
| 22 | Local Medical Committees (West Midlands) | 0 |
| 23 | Clinical Commissioning Groups (West Midlands) | 0 |
| 24 | SIGN | 0 |
| 25 | NHS Improvement | 1 |
| | TOTAL RESULTS | 3113 |

For peer review only

**Full List of Search Strategies for each source**

Search strategy for electronic databases (MEDLINE and AMED)

1. letter$
2. summaries$
3. Correspondence
4. patient copies$
5. patient letter
6. communication (MESH term if MEDLINE)
7. patient$ receiving
8. written information
9. discharge document$
10. patient-directed letter
11. personalised letter
12. personal letter
13. personalized letter
14. copy letter
15. 1 OR 2 OR 3 OR 4 OR 5 OR 6 OR 7 OR 8 OR 9 OR 10 OR 11 OR 12 OR 13 OR 14
16. Hospital discharge (MESH if MEDLINE) AND 15
17. Patient discharge (MESH if MEDLINE) AND 15
18. 16 OR 17
19. 18 and patients (MESH HEADING if MEDLINE)
20. Patients adj3 receiving adj3 letter*
21. Patients adj3 receiving adj3 discharge adj letter*
22. patient adj3 cop$ of written adj information
23. written adj3 patient adj discharge adj communication
24. secondary to primary adj care adj3 communication
25. hospital adj3 GP adj3 communication
26. writing adj3 to adj3 patients
27. 20 OR 21 OR 22 OR 23 OR 24 OR 25 OR 26
28. 27 OR 19
29. patient discharge letter
30. discharge communication
31. discharge letter
32. discharge summary
33. discharge summaries
34. 29 OR 30 OR 31 OR 32 OR 33 OR 34 OR 10 OR 11 OR 12 OR 13 OR 14
35. 34 OR 28
36. discharge correspondence
37. copy correspondence
38. doctor letter
39. copy letter
40. 36 OR 37 OR 38 OR 39
41. 40 OR 35

For peer review only

Search strategy for EMBASE and DARE (adapted due to high search results in EMBASE and simpler searching filtration system in DARE):

1. patient directed letter
2. personalised letter
3. personal letter
4. personalized letter
5. copy letter
6. Patients adj3 receiving adj3 letter*
7. Patients adj3 receiving adj3 discharge adj letter*
8. patient adj3 cop$ of written adj information
9. written adj3 patient adj discharge adj communication
10. secondary to primary adj care adj3 communication
11. hospital adj3 GP adj3 communication
12. writing adj3 to adj3 patients
13. patient discharge letter
14. discharge communication
15. discharge letter
16. patient discharge letter
17. discharge correspondence
18. copy correspondence
19. doctor letter
20. copy letter
21. 1 OR 2 OR 3 OR 4 OR 5 OR 6 OR 7 OR 8 OR 9 OR 10 OR 11 OR 12 OR 13 OR 14 OR 15 OR 16 OR 17 OR 18 OR 19 OR 20
22. 21 OR discharge summary (DARE ONLY)

Search strategy for CINAHL (adapted due to high search results):

1. patient discharge letter AND patient discharge from hospital (CINAHL MH "Hand Off (Patient Safety)")
2. personalised letter AND patient discharge from hospital (CINAHL MH "Hand Off (Patient Safety)")
3. copy letter AND patient discharge from hospital (CINAHL MH patient discharge summaries)
4. discharge communication AND patient discharge from hospital (CINAHL MH patient discharge summaries)
5. discharge correspondence AND patient discharge from hospital (CINAHL MH patient discharge summaries)
6. copy correspondence AND patient discharge from hospital (CINAHL MH patient discharge summaries)
7. doctor letter AND patient discharge from hospital (CINAHL MH patient discharge summaries)
8. copy letter
9. 1 OR 2 OR 3 OR 4 OR 5 OR 6 OR 7 OR 8

ASSIA and Web of Science and ZETOC and NHS evidence search strategy and NHS improvement and Cochrane database

1. (patient discharge letter) AND (discharge from hospital) AND "patient discharge" (*for NHS EVIDENCE & improvement & SCOPUS- AND written)

For peer review only

2. (personalised letter) AND (discharge from hospital) AND "patient discharge" (*for NHS EVIDENCE & improvement & SCOPUS - AND written)
3. (copy letter) AND (discharge from hospital) AND "patient discharge" (*for NHS EVIDENCE & improvement - AND written)
4. (doctor letter) AND (discharge from hospital) AND "patient discharge" (*for NHS EVIDENCE & improvement - AND written)
5. 1 OR 2 OR 3 OR 4
6. (FOR NHS EVIDENCE & improvement ONLY) copying hospital discharge letters to patients

ProQuest very high results (adapted terms)

1. personalised patient discharge letter AND "patient discharge" AND written AND patient information AND copy AND personalised AND copy letter AND doctor letter AND discharge communication copy OR letter "discharge from hospital"Google Scholar search: (many thousands of results when using above terms)

1. (personalised patient discharge letter) AND (discharge from hospital) AND "patient discharge" AND written AND patient information AND copy AND personalised AND copy letter AND doctor letter AND discharge communication AND written AND patient copy

Broad searches for X, Y, Z (websites and sources without indexing or electronic searching) e.g. Department of health

Used for: HSCIC and EPPI-CENTRE And Open Grey

The below search terms were entered into GMC yielding several hundred results. As results from GMC must be exported singularly these were screened on the webpage. The searches found no relevant results.

1. Discharge communication
2. Discharge
3. Patient discharge
4. Discharge letter
5. Discharge letters
6. Discharge summary
7. Discharge summaries
8. Patient letters
9. Patients receiving letters
10. Patients receiving letter
11. Patient copy
12. Copying patients
13. Patient copies
14. Patients receiving written information
15. Hospital discharge
16. Discharge correspondence
17. Discharge document
18. Discharge information
19. Patient discharge information

For peer review only

20. Copy letter
21. Doctor letter
22. Personal letter
23. Copy correspondence
24. Patient involvement
25. Patient access to records
26. Health informatics

*searches adapted in NHS digital due to huge amount of irrelevant results around admission statistics. No relevant results could be found.

For peer review only

**List of Included Texts (full)**


1. Ackermann S, Bingisser MB, Heierle A, Langewitz W, Hertwig R, Bingisser R. Discharge communication in the emergency department: physicians underestimate the time needed. Swiss Med Wkly. 2012;142:w13588.
2. Adams DC, Bristol JB, Poskitt KR. Surgical discharge summaries: improving the record. Ann R Coll Surg Engl. 1993;75(2):96-9.
3. Advancing effective communication, cultural competence, and patient- and family-centered care : a roadmap for hospitals. Joint Commission. 2014 http://www.jointcommission.org/assets/1/6/aroadmapforhospitalsfinalversion727.pdf.
4. Aguayo-Albasini JL, Garcia Garcia ML, Flores-Pastor B, Liron-Ruiz R. The importance of the discharge summary reports. Cir Esp. 2014;92(8):574-5.
5. Allan K, Ribbons B. Nurses combine IT and nursing skills to improve discharge communication. Aust Nurs J. 2006;14(1):30.
6. Antoniou A, Saunders M, Bourner R, Crouch L. would you like to see yours? The Bulletin of the Royal College of Surgeons of England. 2007;89(2):62-4.
7. Baumann W, Schussler L, Bertram M, Benser J, Kumpers S, Hermes-Moll K. Oncologists' letters for breast cancer patients. Oncology Research and Treatment. 2016;39:184-5.
8. Baxter S, Farrell K, Brown C, Clarke J, Davies H. Where have all the copy letters gone? A review of current practice in professional-patient correspondence. Patient Educ Couns. 2008;71(2):259-64.
9. Bench S, Day T, Griffiths P. Effectiveness of critical care discharge information in supporting early recovery from critical illness. Crit Care Nurse. 2013;33(3):41-52.
10. Bench SD, Heelas K, White C, Griffiths P. Providing critical care patients with a personalised discharge summary: a questionnaire survey and retrospective analysis exploring feasibility and effectiveness. Intensive Crit Care Nurs. 2014;30(2):69-76.
11. Bench S, Day T, Heelas K, Hopkins P, White C, Griffiths P. Evaluating the feasibility and effectiveness of a critical care discharge information pack for patients and their families: a pilot cluster randomised controlled trial. BMJ Open. 2015;5(11):e006852.
12. Boaden R, Harris C. Copying letters to patients—will it happen? : Oxford University Press; 2005.
13. Brockbank K. Copying patient letters - Making it work. Clinical Governance. 2005;10(3):231-40.
14. Brodie T, Lewis D. A survey of patient views on receiving vascular outpatient letters. European Journal of Vascular and Endovascular Surgery. 2010;39(1):5-10.
15. Brown CE, Roberts NJ, Partridge MR. Does the use of a glossary aid patient understanding of the letters sent to their general practitioner? Clinical medicine (London, England). 2007;7(5):457-60.
16. Buurman BM, Verhaegh KJ, Smeulers M, Vermeulen H, Geerlings SE, Smorenburg S, et al. Improving handoff communication from hospital to home: the development, implementation and evaluation of a personalized patient discharge letter. International Journal for Quality in Health Care. 2016;28(3):384-90.
17. Cannaby A-M. Improving the process of hospital discharge for medical patients [Ph.D.]. Ann Arbor: University of Leicester (United Kingdom); 2003.
18. Carol Lim KK, Chan SK, Chew EL, Anita Lim AF, Sararaks S, Ainul H, et al. Handoff communication - Let's do it right. Medical Journal of Malaysia. 2010;65:8.
19. Chantler C, Johnson J. Patients should receive copies of letters and summaries. BMJ : British Medical Journal. 2002;325(7360):388-.


For peer review only

20. Charlett SD, Bajaj Y, Kelly G. Writing to patients with the results of routine tests: A measure to improve access to outpatient clinics. Otorhinolaryngologist. 2009;2(3):73-4.
21. Choudhry AJ, Baghdadi YM, Wagie AE, Habermann EB, Heller SF, Jenkins DH, et al. Readability of discharge summaries: with what level of information are we dismissing our patients? Am J Surg. 2016;211(3):631-6
22. Damian D, Tattersall MH. Letters to patients: improving communication in cancer care. Lancet. 1991;338(8772):923-5.
23. Davies JM, Batuyong E, Lupichuk SM, Hilsden R, Eliasziw M, Easaw JC. Cohort study evaluating the impact of a discharge letter (DL) compared with usual care on adherence to surveillance following treatment for stage II/III colorectal cancer (CRC). Journal of Clinical Oncology Conference. 2012;30(4 SUPPL. 1).
24. Department of Health. The NHS Plan. 2000 http://webarchive.nationalarchives.gov.uk.
25. Department of Health. Copying letters to patients: good practice guidelines. 2003 http://webarchive.nationalarchives.gov.uk/
26. Discharge planning : best practice in transitions of care. The Queen's Nursing Institute. 2016 https://www.qni.org.uk/wpcontent/uploads/2016/09/discharge_planning_report_2015.pdf.
27. Dooher P, Syed A, Liu J, Chopra A, Bradpiece H, Jenkins S, et al. Copying letter to patients-distress or satisfaction? Eur J Cancer. 2012;48:S151.
28. Exploring patient participation in reducing health-care-related safety risks. 2013 http://www.euro.who.int/__data/assets/pdf_file/0010/185779/e96814.pdf.
29. Fayers T, Abdullah W, Walton V, Wilkins MR. Impact of written and photographic instruction sheets on patient behavior after cataract surgery. J Cataract Refract Surg. 2009;35(10):1739-43.
30. Fenton C, Al-Ani A, Trinh A, Srinivasan A, Marion K, Hebbard G. Impact of providing patients with copies of their medical correspondence: a randomised controlled study. Intern Med J. 2017;47(1):68-75.
31. Generic Standards Mar 2002. 2002 http://www.healthcareimprovementscotland.org/his/idoc.ashx?docid=ea8d290a-9504-4c21-a889-fc479d530a51&version=-1.
**32.** Guidelines on regional immediate discharge documentation for patients being discharged from secondary into primary care. Guidelines and Audit Implementation Network (GAIN). 2011 https://www.rqia.org.uk/RQIA/files/73/734a792f-f9d4-47f0-830f-31f9db51c82a.pdf**.**
33. Hahn-Goldberg S, Okrainec K, Damba C, Huynh T, Lau D, Maxwell J, et al. Implementing Patient-Oriented Discharge Summaries (PODS): A Multisite Pilot Across Early Adopter Hospitals. Healthc Q. 2016;19(1):42-8.
34. Hallowell N. Providing letters to patients. Patients find summary letters useful. Bmj. 1998;316(7147):1830.
35. Hayes KS. Literacy for health information of adult patients and caregivers in a rural emergency department. Clin Excell Nurse Pract. 2000;4(1):35-40.
36. HPOE: A Compendium of Implementation Guides 2011. 2011 http://www.hpoe.org/Reports-HPOE/hpoe_compendium_2011.pdf.
37. Hoek AE, De Ridder MA, Bayliss A, Patka P, Rood PP. Effective strategy for improving instructions for analgesic use in the emergency department. Eur J Emerg Med. 2013;20(3):210-3.
38. Holm H, Viktil KK. Patient satisfaction with drug reconciliation at discharge from hospital. Int J Clin Pharm. 2013;35 (6):1284.

For peer review only

39. Jelley D, van Zwanenberg T, Walker C. Copying letters to patients : Concerns of clinicians and patients need to be addressed first. BMJ : British Medical Journal. 2002;325(7376):1359-.
40. Knight AH, Mayon-White V. Writing to patients--annual review reports. Diabet Med. 1991;8(6):591.
41. Krishna Y, Damato BE. Patient attitudes to receiving copies of outpatient clinic letters from the ocular oncologist to the referring ophthalmologist and GP. Eye (London, England). 2005;19(11):1200-4.
42. Lepping P, Paravastu SC, Turner J, Billings P, Minchom P. Copying GP letters to patients: a comprehensive study across four different departments in a district general hospital. Health Inform J. 2010;16(1):58-62.
43. Liapi A, Robb PJ, Akthar A. Copying clinic letters to patients: a survey of patient attitudes. The Journal of Laryngology & Otology. 2006;121(6):588-91.
44. Lim CKK, Lim AAF, Ainul Nadziha MH, Roslinah A, Sararaks S, Chan SK, et al. Boleh balik! Medical Journal of Malaysia. 2010;65:69.
45. Lin R, Tofler G, Spinaze M, Dennis C, Clifton-Bligh R, Nojoumian H, et al. Patient-directed discharge letter (PADDLE)-a simple and brief intervention to improve patient knowledge and understanding at time of hospital discharge. Heart Lung and Circulation. 2012;21:S312.
46. Lin R, Gallagher R, Spinaze M, Najoumian H, Dennis C, Clifton-Bligh R, et al. Effect of a patient-directed discharge letter on patient understanding of their hospitalisation. Internal Medicine Journal. 2014;44(9):851-7.
47. Lin MJ, Tirosh AG, Landry A. Examining patient comprehension of emergency department discharge instructions: Who says they understand when they do not? Intern. 2015;10(8):993-1002.
48. Lorenzati B, Quaranta C, Perotto M, Tartaglino B, Lauria G. Discharge communication is an important underestimated problem in emergency department. Intern. 2016;11(1):157-8
49. Main J. Copying in or copping out? Bmj. 2008;337:a2688.
50. Making time in general practice : freeing GP capacity by reducing bureaucracy and avoidable consultations, managing the interface with hospitals and exploring new ways of working. NHS Alliance. 2015 http://www.nhsalliance.org/wp-content/uploads/2015/10/Making-Time-in-General-Practice-FULL-REPORT-01-10-15.pdf.
51. McConnell D, Butow P, Tattersall M. Audiotapes and letters to patients: the practice and views of oncologists, surgeons and general practitioners. British Journal of Cancer. 1999;79(11-12):1782.
52. McEniry B, Pillay I. How do patients in a rural setting respond to receiving a copy of their general practitioners letter? Ir Med J. 2008;101(3):84-5.
53. McKinstry B. Copying patients in is not as simple as it seems. Bmj. 2008;337:a2687.
54. Menon GJ, Dutton GN. Writing to our patients. Br J Ophthalmol. 1999;83(7):765.
55. Mrduljas Dujic N, Zitnik E, Pavelin L, Bacic D, Boljat M, Vrdoljak D, et al. Writing letters to patients as an educational tool for medical students. BMC Med Educ. 2013;13:114.
56. Mrduljas-Djujic N, Pavlicevic I, Marusic A, Marusic M. Students letters to patients as a part of education in family medicine. Acta Med. 2012;41(1):52-8.
57. NHS England . Standards for the communication of patient diagnostic test results on discharge from hospital. 2016. https://improvement.nhs.uk/uploads/documents/discharge-standards-march-16.pdf.
58. Nixon J, Courtney P. Copying clinic letters to patients. Rheumatology (Oxford). 2005;44(2):255-6.


For peer review only

59. O'Driscoll BR, Koch J, Paschalides C. Copying letters to patients: Most patients want copies of letters from outpatient clinics and find them useful. BMJ : British Medical Journal. 2003;327(7412):451-.
60. O'Reilly M, Cahill MR, Perry IJ. Writing to patients: a randomised controlled trial. Clin Med. 2006;6(2):178-82.
61. Paravastu S, Lepping P, Billings P. Copying clinic letters to surgical patients. The Bulletin of the Royal College of Surgeons of England. 2007;89(8):288-90.
62. Partridge MR, Roberts NJ. Writing to patients. Clin Med. 2006;6(3):319.
63. Perera KY, Ranasinghe P, Adikari AM, Balagobi B, Constantine GR, Jayasinghe S. Medium of language in discharge summaries: would the use of native language improve patients' knowledge of their illness and medications? J Health Commun. 2012;17(2):141-8
64. Perkins P, Jordan A, Prentice W, Regnard C. Copying letters to patients: a survey of patients and GPs views. Palliat Med. 2007;21(4):355-6.
65. Physicians RCo. Standards for the clinical structure and content of patient records. 2013 https://www.rcplondon.ac.uk/projects/outputs/standards-clinical-structure-and-content-patient-records.
66. Physicians RCo. Writing letters to patients – what's the big deal? 2017 https://www.rcplondon.ac.uk/news/writing-letters-patients-what-s-big-deal.
67. Pierce L. How to choose and develop written educational materials. Rehabilitation Nursing. 2010;35(3):99-105.
68. Pinder E, Jefferys S, Loeffler M. Patient Satisfaction: Receiving a copy of the GP letter following fracture or elective orthopaedic clinic. BMJ Quality Improvement Reports. 2013;2(2).
69. Polster D. Patient discharge information. Nursing. 2015;45(5):42-9.
70. Pothier DD, Nakivell P, Hall CE. What do patients think about being copied into their GP letters? Ann R Coll Surg Engl. 2007;89(7):718-21.
71. PRSB. Outpatient letter standard Consultation survey. 2017.
72. Rao M, Fogarty P. What did the doctor say? J Obstet Gynaecol. 2007;27(5):479-80.
73. Reddick B, Holland C. Reinforcing discharge education and planning. Nurs Manage. 2015;46(5):10-4.
74. Regalbuto R, Maurer MS, Chapel D, Mendez J, Shaffer JA. Joint Commission requirements for discharge instructions in patients with heart failure: is understanding important for preventing readmissions? J Card Fail. 2014;20(9):641-9.
75. Reilly MM. Let's set the record straight: preparing the discharge summary and the patient's instruction sheet. Nursing. 1979;9(1):56-61.
76. Reilly MO, Cahill M, Perry IJ. Writing to patients: 'putting the patient in the picture'. Ir Med J. 2005;98(2):58-60.
77. Roberts NJ, Partridge MR. How useful are post consultation letters to patients? BMC Medicine. 2006;4:2-.
78. Saidinejad M, Zorc J. Mobile and web-based education: delivering emergency department discharge and aftercare instructions. Pediatr Emerg Care. 2014;30(3):211-6.
79. Samuels-Kalow M, Rhodes K, Uspal J, Reyes Smith A, Hardy E, Mollen C. Unmet Needs at the Time of Emergency Department Discharge. Acad Emerg Med. 2016;23(3):279-87
80. Sandler DA, Heaton C, Garner ST, Mitchell JR. Patients' and general practitioners' satisfaction with information given on discharge from hospital: audit of a new information card. Bmj. 1989;299(6714):1511-3.
81. Sandler DA, Mitchell JR, Fellows A, Garner ST. Is an information booklet for patients leaving hospital helpful and useful? Bmj. 1989;298(6677):870-4.


For peer review only

82. Saunders NC, Georgalas C, Blaney SP, Dixon H, Topham JH. Does receiving a copy of correspondence improve patients' satisfaction with their out-patient consultation? J Laryngol Otol. 2003;117(2):126-9.
83. Sharma D, O'Brien S, Hardy K. Copying letters to patients: What patients think - A questionnaire survey. Clinician in Management. 2007;15(2):75-8.
84. Shee CD. Try it and see. Bmj. 2008;337:a2786.
85. Singh S, Budeda B, Housden P. Do patients want copies of their GP letters?–our experience with 7250 patients. Int J Clin Pract. 2007;61(8):1407-9.
86. Smith PEM. Letters to patients: sending the right message. BMJ : British Medical Journal. 2002;324(7338):685-.
87. Somov P, Madden T, Wong K, Hamm R. Security Concerns About Copying Clinical Letters to Patients. The Bulletin of the Royal College of Surgeons of England. 2013;95(1):33-4.
88. Sparkler 1 - Transitions of care in elderly patients. 2014 http://emahsn.org.uk/images/Section%208%20-%20Resource%20hub/Sparks%20and%20Sparklers/Sparkler_1_v6_SP1V1_FINAL_pdf_01-08-14.pdf.
89. Synthesis and conceptual analysis of the SDO programme's research on continuity of care. National Institute for Health Research (NIHR). 2010 http://www.netscc.ac.uk/hsdr/files/project/SDO_FR_08-1813-248_V01.pdf.
90. Tattersall R. Writing for and to patients. Diabet Med. 1990;7(10):917-9.
91. Taylor DM, Cameron PA. Discharge instructions for emergency department patients: What should we provide? Journal of Accident and Emergency Medicine. 2000;17(2):86-90.
92. Thornber M. Copy them in. Bmj. 2008;337.
93. Todhunter SL, Clamp PJ, Gillett S, Pothier DD. Readability of out-patient letters copied to patients: can patients understand what is written about them? J Laryngol Otol. 2010;124(3):324-7.
94. Tomkins CS, Braid JJ, Williams HC. Do dermatology outpatients value a copy of the letter sent to their general practitioner? In what way and at what cost? Clin Exp Dermatol. 2004;29(1):81-6.
95. Treacy K, Elborn JS, Rendall J, Bradley JM. Copying letters to patients with cystic fibrosis (CF): letter content and patient perceptions of benefit. Journal of cystic fibrosis : official journal of the European Cystic Fibrosis Society. 2008;7(6):511-4.
96. The Newcastle upon Tyne Hospitals NHS Foundation Trust Sharing Letters with Patients Policy. 2013.
97. Vaidya G. Copying letters to patients: Are we ready yet? Hospital Medicine. 2004;65(8):454-5.
98. Verhaegh KJ, Buurman BM, Veenboer GC, de Rooij SE, Geerlings SE. The implementation of a comprehensive discharge bundle to improve the discharge process: a quasi-experimental study. Neth J Med. 2014;72(6):318-25.
99. Walji M, Loeffelholz J, Valenza JA. A human-centered design of a dental discharge summary (DDS) for patients. AMIA Annu Symp Proc. 2007;Annual Symposium Proceedings/AMIA Symposium.:1146.
100. Warren J, Adnan M, Orr M. Iterative refinement of SemLink to enhance patient readability of discharge summaries. Stud Health Technol Inform. 2013;188:128-34.
101. Wimsett J, Harper A, Jones P. Review article: Components of a good quality discharge summary: a systematic review. Emerg Med Australas. 2014;26(5):430-8.

For peer review only

102. Zavala S, Shaffer C. Do patients understand discharge instructions? J Emerg Nurs. 2011;37(2):138-40.

103. Zeng-Treitler Q, Kim H, Hunter M. Improving patient comprehension and recall of discharge instructions by supplementing free texts with pictographs. AMIA Annu Symp Proc. 2008;Annual Symposium Proceedings/AMIA Symposium.:849-53.

For peer review only

| No. | Authors | Title |
|---|---|---|
| 49 | Reilly, M. M. | Let's set the record straight: preparing the discharge summary and the patient's instruction sheet |
| | Sandler, D. A.<br>Mitchell, J. R.<br>Fellows, A. | |
| 44 | Garner, S. T. | Is an information booklet for patients leaving hospital helpful and useful? |
| | Sandler, D. A.<br>Heaton, C.<br>Garner, S. T. | |
| 45 | Mitchell, J. R. | Patients' and general practitioners' satisfaction with information given on discharge from hospital: audit of a new in |
| 41 | Tattersall, R. | Writing to and for patients |

For peer review only

| | | |
|---|---|---|
| | Damian, D. | |
| 6 | Tattersall, M. H. N. | Letters to patients: improving communication in cancer care |
| | Knight, A. H. | |
| 69 | Mayon-White, V. | Writing to patients--annual review reports |
| | Adams, D. C. | |
| | Bristol, J. B. | |
| 93 | Poskitt, K. R. | Surgical discharge summaries: improving the record |
| 8 | Hallowell, N. | Providing letters to patients. Patients find summary letters useful |

For peer review only

| | | |
|---|---|---|
| 14 | McConnell, D<br>Butow, PN<br>Tattersall, MHN | Audiotapes and letters to patients: the practice and views of oncologists, surgeons and general practitioners |
| 62 | Menon, G. J.<br>Dutton, G. N. | Writing to our patients |
| 40 | Taylor, D. M.<br>Cameron, P. A. | Discharge instructions for emergency department patients: What should we provide? |
| 72 | Hayes, K. S. | Literacy for health information of adult patients and caregivers in a rural emergency department |

For peer review only

| | | |
|---|---|---|
| 5 | Chantler, Cyril<br>Johnson, James | Consultations do not have to be longer |
| 9 | Jelley, Diana<br>van Zwanenberg, Tim<br>Walker, Caron | Copying letters to patients : Concerns of clinicians and patients need to be addressed first |
| 26 | Smith, Philip E. M. | Letters to patients: sending the right message |
| 95 | Department of Health, | Generic Standards Mar 2002 |
| 17 | O'Driscoll, B. Ronan<br>Koch, Jan<br>Paschalides, Constantinos | Copying letters to patients: Most patients want copies of letters from outpatient clinics and find them useful |
| 43 | Saunders, N. C.<br>Georgalas, C.<br>Blaney, S. P.<br>Dixon, H.<br>Topham, J. H. | Does receiving a copy of correspondence improve patients' satisfaction with their out-patient consultation? |
| 77 | Department of Health, | The NHS Plan |

For peer review only

| | | |
|---|---|---|
| 78 | Department of Health, | Copying letters to patients: good practice guidelines |
| 83 | Cannaby, Ann-Marie | Improving the process of hospital discharge for medical patients |
| 38 | Vaidya, G. | Copying letters to patients: Are we ready yet? |

For peer review only

| | | |
|---|---|---|
| 39 | Tomkins, C. S.<br>Braid, J. J.<br>Williams, H. C. | Do dermatology outpatients value a copy of the letter sent to their general practitioner? In what way and at what cc |
| 2 | Boaden, Ruth<br>Harris, Claire | Copying letters to patients—will it happen? |
| 10 | Krishna, Y.<br>Damato, B. E. | Patient attitudes to receiving copies of outpatient clinic letters from the ocular oncologist to the referring ophthalm |
| 16 | Nixon, J<br>Courtney, P | Copying clinic letters to patients |

For peer review only

| | | |
|---|---|---|
| | Reilly, M. O. | |
| | Cahill, M. | |
| 48 | Perry, I. J. | Writing to patients: 'putting the patient in the picture' |
| 85 | Brockbank, K. | Copying patient letters - Making it work |
| | Liapi, A. | |
| | Robb, P. J. | |
| 12 | Akthar, A. | Copying clinic letters to patients: a survey of patient attitudes |
| | Roberts, Nicola J. | |
| 23 | Partridge, Martyn R. | How useful are post consultation letters to patients? |

For peer review only

| | | |
|---|---|---|
| | Partridge, M. R. | |
| 58 | Roberts, N. J. | Writing to patients |
| | O'Reilly, M. | |
| | Cahill, M. R. | |
| 59 | Perry, I. J. | Writing to patients: a randomised controlled trial |
| | Allan, K. | |
| 91 | Ribbons, B. | Nurses combine IT and nursing skills to improve discharge communication |
| | Antoniou, A | |
| | Saunders, MP | |
| | Bourner, R | |
| 1 | Crouch, L | Would you like to see yours? |
| | Brown, C. E. | |
| | Roberts, N. J. | |
| 4 | Partridge, M. R. | Does the use of a glossary aid patient understanding of the letters sent to their general practitioner? |

For peer review only

| | | |
|---|---|---|
| 18 | Paravastu, SCV<br>Lepping, P<br>Billings, PJ | Copying clinic letters to surgical patients |
| 19 | Perkins, Paul<br>Jordan, Alice<br>Prentice, Wendy<br>Regnard, Claud | Copying letters to patients: a survey of patients and GPs views |
| 25 | Singh, Sarvpreet<br>Budeda, B<br>Housden, P | Do patients want copies of their GP letters?–our experience with 7250 patients |
| 36 | Walji, M.<br>Loeffelholz, J.<br>Valenza, J. A. | A human-centered design of a dental discharge summary (DDS) for patients |

For peer review only

| | | |
|---|---|---|
| | Sharma, D.<br>O'Brien, S. | |
| 42 | Hardy, K. | Copying letters to patients: What patients think - A questionnaire survey |
| | Rao, M. | |
| 52 | Fogarty, P. | What did the doctor say? |
| | Pothier, D. D.<br>Nakivell, P. | |
| 53 | Hall, C. E. | What do patients think about being copied into their GP letters? |
| 13 | Main, J | Copying in or copping out? |
| | | Letters Copying letters to patients |
| 15 | McKinstry, B | Copying patients in is not as simple as it seems |

For peer review only

| | | |
|---|---|---|
| 24 | Shee, Charles D | try it and see |
| | Treacy, K. | |
| | Elborn, J. S. | |
| | Rendall, J. | |
| 30 | Bradley, J. M. | Copying letters to patients with cystic fibrosis (CF): letter content and patient perceptions of benefit |
| | Zeng-Treitler, Q. | |
| | Kim, H. | |
| 32 | Hunter, M. | Improving patient comprehension and recall of discharge instructions by supplementing free texts with pictographs |
| | McEniry, B. | |
| 63 | Pillay, I. | How do patients in a rural setting respond to receiving a copy of their general practitioners letter? |

For peer review only

| | | |
|---|---|---|
| | Baxter, S. | |
| | Farrell, K. | |
| | Brown, C. | |
| | Clarke, J. | |
| 89 | Davies, H. | Where have all the copy letters gone? A review of current practice in professional-patient correspondence |
| | Fayers, T. | |
| | Abdullah, W. | |
| | Walton, V. | |
| 74 | Wilkins, M. R. | Impact of written and photographic instruction sheets on patient behavior after cataract surgery |
| | Charlett, S. D. | |
| | Bajaj, Y. | |
| 82 | Kelly, G. | Writing to patients with the results of routine tests: A measure to improve access to outpatient clinics |

For peer review only

| | | |
|---|---|---|
| | Brodie, T | |
| 3 | Lewis, DR | A survey of patient views on receiving vascular outpatient letters |
| | Lepping, Peter | |
| | Paravastu, Sharath CV | |
| | Turner, Jim | |
| | Billings, Peter | |
| 11 | Minchom, Philip | Copying GP letters to patients: a comprehensive study across four different departments in a district general hospita |
| 28 | Thornber, M | copy them in |
| | Todhunter, S. L. | |
| | Clamp, P. J. | |
| | Gillett, S. | |
| 29 | Pothier, D. D. | Readability of out-patient letters copied to patients: can patients understand what is written about them? |
| 55 | Pierce, LI | How to choose and develop written educational materials |

For peer review only

| | | |
|---|---|---|
| | Lim, C. K. K. | |
| | Lim, A. A. F. | |
| | Ainul Nadziha, M. H. | |
| | Roslinah, A. | |
| | Sararaks, S. | |
| | Chan, S. K. | |
| | Chew, E. L. | |
| | Tan, L. S. | |
| | Low, L. L. | |
| | Azman, A. B. | |
| | Maimunah, A. H. | |
| 68 | Irdayu, H. | Boleh balik! |
| | Carol Lim, K. K. | |
| | Chan, S. K. | |
| | Chew, E. L. | |
| | Anita Lim, A. F. | |
| | Sararaks, S. | |
| | Ainul, H. | |
| | Roslinah, A. | |
| | Tan, L. S. | |
| | Low, L. L. | |
| | Azman, A. B. | |
| 80 | Maimunah, A. H. | handoff communication - lets do It right |
| 96 | NIHR | Synthesis and conceptual analysis of the SDO programme's research on continuity of care |
| | Zavala, S. | |
| 33 | Shaffer, C. | Do patients understand discharge instructions? |

For peer review only

| | | |
|---|---|---|
| 97 | GAIN | Guidelines on regional immediate discharge documentation for patients being discharged from secondary into prim |
| 103 | American Hospital Association | HPOE: A Compendium of Implementation Guides 2011 |
| 57 | Perera, K. Y.<br>Ranasinghe, P.<br>Adikari, A. M.<br>Balagobi, B.<br>Constantine, G. R.<br>Jayasinghe, S. | Medium of Language in Discharge Summaries: Would the Use of Native Language Improve Patients' Knowledge of Their Illness and Medications? |
| 60 | Mrduljas-Djujic, N.<br>Pavlicevic, I.<br>Marusic, A.<br>Marusic, M. | Students letters to patients as a part of education in family medicine |

For peer review only

| | | |
|---|---|---|
| | Lin, R.<br>Tofler, G.<br>Spinaze, M.<br>Dennis, C.<br>Clifton-Bligh, R.<br>Nojoumian, H. | |
| 65 | Gallagher, R. | Patient-directed discharge letter (PADDLE)-a simple and brief intervention to improve patient knowledge and under |
| | Dooher, P.<br>Syed, A.<br>Liu, J.<br>Chopra, A.<br>Bradpiece, H.<br>Jenkins, S. | |
| 76 | Patel, A. | Copying letter to patients-distress or satisfaction? |
| | Davies, J. M. Batuyong, E.<br>Lupichuk, S. M. Hilsden, R. | |
| 79 | Eliasziw, M. Easaw, J. C. | Cohort study evaluating the impact of a discharge letter (DL) compared with usual care on adherence to surveillance |
| | Pinder, Elizabeth<br>Jefferys, S | |
| 21 | Loeffler, Mark | Patient Satisfaction: Receiving a copy of the GP letter following fracture or elective orthopaedic clinic |

For peer review only

| | | |
|---|---|---|
| 27 | Somov, P<br>Madden, T<br>Wong, K<br>Hamm, R | Security Concerns About Copying Clinical Letters to Patients |
| 31 | The Newcastle upon Tyne Hospitals NHS Foundation Trust | The Newcastle upon Tyne Hospitals NHS Foundation Trust Sharing Letters with Patients Policy |
| 35 | Warren, J.<br>Adnan, M.<br>Orr, M. | Iterative refinement of SemLink to enhance patient readability of discharge summaries |
| 56 | Royal College of Physicians | Standards for the clinical structure and content of patient records |
| 61 | Mrduljas Dujic, N.Zitnik, E.Pavelin, L.Bacic,<br>Boljat, M.Vrdoljak, D.Pavlicevic,<br>I.Dvornik, A.Marusic, A.Marusic,<br>M. | Writing letters to patients as an educational tool for medical students |

For peer review only

| | | |
|---|---|---|
| 70 | Holm, H.<br>Viktil, K. K. | Patient satisfaction with drug reconciliation at discharge from hospital |
| 71 | Hoek, A. E.<br>De Ridder, M. A.<br>Bayliss, A.<br>Patka, P.<br>Rood, P. P. | Effective strategy for improving instructions for analgesic use in the emergency department |

For peer review only

| | | |
|---|---|---|
| | Bench, S.<br>Day, T. | |
| 88 | Griffiths, P. | Effectiveness of critical care discharge information in supporting early recovery from critical illness |
| 102 | World Health Organisation | Exploring patient participation in reducing health-care-related safety risks |
| | Wimsett, J.<br>Harper, A. | |
| 34 | Jones, P. | Review article: Components of a good quality discharge summary: a systematic review |

For peer review only

| | | |
|---|---|---|
| | Verhaegh, K. J<br>Buurman, B. .<br>Veenboer, G.C.de Rooij, S. E. | |
| 37 | Geerlings, S.E. | The implementation of a comprehensive discharge bundle to improve the discharge process: a quasi-experimental s |
| | Saidinejad, M. | |
| 47 | Zorc, J. | Mobile and web-based education: delivering emergency department discharge and aftercare instructions |
| | Regalbuto, R.<br>Maurer, M. S.<br>Chapel, D.<br>Mendez, J. | |
| 50 | Shaffer, J. A. | Joint Commission requirements for discharge instructions in patients with heart failure: is understanding important |

For peer review only

| | | |
|---|---|---|
| | Lin, R. | |
| | Gallagher, R. | |
| | Spinaze, M. | |
| | Najoumian, H. | |
| | Dennis, C. | |
| | Clifton-Bligh, R. | |
| 66 | Tofler, G. | Effect of a patient-directed discharge letter on patient understanding of their hospitalisation |
| | Bench, S. D. | |
| | Heelas, K. | |
| | White, C. | |
| 86 | Griffiths, P. | Providing critical care patients with a personalised discharge summary: a questionnaire survey and retrospective ana |
| | Aguayo-Albasini, J. L. | |
| | Garcia Garcia, M. L. | |
| | Flores-Pastor, B. | |
| 92 | Liron-Ruiz, R. | The importance of the discharge summary reports |
| 100 | The Joint Commission | Advancing effective communication, cultural competence, and patient- and family-centered care : a roadmap for ho |

For peer review only

| | | |
|---|---|---|
| 101 | Academic Health Science Network | Sparkler 1 - Transitions of care in elderly patients |
| | Reddick, B. | |
| 51 | Holland, C. | Reinforcing discharge education and planning |
| 54 | Polster, Debra | Patient discharge information |
| | Lin, M. J. | |
| | Tirosh, A. G. | |
| 67 | Landry, A. | Examining patient comprehension of emergency department discharge instructions: Who says they understand whe |

For peer review only

| | | |
|---|---|---|
| | Bench, S. | |
| | Day, T. | |
| | Heelas, K. | |
| | Hopkins, P. | |
| | White, C. | |
| 87 | Griffiths, P. | Evaluating the feasibility and effectiveness of a critical care discharge information pack for patients and their familie |
| 99 | Primary Care Foundation | Making time in general practice : freeing GP capacity by reducing bureaucracy and avoidable consultations, managir |

For peer review only

| | | |
|---|---|---|
| | Samuels-Kalow, M. | |
| | Rhodes, K. | |
| | Uspal, J. | |
| | Reyes Smith, A. | |
| | Hardy, E. | |
| 46 | Mollen, C. | Unmet Needs at the Time of Emergency Department Discharge |
| | Lorenzati, B. | |
| | Quaranta, C. | |
| | Perotto, M. | |
| | Tartaglino, B. | |
| 64 | Lauria, G. | Discharge communication is an important underestimated problem in emergency department |
| | Hahn-Goldberg, S. | |
| | Okrainec, K. | |
| | Damba, C. | |
| | Huynh, T. | |
| | Lau, D. | |
| | Maxwell, J. | |
| | McGuire, R. | |
| | Yang, L. | |
| 73 | Abrams, H. B. | Implementing Patient-Oriented Discharge Summaries (PODS): A Multisite Pilot Across Early Adopter Hospitals |
| 75 | NHS England | Standards for the communication of patient diagnostic test results on discharge from hospital |

For peer review only - http://bmjopen.bmj.com/site/about/guidelines.xhtml

| | | |
|---|---|---|
| | Choudhry, A. J.<br>Baghdadi, Y. M.<br>Wagie, A. E.<br>Habermann, E. B.<br>Heller, S. F.<br>Jenkins, D. H.<br>Cullinane, D. C. | |
| 81 | Zielinski, M. D.<br>Buurman, B. M.<br>Verhaegh, K. J.<br>Smeulers, M.<br>Vermeulen, H.<br>Geerlings, S. E.<br>Smorenburg, S. | Readability of discharge summaries: with what level of information are we dismissing our patients? |
| 84 | de Rooij, S. E.<br>Baumann, W.<br>Schussler, L.<br>Bertram, M.<br>Benser, J.<br>Kumpers, S. | Improving handoff communication from hospital to home: the development, implementation and evaluation of a pe |
| 90 | Hermes-Moll, K. | Oncologists' letters for breast cancer patients |

For peer review only

| | | |
|---|---|---|
| | Ackermann, S.<br>Heierle, A.<br>Bingisser, M. B.<br>Hertwig, R.<br>Padiyath, R.<br>Nickel, C. H.<br>Langewitz, W. | |
| 94 | Bingisser, R. | Discharge Communication in Patients Presenting to the Emergency Department With Chest Pain: Defining the Ideal |
| 98 | The Queen's Nursing Institute | Discharge planning : best practice in transitions of care |

For peer review only

| | | |
|---|---|---|
| | Fenton, Christine<br>Al-Ani, Aysha<br>Trinh, Andrew<br>Srinivasan, Ashish<br>Marion, Kaye | |
| 7 | Hebbard, G | Impact of providing patients with copies of their medical correspondence: a randomised controlled study |
| 20 | Royal College of Physicians | Writing letters to patients – what's the big deal? |
| | | Outpatient letter standard |
| 22 | PRSB | Consultation survey |

For peer review only

| Year | Country | Source type | Healthcare setting | Study design |
|---|---|---|---|---|
| 1979 | US | journal | Health insurance system | discussion |
| 1989 | England, UK | journal | nhs | questionnaire |
| 1989 | England, UK | journal | nhs | intervention+questionnaire |
| 1990 | England, UK | journal | nhs | discussion piece |

For peer review only

| | | | | |
|---|---|---|---|---|
| 1991 | Australia | Journal | public funded healthcare system | randomised study design |
| 1991 | England, UK | book | NHS | discussion |
| 1993 | England, UK | journal | nhs | Questionnaire study |
| 1998 | Scotland, UK | Journal letters | NHS | qualitative interview study |

For peer review only

| | | | | |
|---|---|---|---|---|
| 1999 | Australia | journal | public funded healthcare system | mixed methods study interview + quest |
| 1999 | Scotland, UK | Journal Commentary | NHS | discussion |
| 2000 | Australia | journal | public funded healthcare system | review |
| 2000 | US | journal | Health insurance system | descriptive study |

For peer review only

| | | | | |
|---|---|---|---|---|
| 2002 | England, UK | Journal letters | NHS | Discussion piece |
| 2002 | England, UK | Journal letters | NHS | postal survey |
| 2002 | England, UK | journal | NHS | Discussion piece |
| 2002 | England, UK | online | nhs | guidelines |
| 2003 | England, UK | Journal letters | NHS | Discussion piece |
| 2003 | England, UK | journal | nhs | questionnaire |
| 2003 | England, UK | DOH archive | NHS | guidelines |

For peer review only

| | | | | |
|---|---|---|---|---|
| 2003 | England, UK | DOH archive | NHS | guidelines |
| 2003 | England, UK | ProQuest | nhs | Thesis (PhD) |
| 2004 | England, UK | journal | nhs | discussion piece |

For peer review only

| | | | | |
|---|---|---|---|---|
| 2004 | England, UK | journal | nhs | service evaluation |
| 2005 | England, UK | Journal | National Health Service | no intervention. Discussion piece |
| 2005 | England, UK | journal | NHS | interviews |
| 2005 | England, UK | journal | NHS | questionnaire |

For peer review only

| | | | | |
|---|---|---|---|---|
| 2005 | Ireland | journal | public healthcare system | qualitative interview study |
| 2005 | England, UK | journal | nhs | questionnaire pilot study |
| 2006 | England, UK | journal | NHS | pilot/standard question format |
| 2006 | England, UK | journal | NHS | intervention |

For peer review only

| | | | | |
|---|---|---|---|---|
| 2006 | England, UK | journal | nhs | discussion |
| 2006 | England, UK | journal | nhs | a randomised controlled trial |
| 2006 | Australia | journal | public funded healthcare system | discussion |
| 2007 | England, UK | Journal | National Health Service | questionnaire |
| 2007 | England, UK | Journal | NHS | Questionnaire |

For peer review only

| | | | | |
|---|---|---|---|---|
| 2007 | England, UK | journal | NHS | questionnaire |
| 2007 | England, UK | journal | NHS | questionnaire |
| 2007 | England, UK | journal | NHS | pilot of intevention |
| 2007 | US | Symposium abstract | Health insurance system | abstract |

For peer review only

| | | | | |
|---|---|---|---|---|
| 2007 | England, UK | journal | nhs | questionnaire |
| 2007 | Ireland, UK | journal | NHS | questionnaire |
| 2007 | England, UK | journal | NHS | questionnaire |
| 2008 | England, UK | Journal letters | NHS | Discussion piece |
| 2008 | England, UK | Journal letters | NHS | Discussion piece |

For peer review only

| | | | | |
|---|---|---|---|---|
| 2008 | England, UK | Journal letters | NHS | Discussion piece |
| 2008 | England, UK | journal | NHS | Discussion piece+questionnaire |
| 2008 | US | journal | Health insurance system | pilot intervention |
| 2008 | Ireland | journal | public healthcare system | experimental study |

For peer review only

| | | | | |
|---|---|---|---|---|
| 2008 | England, UK | journal | nhs | review |
| 2009 | England, UK | journal | NHS | nonrandomized interventional clinical s |
| 2009 | England, UK | journal | nhs | experimental study |

For peer review only

| | | | | |
|---|---|---|---|---|
| 2010 | New Zealand | Journal | public funded healthcare system | Questionnaire on intervention |
| 2010 | England, UK | journal | NHS | postal survey |
| 2010 | England, UK | journal | NHS | Discussion piece |
| 2010 | England, UK | journal | NHS | Discussion piece |
| 2010 | US | journal | Health insurance system | discussion |

For peer review only

| | | | | |
|---|---|---|---|---|
| 2010 | Malaysia | conference listing | government's Ministry of Health | abstract for review |
| 2010 | Malaysia | conference listing | government's Ministry of Health | abstract |
| 2010 | England, UK | online | nhs | report |
| 2011 | US | journal | Health insurance system | observational qualitative design |

For peer review only

| | | | | |
|---|---|---|---|---|
| 2011 | Ireland, UK | online | nhs | guidelines |
| 2011 | us | online | insurance system | guide and report |
| 2012 | Sri Lanka | journal | public healthcare | prospective randomized design. |
| 2012 | Croatia | journal | health insurance plan | experimental study |

For peer review only

| | | | | |
|---|---|---|---|---|
| 2012 | Australia | journal | public funded healthcare system | pilot intervention |
| 2012 | England, UK | conference listing | NHS | abstract for questoinnaire study |
| 2012 | US | conference listing | Health insurance system | abstract for cohort study |
| 2013 | England, UK | journal | NHS | questionnaire |

For peer review only

| | | | | |
|---|---|---|---|---|
| 2013 | England, UK | journal | NHS | questionnaire |
| 2013 | England, UK | policy document | NHS | guidelines/policy |
| 2013 | New Zealand | journal | public funded healthcare system | protocol |
| 2013 | England, UK | online | NHS | guidelines |
| 2013 | Croatia | journal | health insurance plan | experimental study |

For peer review only

| | | | | |
|---|---|---|---|---|
| 2013 | Norway | Journal listing | public funded healthcare system | abstract for pre-post study |
| 2013 | The Netherland | Journal listing | Health insurance system | report |

For peer review only

| | | | | |
|---|---|---|---|---|
| 2013 | England, UK | journal | nhs | review |
| 2013 | Europe | online | N/A | recommendations |
| 2014 | New Zealand | journal | public funded healthcare system | systematic review |

For peer review only

| | | | | |
|---|---|---|---|---|
| 2014 | Netherlands | journal | Health insurance system | a quasi-experimental study |
| 2014 | US | journal | Health insurance system | discussion |
| 2014 | US | journal | Health insurance system | prospective cohort study |

For peer review only

| | | | | |
|---|---|---|---|---|
| 2014 | Australia | journal | public funded healthcare system | A prospective randomised controlled tr |
| 2014 | England, UK | journal | nhs | A questionnaire survey and retrospective analysis exploring feasibility and effectiveness |
| 2014 | Spain | Journal letters | public funded healthcare system | Letter to editor |
| 2014 | US | online | Health insurance system | guidelines |

For peer review only

| | | | | |
|---|---|---|---|---|
| 2014 | England, UK | online | nhs | review |
| 2015 | US | journal | Health insurance system | discussion |
| 2015 | US | journal | Health insurance system | guidelines |
| 2015 | US | journal | health insurance | survey |

For peer review only

| | | | | |
|---|---|---|---|---|
| 2015 | England, UK | journal | nhs | a pilot cluster randomised controlled trial |
| 2015 | England, UK | online | nhs | report |

For peer review only

| | | | | |
|---|---|---|---|---|
| 2016 | US | journal | Health insurance system | qualitative interview study |
| 2016 | Italy | journal | public healthcare system (national hea | discussion |
| 2016 | US | journal | Health insurance system | pilot |
| 2016 | England, UK | DOH archive | NHS | guidelines |

For peer review only

| | | | | |
|---|---|---|---|---|
| 2016 | US | journal | Health insurance system | cohort study |
| 2016 | The Netherland | journal | Health insurance system | before-after evaluation study |
| 2016 | Germany | conference listing | Health insurance system | abstract for survey design |

For peer review only

| | | | | |
|---|---|---|---|---|
| 2016 | Switzerland | journal | private health insurance | single-center cross-sectional study |
| 2016 | England, UK | online | nhs | best practice guidelines |

For peer review only

| | | | | |
|---|---|---|---|---|
| 2017 | Australia | Journal | public funded healthcare system | a randomised controlled study |
| 2017 | England, UK | online post | NHS | Discussion piece |
| 2017 | England, UK | online | NHS | guidelines |

For peer review only

| Topic focus | Study aim | No. of subjects |
|---|---|---|
| summary instructions | to encourage better recording of clinical stays | n/a |
| patient views on helpfulness of discharge instructi | Objective-To determine whether a booklet given to patients being discharged from hospital giving details of their admission and treatment increased their knowledge and recall when reviewed in outpatient clinics.' | 131 |
| information card usefulness | To determine the attitudes of patients discharged from hospital and their general practitioners to a new information card giving details about admission, diagnosis, and treatment and to assess the completeness of the information on the card.' | 258 |
| writing to patients | to discuss advanatges and disadvanatges of writing to patients | N/A |

For peer review only

| | | |
|---|---|---|
| letters to patients | to assess the role of personal patient letter | 48 |
| writing to patients | to discuss involvement of patients in hospital to GP communica | none specified |
| Improving discharge summaries | "assess the quality of previous discharge summaries and the new computergenerated discharge summaries," | 118 |
| use of written summaries | to explore patients' attitudes about and their use of written summaries of their genetic consultations for hereditary breast and ovarian cancer | 40 |

For peer review only

| | | | |
|---|---|---|---|
| To gather current practices and views of different ( | "To identify the proportion of oncologists, surgeons and GPs in favour of offering cancer patients an audiotape of the consultation and/or a summary letter." | | 319 |
| writing to patients | To discuss the benefits of writing to patients | n/a | |
| discharge instructions | to examine 'the purpose, advantages, and disadvantages of three commonly used types of discharge instruction' | N/A | |
| health literacy of patients | to look at literacy for health information | | 195 |

For peer review only

| | | | |
|---|---|---|---|
| Patient copies of letters - focus on benefits | N/A | N/A | |
| Copies of clinical correspondence to patients | to discuss concerns of clinicians | | 229 |
| copies of letters | To discuss copying patient letters and guidelines for letters | N/A | |
| hospital standards | to outline NHS standards | N/A | |
| Copies of letters | To discuss the benefits of copy patient letters | N/A | |
| patients views on copy letters | to see whether patients receiving a copy letter improved satisfaction with their consultation | | 117 |
| NHS plans | to provide guidelines and standards of patient care | n/a | |

For peer review only

| | | | |
|---|---|---|---|
| copying letters to patients | to provide guidelines and standards of patient care | n/a | |
| patient education | "To explore the barriers to the process of discharge and to investigate possible interventions to overcome them." | | 50 |
| copies of letters | to discuss the current situation with copying letters to patients | N/A | |

For peer review only

| | | | |
|---|---|---|---|
| whether patients value copy of letter | to explore the value patients attach to receiving such a letter and to estimate the cost of this practice.' | | 49 |
| Copying all letters to patients | N/A | N/A | |
| Copies of letters | To understand patient attitudes to copies | | 52 |
| Copies of letters | To gather opinions of clinicians and patients on copy letter | | 117 |

For peer review only

| | | |
|---|---|---|
| summary letters for patients | To explore consultant, general practitioner and patient attitudes towards the proposal that following an outpatient consultation, consultants should consider communicating directly with patients in the form of a summary letter, with a copy to the referring general practitioner or other professionals as appropriate' | 48 |
| patient copy letters | "to describe how one acute Trust has used basic change management principles to implement the initiative." | 76 |
| Copies of letters | to examine attitudes of ENT patients on copies of letters | 200 |
| letters to patients | To gather opinions of patients on receiving letter | 84 |

For peer review only

| | | |
|---|---|---|
| patient letters | to discuss patient copies and patient letters | n/a |
| patient letters | to look at patient recall with patient letters | 150 |
| electronic discharge letters | to improve discharge communicaton through use of electonic letter invervention | None specified |
| Copies of GP letters | to assess patients' responses to the proposed legislation (copies of GP correspondence) | 500 |
| Copying GP letters to patients with glossary | to evaluate a lung disease glossary to enhance patient understanding of terms used within the letter sent to their GP. | 93 |

For peer review only

| | | |
|---|---|---|
| copies and personal patient letter | To gather opinions of clinicians and patients on copy letter | 175 |
| Copies of letters | To gather opinions of clinicians and patients on copy letter | 21 |
| copies of letters | " to determine how many patients attending Trauma and Orthopaedic clinics actively wished to receive copies of these letters" | 7250 |
| patient discharge summary | to generate a paper-based discharge summary prototype.' N/A | |

For peer review only

| | | | |
|---|---|---|---|
| patients views on copy letters | to evaluate patients views on receiving copy letters | | 220 |
| patient satisfaction with discharge letters | to assess patient satisfaction with discharge letter copies | | 78 |
| patient views on copies of letters | to find out what patients think of being copied into their letters | | 500 |
| Copies of letters | Is copy letters a new target as a cop out for good communication | N/A | |
| Copies of letters | To discuss the drawbacks on patient copies of letters | N/A | |

For peer review only

| | | | |
|---|---|---|---|
| copies of letters | To discuss the benefits of copy patient letters | N/A | |
| copies of letters | to assess readability of patient letter | N/A | |
| use of pictures with instructions to improve unders | to test use of pictures with discharge instructions for patients | | 13 |
| patients receiving letters | To assess how patients feel about receiving a copy of their lette | | 130 |

For peer review only

| | | | |
|---|---|---|---|
| patients receiving letters | "This article reviews the literature in relation to patients receiving copies of health professional correspondence. It examines progress in<br>adopting the practice 3 years on from its introduction as policy in the UK, and considers potential benefits and obstacles to implementation." | N/A | |
| use of written discharge instructions plus photogra | "To evaluate the extent to which patients unnecessarily restrict activities of daily living<br>after routine cataract surgery and to test interventions to increase activity." | | 150 |
| test results after discharge | to assess writing to patients with test results after discharge | | 100 |

For peer review only

| | | | |
|---|---|---|---|
| Copying letters | to find out how valuable patients find copies of letters | | 68 |
| Copies of letters | to determine whether patients are satisfied with their current communication or prefer alternative modes, overall and between different specialty clinics | | 483 |
| copies of letters | To discuss copying patient letters and guidelines for letters | N/A | |
| copies of letters | to assess readability of patient letter | N/A | |
| education materials | to promote healthy behaviours through patient education | n/a | |

For peer review only

| | | |
|---|---|---|
| discharge communication | "identifying strategies that are effective in improving discharge | none specified |
| discharge information | to improve discharge communication | n/a |
| Service delivery | "to identify what influences the experience and delivery of continuity of care both overall and for different patient groups" | 193 |
| patient understanding | to assess patient understanding of discharge instructions | 50 |

For peer review only

| | | |
|---|---|---|
| discharge documents | to report on discharge standards | N/A |
| medical activities and care | to identify ways of improving care | N/A |
| patient information | to assess whether patients prefer a copy of discharge summary | 130 |
| patient letters | to look at students writing letters to patients | none specified |

For peer review only

| | | |
|---|---|---|
| patient letters | "To determine the effect of a patient-directed discharge letter (PADDLE) on patient knowledge and understanding of their diagnosis and treatment plan." | 62 |
| copying letters to patients | to look at patients response to receiving copy letters | 217 |
| impact of discharge letter to patients | To look at use of discharge letter | 332 |
| letters to patients | To gather opinions of patients on receiving letter | 124 |

For peer review only

| | | | |
|---|---|---|---|
| copies of letters | " to evaluate this service as well as explore patient preferences and any issues that may affect the security of confidential information." | | 94 |
| copies of letters | to outline policy for sharing letters with patients | N/A | |
| readibility | "to enhance the semantic annotation and dynamic hyperlinking algorithms to link topic-specific web pages for difficult terms found occurring in Discharge Summary text." | N/A | |
| patient records | to provide guidance for appropriate and good standards of clini | n/a | |
| patient letters | to look at students writing letters to patients | none specified | |

| | | |
|---|---|---|
| patient information | "to investigate whether a new template for drug information (based on the IMM model) could increase readability and usefulness for the patients, compared with the standard discharge letter." | 34 |
| improving discharge instructions | "to evaluate a strategy for improving our patients' recall of their instructions for taking pain medication." | 50 |

For peer review only

| | | | |
|---|---|---|---|
| discharge information | to look at effectiveness of discharge information through review | | 406 |
| patient involvement | to explore relationships between patient rights and safety | N/A | |
| content | to assess content of good quality discharge summary | N/A | |

For peer review only

| | | | |
|---|---|---|---|
| discharge process | to evaluate the discharge bundle | | 428 |
| to look at different ways of delivering discharge ins | to look at different ways of delivering discharge instructions | none specified | |
| joint commission standards | to test patient understanding of discharge instructions in relation to 30 day readmission rates | | 145 |

For peer review only

| | | | |
|---|---|---|---|
| patient letters | to determine "patient understanding of the reasons for hospitalisation, in-hospital tests, treatments and post–discharge recommendations, and whether a brief patient-directed discharge letter (PADDLE) delivered during a brief discussion prior to discharge would improve understanding." | | 67 |
| personalised discharge patient letters | to explore feasibility of providing patients with personalised discharge letters | | 232 |
| Patients receiving letters and letter quality | to discuss patient letters and discharge process from surgery | None specified | |
| hospital communication | to provide guidance on hospital activities | N/A | |

For peer review only

| | | | |
|---|---|---|---|
| care transitions | to provide guidance on hospital activities | N/A | |
| discharge planning | to discuss ways of improving discharge planning and informatio | n/a | |
| discharge education | to provide guidance on patient discharge | n/a | |
| patient understanding | to examine patient comprehension of discharge instructions | | 75 |

For peer review only

| | | |
|---|---|---|
| discharge packs | "To evaluate the feasibility and effectiveness of an information pack, based on selfregulation theory, designed to support patients and their families immediately before, during and after discharge from an intensive care unit (ICU)." | 158 |
| General Practice | to report on managing the interface with hospitals | N/A |

For peer review only

| | | | |
|---|---|---|---|
| to look at unmet needs at discharge | to characterize the variation in needs and preferences regarding the ED discharge process by health literacy and identify novel ideas for process improvement from parents and patients.' | | 51 |
| discharge communication | To discuss the importance and appropriate practice of discharg | | 50 |
| test of patient orientated discharge summary | to look at use of patient summary | >200 | |
| discharge process | to provide guidance on discharge practice in the NHS | n/a | |

For peer review only

| | | |
|---|---|---|
| readability of discharge summaries | "to assess the readability of patient hospital dismissal summaries and compare this to the patient's educational level." | 497 |
| patient letters | "To develop, implement and evaluate a personalized patient discharge letter (PPDL) to improve the quality of handoff communication from hospital to home." | 141 |
| patients receiving letters | looking at patients receiving copies of letters | 9 |

For peer review only

| | | |
|---|---|---|
| discharge information for patients | "investigating and improving physician–patient communication in an ED discharge setting by identifying the information that needs to be covered in this interaction" | 98 |
| discharge planning | to report on discharge standards | 608 |

For peer review only

| | | | |
|---|---|---|---|
| Copies of clinical correspondence to patients | To determine whether providing clinic correspondence and endoscopy reports to patients leads to improved understanding, satisfaction or anxiety. | | 70 |
| Copies of letters | To discuss the benefits of copy patient letters | N/A | |
| letters to patients | To discuss copying patient letters and guidelines for letters | N/A | 16383 |

For peer review only

| Type of intervention | Form of discharge communication | Specialty |
| --- | --- | --- |
| n/a | discharge summary & instructions | none specified |
| discharge instructions | discharge instructions | cardiology |
| discharge information card | discharge instructions | general medical ward |
| N/A | patient letter | diabetes clinic |

For peer review only

| | | |
|---|---|---|
| Patient sent personal letter | personal patient letter | Oncology |
| none specified | none specified | diabetes clinic |
| N/A | N/A | None specified |
| patient given summary | summary | Oncology |

For peer review only

| | | |
|---|---|---|
| N/A | Copies & personal summaries | Oncology, surgery & general practice |
| patient letter | patient letter | Eye care |
| N/A | discharge instructions | Emergency Department |
| discharge instructions | discharge instructions | Emergency Department |

| | | |
|---|---|---|
| N/A | Copy | N/A |
| patient sent copy | Copy | none specified |
| N/A | Copy | none specified |
| N/A | discharge summary | none specified |
| N/A | Copy | None specified |
| copy letter | copy of discharge letter | ENT |
| n/a | copy of discharge letter | n/a |

For peer review only

| | | |
|---|---|---|
| n/a | copy of discharge letter | n/a |
| booklet and facilitated presentat | discharge booklet | none specified |
| N/A | discharge letter | none specified |

For peer review only

| | | |
|---|---|---|
| telephone call | copy of discharge letter | Dermatology |
| N/A | Copies of all letters | all |
| patient sent copy | Copy | ocular oncology |
| N/A | Copy | rheumatology |

For peer review

| | | |
|---|---|---|
| discharge summary | discharge summary | none specified |
| copy of letter | copy of letter | none specified |
| patient offered copy | Copy | ENT |
| copy of letter | Copy | cardiorespiratory |

For peer review only

| | | |
|---|---|---|
| n/a | copy & patient letter | none specified |
| patient letter | patient letter | haematology |
| copy | copy | None specified |
| N/A | Copies of GP correspondence | colorectal surgery |
| Copy of letter + glossary | copy + glossary | Respiratory |

For peer review only

| | | |
|---|---|---|
| N/A | copy & personal letter | mixed |
| N/A | Copy | palliative care |
| copy of letter | Copy | Trauma and Orthopaedic |
| N/A | discharge summary | dental |

For peer review only

| | | |
|---|---|---|
| copy letter | copy of discharge letter | diabetes clinic |
| copy | copy of discharge letter | gynaecology |
| copy | copy of discharge letter | ENT |
| N/A | Copy | N/A |
| N/A | Copy | None specified |

For peer review only

| | | |
|---|---|---|
| N/A | Copy | none specified |
| N/A | Copy | none specified |
| instructions + pictures | instructions + pictures | ?Cardiology application but volunteers healthy |
| copy | copy | Geriatric and general internal medicine |

For peer review only

| | | |
|---|---|---|
| N/A | copy | N/A |
| discharge instructions/photogra | discharge instructions | Eye care |
| patient letter | patient letter | otolarngology |

For peer review only

| | | |
|---|---|---|
| Sent copy of letter | Copy | Vascular |
| patient sent copy | Copy | paediatrics, psychiatry, medicine and sur |
| N/A | Copy | none specified |
| N/A | Copy | none specified |
| n/a | n/a | none specified |

For peer review only

| | | |
|---|---|---|
| none specified | none specified | none specified |
| n/a | n/a | n/a |
| N/A | discharge information | none specified |
| telephone call | instructions | Emergency Department |

For peer review only

| | | |
|---|---|---|
| N/A | discharge information | none specified |
| N/A | patient information | none specified |
| copy of discharge summary in na | copy (language variation) | none specified |
| patient letter | patient letter | none specified |

For peer review only

| | | |
|---|---|---|
| patient directed letter | patient letter | cardiology 47, respiratory 10, endocrine & general medicine 5 |
| copy | copy of discharge letter | Oncology |
| copy of discharge letter | copy of discharge letter | Oncology |
| N/A | Copy | fracture clinic |

For peer review only

| | | |
|---|---|---|
| N/A | Copy | urology |
| N/A | Copy | none specified |
| N/A | discharge summary | none specified |
| n/a | n/a | none specified |
| patient letter | patient letter | none specified |

For peer review only

| | | |
|---|---|---|
| new discharge template | new discharge template | none specified |
| discharge instructions | discharge instructions | Emergency Department |

For peer review only

| | | |
|---|---|---|
| mixed | discharge information | critical illness/general ward |
| N/A | patient information | none specified |
| N/A | discharge summary | Emergency Department |

For peer review only

| | | |
|---|---|---|
| discharge bundle | the personalised patient discharge letter | medical |
| technology | discharge instructions | none specified |
| discharge instructions | discharge instructions | heart failure patients (cardiology) |

For peer review only

| | | |
|---|---|---|
| patient directed letter | patient letter | cardiology, respiratory and endocrinology wards |
| personalised patient discharge le | personalised patient discharge letter | medical, surgical and traumapatient popu |
| N/A | copy | Surgery |
| N/A | discharge information | none specified |

For peer review only

| | | |
|---|---|---|
| N/A | discharge information | Geriatric |
| n/a | discharge instructions | none specified |
| n/a | n/a | none specified |
| discharge instructions | discharge instructions | Emergency Department |

For peer review only

For peer review only

| | | |
|---|---|---|
| patient discharge pack | patient discharge pack | Intensive Care Unit |
| N/A | discharge information | none specified |

| | | |
|---|---|---|
| n/a | discharge process | Emergency Department |
| n/a | discharge instructions | Internal Emergency Medicine |
| patient directed letter | patient directed letter | mix of specialities |
| n/a | n/a | n/a |

For peer review only

| | | |
|---|---|---|
| discharge summary | discharge summary | trauma |
| personalised patient discharge le | personalised patient discharge letter | medical ward |
| copy | copy | Oncology (breast cancer) |

For peer review only

| | | |
|---|---|---|
| discharge information | discharge information | Emergency Department |
| N/A | discharge information | none specified |

For peer review only

| | | |
|---|---|---|
| patient sent copy | Copy | gastroenterology |
| N/A | Copy | N/A |
| N/A | Copy | none specified |

For peer review only

| Restrictions/exclusions | Participant mix | Professional mix |
|---|---|---|
| none specified | none specified | none specified |
| none specified | none specified | none specified |
| none specified | none specified | 3 consultants |
| none specified | none specified | none specified |

For peer review only

| | | |
|---|---|---|
| Patients who did not speak English (14) or who were considered too ill or disabled to be interviewed (7) were also excluded. | Oncology outpatients eligible | consultants |
| none specified | diabetic patients | none specified |
| None specified | None specified | GPs |
| N/A | ALL | ALL |

For peer review only

| | | |
|---|---|---|
| None specified | 156 Onc,55 surg, 108 GPs | GPs, surgeons, oncologists |
| none specified | outpatients | none specified |
| none specified | none specified | none specified |
| none specified | none specified | none specified |

For peer review only

| | | |
|---|---|---|
| N/A | N/A | N/A |
| N/A | none specified | none specified |
| none specified | none specified | none specified |
| N/A | N/A | N/A |
| None specified | None specified | None specified |
| none specified | none specified | none specified |
| n/a | n/a | n/a |

For peer review only

| | | |
|---|---|---|
| n/a | n/a | n/a |
| "Speciality specific discharge, e.g. Neonatal or psychiatric<br>• Articles written in a language other than English<br>• Studies of pharmaceutical interventions<br>• Articles on children/adolescent discharge<br>• Discharge from outpatients, day cases or A&E (in patient stay required)" | inpatients | hospital and community staff |
| none specified | none specified | none specified |

For peer review only

| | | |
|---|---|---|
| none specified | none specified | none specified |
| N/A | ALL | ALL |
| N/A | 30 females and 22 males, aged between 24 and 87 years (median 61 years | consultants |
| None specified | patients | mix of doctors and nurses |

For peer review only

| | | |
|---|---|---|
| none specified | 16 patients | 20 consultants, 12 GPs |
| none specified | none specified | hospital staff |
| None specified | 100 adults, 100 children/parents | clinic doctors |
| none specified | none specified | none specified |

For peer review only

| | | |
|---|---|---|
| none specified | none specified | none specified |
| none specified | outpatients | consultants |
| None specified | None specified | nurses |
| Patients attending either of the two weekly clinics under the supervision of one consultant surgeon (MP Saunders) were recruited. | outpatients | 1 consultant surgeon |
| None specified | ALL | Non-clinical staff, consultants |

For peer review only

| | | |
|---|---|---|
| None specified | patients | doctors and nurses |
| None specified | patients | GPs |
| none specified | outpatients | none specified |
| none specified | none specified | none specified |

For peer review only

| | | |
|---|---|---|
| none specified | none specified | none specified |
| none specified | gynaecology surgery patients | 1 consultant surgeon |
| INCLUSION CRITERIA<br>1. Patients who had been seen in the RUH ENT out-patient<br>department during the preceding 2 years during which<br>copy letters were being offered routinely.<br>2. Adults or parents of children who were patients.<br>EXCLUSION CRITERIA<br>1. New patients.<br>2. Children.<br>Where patients were unable to complete the questionnaire<br>by themselves, assistance was offered. | outpatients | consultants |
| None specified | None specified | None specified |
| None specified | None specified | None specified |

For peer review only

| | | |
|---|---|---|
| none specified | none specified | none specified |
| none specified | none specified | none specified |
| none specified | healthy volunteers | none specified |
| letters withheld at doctors disecretion e.g. serious diagnosis. Letters written by clinician not taking part excluded | elderly outpatients | hospital clinicians |

For peer review only

| | | |
|---|---|---|
| None specified | none specified | none specified |
| none specified | none specified | none specified |
| discharged patients | outpatients | specialist registrars |

For peer review only

| | | |
|---|---|---|
| all patients (both new and follow up) attending a vascular outpatient clinic between the dates of 20 August 2008 and 19 November 2008. | Vascular outpatients. 63% male. Median age 73 | consultants |
| Patients attending four specialty outpatient clinics – paediatrics, psychiatry, medicine and surgery | Any | none specified |
| none specified | none specified | none specified |
| none specified | none specified | none specified |
| none specified | none specified | none specified |

For peer review only

| | | |
|---|---|---|
| none specified | none specified | none specified |
| n/a | n/a | n/a |
| N/A | N/A | N/A |
| none specified | mixed patients | none specified |

For peer review only

 

| | | |
|---|---|---|
| N/A | N/A | N/A |
| N/A | N/A | N/A |
| none specified | newly diagnosed patients with noncommunicable chronic diseases | none specified |
| none specified | none specified | trainee doctors/medical students |

For peer review only

| | | |
|---|---|---|
| none specified | inpatients | none specified |
| none specified | breast unit patients | none specified |
| n/a | n/a | n/a |
| adults | all patients | none specified |

For peer review only

| | | |
|---|---|---|
| none specified | none specified | consultants |
| none specified | none specified | none specified |
| none specified | none specified | none specified |
| none specified | none specified | none specified |
| none specified | none specified | 3 family medicine experts & medical students |

For peer review only

| | | |
|---|---|---|
| none specified | inpatients | none specified |
| "over 18 years of age and discharged on analgesics with verbal instructions only (e.g. paracetamol, NSAIDs and/or tramadol, as reported in patient's file) were eligible to take part. Exclusion criteria were: patients unable to read and speak Dutch, patients with missing data (particularly telephone number), patients who could not be contacted by telephone within 3 days of their ED visit, patients without access to e-mail and patients who refused to take part." | ED patients who met criteria | none specified |

For peer review only

| | | |
|---|---|---|
| "Inclusion<br>1. Published primary research<br>2. Information provided during critical care discharge to<br>general care area (ward)<br>3. Adult patients or family members receiving information<br>4. Evaluation of effects of information<br>5. Views of health care professionals<br>Exclusion<br>1. Discussion papers<br>2. Literature reviews<br>3. Descriptions of information" | mix of patients | hospital staff |
| N/A | N/A | N/A |
| none specified | none specified | none specified |

For peer review only

| | | |
|---|---|---|
| (1) 18 years or older, (2) acutely admitted at one of the four general medicine wards for more than 48 hours, (3) discharged home, (4) able to speak or understand Dutch, (5) have a working telephone, (6) showed no notification of cognitive impairment in the medical record, and (7) had an estimated life expectancy of more than three months. | those who met criteria | none specified |
| none specified | none specified | none specified |
| none specified | heart failure patients | cardio doctors |

For peer review only

| | | |
|---|---|---|
| "Inclusion criteria were that the participants were medical patients (non-surgical), sufficiently proficient in English that they could read and communicate without interpretation, were independent in their self-care, were to be discharged home and not to other facilities, and had a life expectancy greater than 6 months." | inpatients | hospital clinicians |
| >18 years.• Critical care stay (level 2 or 3) >72 hc | inpatients | hospital nurses |
| None specified | None specified | None specified |
| N/A | N/A | N/A |

For peer review only

| | | |
|---|---|---|
| N/A | N/A | N/A |
| none specified | none specified | none specified |
| none specified | none specified | none specified |
| "Patients were excluded for: high acuity/distress per the Attending ED physician, altered mental status, aphasia, developmental delay, dementia, or insurmountable communication barrier, non-English speaking patients, possible sexual assault, and acute psychiatric illness." | none specified | hospital staff |

For peer review only

| | | |
|---|---|---|
| "Inclusion: Adult patients (>18 years)<br>‣ Adult family members of eligible patients (>18 years)<br>‣ Elective or emergency admissions in the ICU ≥72 h<br>‣ Patients identified for discharge to a general ward setting within the hospital<br>‣ Elective discharges between 08:00 and 20:00 Monday to Friday<br>Exclusion criteria:<br>‣ Patients for whom active treatment had been withdrawn<br>‣ Inability to verbally communicate in or read English<br>‣ Involvement in a phase I focus group study4" | inpatients | hospital staff |
| N/A | N/A | N/A |

For peer review only

| | | |
|---|---|---|
| none specified | asthma patients, | none specified |
| none specified | chronic lower back pain patients | hospital nurses and clinicians |
| none specified | none specified | none specified |
| n/a | n/a | n/a |

For peer review only

| | | |
|---|---|---|
| "Exclusion criteria included all in-hospital deaths | none specified | none specified |
| "All consecutive patients of 18 years and older, a | inpatients | hospital staff |
| None specified | None specified | three consultants |

For peer review only - http://bmjopen.bmj.com/site/about/guidelines.xhtml

| | | |
|---|---|---|
| Exclusion: High-risk features in an electrocardiogram (ECG; e.g., ST elevation) and/or increased high-sensitive troponin levels (to exclude high-risk patients).<br>● None of the following cardiovascular risk factors: smoking history, diabetes, hypertension, dyslipidemia, age above 50 years, family history of CHD (to exclude lowrisk patients).<br>● Dementia, as defined by a score of <7 on a clock-drawing test.<br>● Age under 18 years.<br>● Limited German language skills (German being the default language at the hospital). | Chest pain patients | hospital physicians |
| N/A | N/A | N/A |

For peer review only

| | | |
|---|---|---|
| All adult patients who attended the clinic and were undergoing a category 1 (urgent) endoscopic procedure were eligible for the study. Participants were required to be able to read and write in English. | Eligible patients | 6 consultants |
| N/A | N/A | N/A |
| none specified | none specified | none specified |

For peer review only

| Findings/conclusion | Rigour/quality assessment (credibility and trusworthyness) | Relevance |
|---|---|---|
| it is duty to properly record clinical stay information and patient instructions | low | low |
| iving patients an information booklet at discharge from hospital appreciably increased the accuracy and thoroughness of their recall of important medical details concerning their illness and its treatment. The booklet was shown to be feasible, helpful in the outpatient clinic, and preferred by most general practitioners.' | medium | low |
| Giving an ipformation card to all patients at discharge was feasible and favoured by most patients and their general practitioners. Having made minor changes in design, we think that we have produced an information card that is a convenient size and will improve communication between patients, their general practitioners, and hospital doctors. We now issue this card routinely to all patients discharged from our ward and hope that it might be widely adopted.' | medium | very low |
| received no complaints from this practice. | low/medium | low |

For peer review only

| | | |
|---|---|---|
| "In our study, almost half the patients receiving bad news found their letter distressing to some extent; however, with 1 exception, all patients were pleased to have received it." "The study shows that letters to "patients are a useful method of improving patient satisfaction and recall in clinical consultations. They also provide a permanent record of the consultation, which can be kept for future reference, and encourage greater patient involvement in their care." | publication, rigour - med/high | medium |
| reports and documents should be shared with patients | low/medium | very low |
| "The recent introduction of new information technology to the NHS has not only improved the ability to carry out surgical audit, it has allowed the production of computerised discharge information on patients. This shorter, more structured form of discharge summary is clearly more acceptable to general practitioners and potentially may be used by all hospital specialties to provide a speedier, more efficient communication service to the general practitioner." | medium | medium |
| "On the basis of our findings we suggest that genetic counsellors send patients a letter summarising their consultations as this may result not only in an increase in the patient's understanding, but may prevent the miscom-munication of genetic information within the family." | Opinion based but published - low/med | low |

For peer review only

| | | |
|---|---|---|
| "Given that the majority of doctors supported the provision to some or all patients of summary letters, and the majority of oncologists supported the provision of a consultation audiotape in at least some cases, it may be useful to establish a forum where physicians and patients can develop guidelines for the use of these information aids. Such guidelines might suggest appropriate criteria for determining when to offer such aids, as well as a suggested format." | publication, rigour - med/high | medium |
| "For many people with eye disease, particularly if vision is threatened, fear of the unknown is a major cause of stress and anxiety. Carefully structured correspondence, written in a format that can be read easily and understood, can significantly enhance the management of many of our patients." | low/medium | low/medium |
| It is recommended that structured, pre-formatted instruction sheets be provided to all patients discharged to home, that emergency departments establish uniform policies to promote best practice in communication, and that the use of discharge instructions be considered as an emergency department performance indicator.' | publication, rigour - med/high | low |
| a competency based approach to test whether individuals have heard and understood their instructions may be better | medium | low/medium |

For peer review only

| | | |
|---|---|---|
| "The idea that patients should receive copies of letters and summaries is not new, and research has shown that it meets with high satisfaction from the patients." | Letter - low/medium | low-med |
| "Despite our wish to see this policy implemented nationally as soon as possible, we believe that it would be counterproductive to do this without first addressing the considerable concerns of both clinicians and certain patient groups." | Opinion based but published - low/med | low-med |
| "It should now be the exception to write letters that we would not wish patients to read." | low | low |
| "Patients receive a discharge information summary at the time of discharge." | medium/high | medium |
| "We agree with Chantler and Johnson that most patients (even those with cancer) wish to receive copy letters and most patients find them useful. However, it is essential to offer patients a choice in this matter because some patients do not wish to receive copy letters." | Opinion based but published - low/med | medium |
| Sending patients a copy of correspondence to their GP is one means of aiding communication and improving overall satisfaction.' | medium | medium/high |
| "letters between clinicians about an individual patient's care will be copied to the patient as of right" | medium/high | medium/high |

For peer review only

| | | |
|---|---|---|
| "These guidelines show that the issues to be tackled are mainly those already required throughout the health service and in good professional practice. They include good communication, obtaining consent, record keeping and handling (including IT support systems), and provisions to meet legal requirements to prevent discrimination on the grounds of disability or race. In particular, issues involve provisions around confidentiality and data protection." | medium/high | medium/high |
| "Patients found the intervention usable and reported increased confidence to ask questions. A definitive randomised controlled trial is required to establish the effect of the education package on patient outcomes." | medium | low |
| New training is needed in order to implement the practice of copying letters to patients - doctors are not yet ready | publication - high | medium |

For peer review only

| | | |
|---|---|---|
| Consultants who participated in the exercise did not perceive any additional difficulties in implementing this practice. This small study found that 100% of patients receiving a copy letter found it useful. The fact that around one-fifth of patients did not receive such copy letters within 2 weeks as intended is worrying, and requires further investigation. Sending a copy letter involves a relatively trivial cost for a practice which patients view as a valuable resource.' | publication, rigour - med/high | medium |
| "If the government is serious about providing more information to patients, then copying letters may be a useful way of facilitating that process of culture change, as well as addressing some of the objections to the principles of more information sharing." | Publication, NHS based. Rigour - high | low-med |
| "This study demonstrated that sending copies of the consultant oncologist letter to patients, proved to be a useful and valued method of communication with minimal addition to the workload." | publication, rigour - med/high | medium |
| "Our survey confirms that patients want to receive copy lettersand find it very useful. The beneficial effects outweigh thedrawbacks, which can easily be overcome. We suggest that the benefits of copying letters should be recognized and the process welcomed voluntarily rather than eventually responding to an imposed compulsory directive." | publication, rigour - med/high | medium |

For peer review only

| | | |
|---|---|---|
| There is diversity in medical and patient opinion about consultants writing directly to patients. These findings reflect fundamental differences in expectations about the nature and quality of communications between doctors and their patients.' | medium/high | medium |
| "The research demonstrated a high percentage of patients wanting a copy letter, which has huge resource implications in terms of secretarial time, additional stationery and postage costs. Change is difficult and an emotionally charged issue however, using examples of good practice and taking a multi-faceted approach to the change process the initiative has been successfully implemented." | medium/high | medium/high |
| 40% of patients wanted to see a copy of their clinic letter. Patients should be given a choice. | publication, rigour - med/high | low-med |
| "Patients appreciate copies of the letter being sent to their GP but comprehension is less good than with a shorter letter written especially to the patient. More attention needs to be paid to making letters to GPs simpler to read without losing the structure and detail liked by GPs. A compromise might be to dictate the letter in front of the patient and to provide a specialityspecific glossary to accompany each letter." | publication, rigour - med/high | medium |

For peer review only

| | | |
|---|---|---|
| "The GPs generally found the structure and lack of specific clinical detail in the letters to patients unacceptable. What we now need to do is to combine results from all of these reports and if only one letter is going to be written, determine the optimal format in terms of structure, content and comprehensibility to serve the needs of both referring doctors and patients." | high | high |
| "The findings suggest that although personal letters do not substantially improve recall of the clinical encounter, they are feasible, highly valued by patients and acceptable to referring clinicians." | high | medium |
| Patient receives copy promoting consumer "awareness and understanding" | medium | medium |
| "preparations are undoubtedly required to meet this new standard of care: after all, it seems to be strongly supported by patients in the NHS." | Publication, East Sussex Hospital. Rigour - high | med-high |
| "We have therefore demonstrated that the glossary may enhance a patient’s understanding of the letter to the GP and the positive, spontaneous comments would certainly suggest that it enhances patient satisfaction with the whole consultation process." | Publication, NHS based. Rigour - high | low |

For peer review only

| | | |
|---|---|---|
| "It appears that in general surgery in Wrexham these concerns may be more in keeping with patients' wishes than in other specialities where it is the professionals but not the patients who are concerned about more communication. Dissemination of this policy is a problem in that many junior doctors are not even aware of it, which is a training issue." | publication, rigour - med/high | medium |
| "We feel that the practice of writing letters directly to patients has numerous benefits. This study demonstrated how writing such letters allows the patients to have their own source of information about their management. We hope to encourage more clinicians, not just those within palliative medicine, to adopt this practice to further the goal of improving patient care." | publication, rigour - med/high | medium |
| "We suggest that rather than sending patients' copies of all their correspondence" as a routine, there are more secure and cost effective ways to inform patients and allow free access to all information recorded in their medical notes. | publication, rigour - med/high | medium |
| The results of this study will help inform how a DDS can be automatically generated from the electronic patient health record after each clinic visit.' | low/medium | very low |

For peer review only

| | | |
|---|---|---|
| patients like receiving copies of letters | medium | medium |
| "The majority of the patients found the copy of GP discharge letter helpful, informative, non-alarming and reassuring and wanted a similar communication in the future. The extra workload involved was minimal and the extra expense involved only an extra page, envelope and postage.' | medium | medium |
| "Copying patients into their GP letters is a practice supported by the majority of patients. It appears to have little impact on the running of the department and is likely to add significantly to the understanding that patients have of their consultation. Hospital departments should consider implementing this useful practice." | medium/high | medium/high |
| not in favour of patient copies of clinic letters "In the era of target dr | Opinion based but published - low/med | medium |
| "Before rushing to provide the service that Richards suggests, we must think it through, plan it properly to maximise its benefits to all patients, and decide whether the potential overall benefit is worth the cost." | Opinion based but published - low/med | medium |

For peer review only

| | | |
|---|---|---|
| "None of my consultant colleagues who has tried copying letters to patients has subsequently stopped because of the theoretical problems, and most, like me, have become converts to the practice. Try it and see." | Opinion based but published - low/med | low/medium |
| "Copying letters is well received amongst patients with CF, with numerous advantages and few disadvantages reported." | publication, rigour - med/high | low |
| "We conducted a pilot study to develop pictographs for discharge instructions through a participatory design process and test their efficacy in improving patient comprehension and recall. The pictograph enhancement resulted in better immediate and delayed recall rates. This suggests that we could improve patient comprehension and recall of discharge instructions by supplementing free texts with pictographs." | low/medium | low |
| All adult patients should have the option of receiving copy letters | medium/high | medium |

For peer review only

| | | |
|---|---|---|
| "This review suggests that researchers should move from examining the benefits and concerns around copying letters to patients, and instead focus on exploring the quality of correspondence and the optimum process of implementing the practice. As patients can ‘‘opt out’’ of receiving copy correspondence, audit of service delivery may be better assessed by whether patients have been offered a letter, rather than the current measure of whether one has been received.<br>Practice implications: Copying letters to patients may have a number of important benefits and should be routine practice where patients wish to receive correspondence. Further discussion regarding the style and content of letters would be beneficial, together with attention paid to the mechanisms for recording patient preference. There is also a need for studies in non-medical professions." | high | medium/high |
| "Many patients unnecessarily avoided activities of daily living after cataract surgery.<br>Providing an additional written sheet did not significantly improve this, whereas a photograph sheet did. Better awareness of the safety and rapid rehabilitation after modern cataract surgery is needed in hospitals and primary care centers." | medium/high | medium |
| patient letters with test results following discharge was beneficial by not inconviencing patients with a hospital visit and also ensuring test results were communicated quickly | medium/high | low/medium |

For peer review only

| | | |
|---|---|---|
| "This study demonstrated that a very high proportion of patients find copies of the outpatient letter helpful and easy to understand." | Publication, NZ - med/high | medium |
| "The ‘one size fits all’ approach of simply Table 4. Number of patients satisfied with present communication compared to all other types of communication by department. Copying letters should be seen as an adjunct to the communication process and not as a convenient substitute for it." | publication, rigour - med/high | med-high |
| "In conclusion, professional and patient scrutiny of letters, and writing letters which patients can understand, are simple but effective steps towards improving patient care." | low/medium | medium |
| outpatient correspondance does not meet readibility guidelines for the average population. It may not be possible to generate letters which are readible for patients and convey the necessary medical information to the GP | low/medium | low |
| education materials should be tailored to patient needs and record should be kept of the information given to patients | medium | very low |

For peer review only

| | | |
|---|---|---|
| "Effective discharge communication ensures smooth transition of patient from one health care setting to another to ensure continuity of care. This study demonstrated that there are effective strategies to improve discharge communication, Health care providers, both public and private could adopt, adopt and implement an effective approach for discharge communication in hospitals, as a means towards patient safety." | low/medium | very low |
| Only 60% of patients receive their discharge letters and are aware of medication information. | medium | medium |
| "Supported living: high score means living in supported accommodation, attending day care, having more letters copied to user." | medium | very low |
| "This study demonstrated that patients commonly remain confused about aftercare information following treatment in an ED. Follow-up telephone calls may be useful for identifying and addressing ongoing learning needs." | publication, rigour - med/high | low |

For peer review only

| | | |
|---|---|---|
| "It is not considered to be good practice to send the discharge summary home with the patient as there is no guarantee that the information will be passed on to the general practitioner." | medium | very low |
| Giving patients a written discharge plan | medium | low |
| "Including information in a discharge summary in native languages significantly improved patients' knowledge of illness and medication. This could be a simple and cost-effective method to improve health communication and health knowledge. This should be replicated in other parts of Sri Lanka and in other countries with similar contextual factors and further evaluated." | medium | very low |
| "Structured teaching of communication with the patient brings family medicine back to what has actually always been its main part – communication and doctor-patient relationship. Our future aim is to develop students' letters to patients as a new tool in the family medicine course examination. Moreover, we will investigate how they can be used in everyday practice of family medicine." | medium | low |

For peer review only

| | | |
|---|---|---|
| "Patients had poor to moderate understanding of their test results and recommendations at discharge. The simple brief intervention of a patient directed discharge letter significantly increased immediate patient understanding in three of the four major domains.We are currently collecting follow-up data at three and six months to assess retained knowledge and clinical outcomes." | high | high |
| "The study clearly demonstrates that sending a copy letter to patients does help them to understand their condition better, contrary to the misconception amongst health professionals. Hence all hospital departments should consider implementing this useful practice." | medium/high | high |
| "Implementation of a DL improved compliance with surveillance at 1 year of follow-up following discharge from a tertiary cancer centre, although optimal adherence remains low. However, adherence to CT imaging nearly doubled. Evaluation of compliance at 3 years of follow-up is ongoing." | medium | medium |
| "Although most of these studies have shown that receiving the GP letter is welcomed greatly by patients, results should be interpreted with caution." | publication, rigour - med/high | medium |

For peer review only

| | | |
|---|---|---|
| The majority of patients want to receive a copy of their clinical letters. "There is a substantial risk of breaching patient confidentiality when distributing correspondence by post. A well-designed security arrangement is therefore required to ensure the safety of confidential information. Despite the security concern, mail is still the preferred mode of delivery." | publication, rigour - med/high | medium |
| "There is considerable evidence and experience to suggest that patients receive good quality letters very positively and with the outcome of improved satisfaction and reduction of anxiety. However, the most pressing implication of the policy relates to those sending the letters if they do not prepare properly and patients receive inappropriate or unsuitable letters, which might cause unnecessary distress or concern." | low/medium | low |
| "A key limitation of the present study is that only a small number of experts were used to drive the iterative refinement; this cannot be expected to capture all the problems that health consumers might encounter and does not provide a quantitative measure of performance." | low/medium | very low |
| "standard headings for the clinical information that should be recorded in the discharge record and included in the discharge summary communication from hospital to GP and patient." Information and advice given to patient should be provided in written discharge communication | medium/high | low |
| "Writing of a letter to their first patients may be a useful tool for students to personally experience the practice of medicine and establish better partnership with patients in health care." | medium | low |

For peer review only

| | | |
|---|---|---|
| "The new template for drug information was superior to the standard discharge letter with regard to readability and usefulness for the patients, and contributes to better overview of drug changes at discharge." | low/medium | very low |
| "Results of this study support the hypothesis that it makes sense to provide patients with written instructions about the appropriate use of analgesics, and that emergency departments that are not yet doing this should consider introducing this policy. It is a relatively low-cost measure that could lead to a significant improvement in quality of care." | medium | very low |

For peer review only

| | | |
|---|---|---|
| "Internationally, evidence evaluating critical care discharge information is scant. Many complex and interwoven factors can affect physical and psychosocial health outcomes after discharge from critical care, making it difficult to extrapolate the effects of information giving alone. Our understanding of service users' perspectives in this important area also is quite limited. Findings of this review should inform the further development of information for this population and the design of future studies investigating this neglected area of critical care practice. CCN" | high | medium |
| The law requires written and oral information to be given to patients (France). The written form is often not used. | medium | medium |
| "Advice given to patients should be in language understandable to the individual6,31,33 and cover aspects of the treatment plan including self-management that encourages a shared-care model of health." | publication, rigour - med/high | very low |

For peer review only

| | | |
|---|---|---|
| The comprehensive discharge bundle was not effective in reducing the rate of readmission and increasing patient satisfaction, but medical discharge summaries were sent faster to the general practitioner and a trend to a longer time to readmission was present.' | publication, rigour - med/high | very low |
| The current methods of delivering ED discharge instructions are not optimal for patient learning. Proper health education for patients after an ED visit has a potential to improve patient understanding of health information, decrease unnecessary return visit to the ED, and positively affect health' | medium | low |
| HF patients' comprehension of discharge instructions is inadequate. Patients with limited education and those that do not speak English as a primary language are more likely to have poorer discharge understanding and higher rates of 30-day readmissions.' | medium/high | low |

For peer review only

| | | |
|---|---|---|
| "A simple patient-directed letter delivered during a brief discussion improves patient understanding of their hospitalisation and post-discharge recommendations, which is otherwise limited. Further evaluation of this brief and well-received intervention is indicated, with the goal of improving patient understanding, satisfaction and clinical outcomes." | high | high |
| "Patient discharge summaries are likely to be a useful adjunct to existing discharge information, but further work is required to determine when and how they should be provided. With appropriate training and support, it is feasible for nurses to write dischargesummaries in a busy critical care environment." | high | high |
| "patients have a right to these hospital reports, and it is the responsibility of physicians to provide them." | medium | medium |
| Ask patient how they prefer to receive information. "Use discharge instructions that meet health literacy needs. Materials should be written at a 5th grade or lower reading level. Consider revising written materials to address the health literacy needs of all patients. Use readability tests, divide complex information into bullet points, and modify document font, layout, and design to revise written materials to improve readability." | medium | low |

For peer review only

| | | |
|---|---|---|
| Patient discharge summaries are not routinely produced despite the reported benefits for patients on receiving this information | medium | medium |
| Therefore, nursing and hospital leaders must develop and have a formal discharge planning process in place that includes interprofessional collaboration to effectively meet the treatment needs of patients and achieve adequate healthcare results.' | medium | very low |
| Consider the patient's learning style19,20 | medium | low |
| "Patient understanding of discharge instructions is an important aspect of ED provider–patient communication and adequate care. Our investigation reveals possible areas of communication as well as specific patient populations to target to ensure proper and safe understanding." | medium/high | medium |

For peer review only

| | | |
|---|---|---|
| "Findings from this pilot RCT provide important preliminary data regarding the circumstances under which an intervention based on the principles of UCCDIP could be effective, and the sample size required to demonstrate this." | high | medium |
| "The form, designed to be completed by junior doctors as part of discharge processes, includes a text box entitled “clinical narrative” which asks the discharging clinician to tell the story of the admission, encouraging them to do so in a way that might be easily understood. Patients themselves receive a printed copy at discharge, aiming to reinforce the importance of making the narrative readable. The documentation also includes the list of medications on which a patient has been discharged as well as specific boxes to document any medications that have been discontinued and any changes made to dosages, flagging up those factors most important for a GP to have quick sight of." | medium | medium |

For peer review only

| | | |
|---|---|---|
| Participants across literacy groups and settings identified multiple actionable areas for improvement in the ED discharge process. These included the use of simplified/lay language, increased visual learning and demonstration, and the desire for complete information. Individuals with limited literacy may particularly benefit from increased attention to consistency.' | medium | low |
| "We believe that if we are able to provide clear and understandable instructions at discharge we might partially reduce inappropriate usage of the ED due to chronic disease, reduce costs and satisfy the patients' needs." | medium/high | low |
| patient summaries improve patient understanding and decrease queries received by hospital followng patient discharge. | medium/high | medium |
| "Involving patients at discharge has been shown to be valuable in reducing medication-related readmissions and post-discharge service utilisation; for improving patient outcomes and for supporting patients in understanding how, when, and where to seek help should they need it." | medium/high | medium |

For peer review only

| | | |
|---|---|---|
| "Patient discharge notes are written at too advanced of an educational level. To ensure patient comprehension, dismissal notes should be rewritten to a 6th-grade level." | medium/high | low |
| "Patients and professionals rated the PPDL positively. Key success factors for implementation were: education of interns, residents and staff, standardization of the content of the PPDL, integrating the PPDL into the electronic medical record and hospital-wide policy." | medium/high | high |
| "While some oncologists assess the copy letters as inappropriate for supplemental patient-oncologist-communication, breast cancer patients regard this tool as predominantly gainful. Oncologists appear to stick to their traditional perspective which perceives the copy letter mainly as a communication tool from doctor to doctor. Due to their individual experience, patients seem to develop an emotional relationship to the copy letter containing information about their disease. Especially for patients dealing actively with their treatment process, copy letters could be a reasonable instrument." | medium | medium |

For peer review only

| | | |
|---|---|---|
| "Effective discharge communication, empowering patients to understand and memorize medical information, should therefore be an integral part of patient care. It is a likely contributor to better outcomes (Bishop, Barlow, Hartley, & William, 1997; Kessels, 2003), higher patient satisfaction (Kessels, 2003), better adherence to medication (Cameron, 1996; Kessels, 2003), more adequate disease management, and reduced anxiety (Galloway et al., 1997; Mossman, Boudioni, & Slevin, 1999)." | medium/high | medium/high |
| "When asked how often patients are discharged home with a copy of their discharge plan to give to the community nurse, 48.2% (n=242) of community respondents said that patients 'sometimes' had a copy, 26.5% (n=133) said patients 'almost always' had a copy and just 1% (n=5) responded that patients are always given a copy of their plan." | medium/high | medium |

For peer review only

| | | |
|---|---|---|
| "Patients wish to receive copies of their correspondence and feel it improves their understanding of their medical condition. Although we were unable to demonstrate a measurable reduction in anxiety, increase in understanding or satisfaction, we recommend that patients be offered the choice of receiving copies of their clinic correspondence and endoscopy reports." | publication, rigour - med/high | low-med |
| "Of course there are situations where writing to patients may be inappropriate; breaking bad news is always better face to face. But patients do not want any of the consultation withheld from letters." | low/med | medium |
| "It is acknowledged that outpatient letters are increasingly written to patients and copied to GPs. In these cases the letter can act as a contract between the patient and their healthcare professional. It is expected that the standards will be applicable to these letters also." | medium | low |

For peer review only

## CMO Table

| | Context | Mechanism | Outcome | Effect assessment | Does it "work" or not? |
|---|---|---|---|---|---|
| CMOC1 | patient not offered letter | patient feels less involved in care | reduced patient autonomy | negative | does not work |
| CMOC2 | patient offered opportunity to receive letter(s)/patient choice respected | patient feels more informed and involved in care | increased patient autonomy and increased involvement of patients in treatment, care and communications | positive | does work |
| CMOC3 | large clear posters displaying patients right to choose and importance of correct contact information | patient realises they should inform hospital of address changes and preferences | lowered risk of confidentiality breach | positive | does work |
| CMOC4 | NHS drive for patient-led care (influence or context) | clinicians increasingly offering patient choice of receiving letter/sharing information with patients | increased patient empowerment | positive | does work |
| CMOC5 | clinician views letters to patients are beneficial e.g. increases transparency, compliance, trust, patient satisfaction, patient understanding and recall | clinician feels patient should be offered letter | potential increase in patient autonomy & satisfaction | positive | does work |
| CMOC6 | Clinicians views letters to patients as not beneficial e.g. letter not comprehensible to patient, medico-legal issues, increased cost and staff workload, patient harm (anxiety, distress, and confusion) and issues around confidentiality | clinician feels patient should not be offered letter | no patient autonomy | N/A | unclear |
| CMOC7 | NHS guidance that all hospital-GP correspondence should be copied to patient as a "right" where appropriate and if patients agree (unless risk of serious harm or legal issues) | clinicians increasingly offering patient choice of receiving letter | increased use of NHS resources to send letters but patient benefits through increased understanding & potential reduction in patient queries (costs balanced) | positive | does work |
| CMOC8 | Data Protection Act 1998 (UK) | Patients may become aware of their right to know what is written & stored about them | Patients informed of their stored electronic information (increased transparency) | positive | does work |
| CMOC9 | doctors copy patients letters | patient trusts doctor more | improved doctor-patient relationship | positive | does work |
| CMOC10 | patients offered choice of receiving letters | increased no. of patients choosing to receive letters | Increased administrative staff workload and costs of printing & posting letters | negative | unclear |

For peer review only

| | Context | Mechanism | Outcome | Effect assessment | Does it "work" or not? |
|---|---|---|---|---|---|
| CMOC11 | patients offered choice of receiving letters | increased no. of patients choosing to receive letters | reduced queries and GP visits and reduced hospital re-admissions (limited evidence) | positive | does work |
| CMOC12 | structured discharge letters written clearly in plain English (pref. 5th grade level) with medical jargon defined, no value judgements of patients and minimal abbreviations | patients understand letter | increased patient knowledge | positive | does work |
| CMOC13 | doctors provided training in letter writing & record keeping (contextual influence) leading to doctors write letters of higher quality and more appropriate for patients | patients understand letter | Increased patient knowledge/potential increase in doctor confidence in letter writing | positive | does work |
| CMOC14 | patients preference for letter copies acknowledged and patients offered choice of receiving letter | patients feel able to express their preference | decreased strain on resources & increased patient autonomy & satisfaction | positive | does work |
| CMOC15 | patients provided written & verbal information | patients reflect on written record of information for reference | increased patient knowledge of care plan, recall and acceptance of illness or condition | positive | does work |
| CMOC16 | Human Rights Act (1998) and Race Revelations Act (2000) - clinicians equally offer all patients letter copies regardless of background | clinician feels all patients should be offered letter | increased equality and accessibility of information to patients | positive | does work |
| CMOC17 | Use of pictures/pictographs/cartoons with written information | patients understand letter | Patient benefits from improved understanding e.g. adherence to agreed care plan | positive | does work |
| CMOC18 | verbal information only | patient may not be able to retain information | reduced patient recall | negative | does not work |
| CMOC19 | professionals who are not involved/limited involvement with patient writes letter | professional does not understand patient plan | letter quality reduced/increased risk of harm | negative | does not work |
| CMOC20 | patient hospital visit of sensitive nature and/or patient lacks capacity e.g. psychotic episode, dementia | patient finds letter distressing and/or confusing | harm to patient | negative | does not work |
| CMOC21 | Patient letter written above patient educational level or in a language the patient does not read | patient finds letter difficult to understand | patient is confused with no increased knowledge of care/possible misinterpretation of care instructions | negative | does not work |
| CMOC22 | letter contains inaccurate information | patient identifies inaccuracies | patient notifies hospital/GP of inaccuracies and corrections are made leading to improved record keeping | positive | does work |

For peer review only

| | Context | Mechanism | Outcome | Effect assessment | Does it "work" or not? |
|---|---|---|---|---|---|
| CMOC23 | patients receives discharge letter | patient does not understand entirety of letter | patient sources answers (internet, GP, friend or relative) | positive | does work |
| CMOC24 | Patient specific letter sent to patient | patient finds letter clear | improved patient comprehension/patient may use letter as aid to explain condition to family and friends | positive | does work |
| CMOC25 | Patient specific letter sent to patient | | increased staff workload and costs | negative | does not work |
| CMOC26 | Patient specific letter sent to patient | Patient identifies information sent to GP and patient is different | medico-legal concerns could be raised over letter discrepancies and any withheld information | negative | does not work |
| CMOC27 | hospital sends patient discharge letter without verifying patient contact details without notifying patient | hospital worker does not identify and correct incorrect information | potential breach of patient confidentiality | negative | does not work |
| CMOC28 | hospital routinely checks patient addresses and sends discharge letters to patients marked confidential using full name | hospital worker identifies and corrects incorrect information | patient receives letter, minimal risk of patient confidentiality breach | positive | does work |
| CMOC29 | patient receives discharge letter | patient may feel they have questions relating to letter | patient contacts health provider with queries (evidence suggests minimal impact and queries) | positive | unclear |
| CMOC30 | discharge letter/summary dictated in front of patient | patients query any inaccuracies | letter less likely to contain inaccuracies | positive | does work |
| CMOC31 | Hospital gives patient discharge letter/summary to deliver to GP | patient may find they are unable to make delivery | GP does not always receive letter/summary | negative | does not work |
| CMOC32 | Patient receives letter not written at appropriate level for them | patient feels confused and does not understand letter | GP spends time reassuring patient and explaining letter to ease patient upset | negative | does not work |
| CMOC33 | Patients have anxiety that doctors talk about things behind their backs | patients who receive letter feel reassured that there is no hidden information | decreased patient anxiety and improved doctor-patient relationship through transparency | positive | does work |
| CMOC34 | patients receives discharge letter | Patients feel they are important to clinician | patient is impressed with letter and feels clinician has an interest in them | positive | does work |
| CMOC35 | choice about whether letter is sent to patient | clinician feels letters would be a disaster and inappropriate for patients | patients do not receive letters | N/A | unclear |
| CMOC36 | patients receives discharge letter | | no impact on patient | N/A | unclear |
| CMOC37 | patients receives discharge letter with bad news | Patient finds letter initially distressing | letter causes initial distress but final outcome that patient finds letter helpful and aids recall and acceptance of condition | positive | does work |

For peer review only

| | Context | Mechanism | Outcome | Effect assessment | Does it "work" or not? |
|---|---|---|---|---|---|
| CMOC38 | letter sent to patient containing information not discussed with patient or abnormal results | patient feels distressed and anxious reading letter | patient harm/unethical practice | negative | does not work |
| CMOC39 | patient worried about diagnosis and receives letter | patient understanding helped by letter | patient feels less anxious due to being more informed | positive | does work |
| CMOC40 | patients preference for letter copies not acknowledged | | patient may receive letter who didn't want one leading to decreased patient satisfaction | negative | does not work |
| CMOC41 | (best practice) patients offered choice of receiving letters/opt-in system | patients enabled to decide on letter preference | patients may or may not receive letter depending on their preference resulting in higher patient satisfaction | positive | does work |
| CMOC42 | patients who feel copies of letters are not necessary for themselves | | patient not given letter so patient satisfied, secondary outcomes: costs and time saved | positive | does work |
| CMOC43 | patients receives discharge letter where appropriate | patient understands letter (high evidence) | patient finds letter informative and helpful | positive | does work |
| CMOC44 | patients receives discharge letter where appropriate | patient feels involved in care plan | patient ensures follow up plan is followed and books any necessary tests etc. | positive | does work |
| CMOC45 | patients receives discharge letter where appropriate | patient feels letter is important | letter forms permanent record of hospital visit and kept for future reference | positive | does work |
| CMOC46 | patients receives discharge letter for breaking good news | patient reminded of discussion | patient feels reassured and has "peace of mind" | positive | does work |
| CMOC47 | patients receives discharge letter where appropriate (patient choice) | patient likes receiving letter | patient satisfaction increased | positive | does work |
| CMOC48 | patients receives copy of discharge letter where appropriate | patient becomes aware of what GP knows | Patient reassured that GP knows about visit | positive | does work |

For peer review only

BMJ Open

# Improving best practice for patients receiving hospital discharge letters: a realist review



| | |
|---|---|
| Journal: | *BMJ Open* |
| Manuscript ID | bmjopen-2018-027588.R1 |
| Article Type: | Research |
| Date Submitted by the Author: | 25-Feb-2019 |



| | |
|---|---|
| Complete List of Authors: | Weetman, Katharine; University of Warwick Warwick Medical School, Unit of Academic Primary Care<br>Wong, Geoffrey; University of Oxford Medical Sciences Division, Nuffield Department of Primary Care Health Sciences<br>Scott, Emma; University of Warwick Warwick Medical School, Unit of Academic Primary Care<br>MacKenzie, Eilidh; University of Warwick Warwick Medical School<br>Schnurr, Stephanie; University of Warwick, Centre for Applied Linguistics<br>Dale, Jeremy; University of Warwick Warwick Medical School, Unit of Academic Primary Care |



| | |
|---|---|
| <b>Primary Subject Heading</b>: | Communication |
| Secondary Subject Heading: | Communication, Patient-centred medicine, Health services research |
| Keywords: | communication, hospital discharge, patient information, discharge letter, realist review |
| | |


SCHOLARONE™ Manuscripts

# Improving best practice for patients receiving hospital discharge letters: a realist review

**Katharine Weetman[1], Geoff Wong[2], Emma Scott[3], Eilidh MacKenzie[4], Stephanie Schnurr[5], Jeremy Dale[6]**

1. Unit of Academic Primary Care, Division of Health Sciences, Warwick Medical School, University of Warwick, Coventry, UK, CV4 7AL, **Doctoral Researcher**
2. Nuffield Department of Primary Care Health Sciences, Medical Sciences Division, University of Oxford, Oxford, UK, OX2 6GG, **Clinical Research Fellow**
3. Unit of Academic Primary Care, Division of Health Sciences, Warwick Medical School, University of Warwick, Coventry, UK, CV4 7AL, **Senior Research Fellow**
4. Warwick Medical School, University of Warwick, Coventry, UK, CV4 7AL, **Medical Student**
5. Centre for Applied Linguistics, University of Warwick, Coventry, UK, CV4 7AL, **Associate Professor**
6. Unit of Academic Primary Care, Division of Health Sciences, Warwick Medical School, University of Warwick, Coventry, UK, CV4 7AL, **Professor**

Correspondence to:
Katharine Weetman
Warwick Medical School, University of Warwick, Coventry, UK, CV4 7AL
K.Weetman@warwick.ac.uk
+44 (0)2476 151077

Word count, excluding title page, abstract, bibliography, figures & tables:

6,987

Word count, excluding title page, headings, abstract, references, figures & tables and quotes:

5,748

**Key words:** Communication; discharge letter; hospital discharge; patient information; realist review

**Study area:** General health



For peer review only - http://bmjopen.bmj.com/site/about/guidelines.xhtml

For peer review only

**TITLE**

Improving best practice for patients receiving hospital discharge letters: a realist review

**ABSTRACT**

**Objective:** To understand how different outcomes are achieved from adult patients receiving hospital discharge letters from inpatient and outpatient settings.

**Design:** Realist review conducted in six main steps: 1) Development of initial theory 2) Searching 3) Screening and selection 4) Data extraction and analysis 5) Data synthesis 6) Programme theory (PT) refinement.

**Eligibility criteria:** Documents reporting evidence that met criteria for relevance to the PT. Documents relating solely to mental health or children aged <18yrs were excluded.

**Analysis:** Data were extracted and analysed using a realist logic of analysis. Texts were coded for concepts relating to context, mechanism, outcome configurations (CMOCs) for the intervention of patients receiving discharge letters. All outcomes were considered. Based on evidence and our judgement, CMOCs were labelled "positive" or "negative" in order to clearly distinguish between contexts where the intervention does and does not work.

**Results:** 3113 documents were screened and 103 were included. Stakeholders contributed to refining the PT in step 6. The final PT included 48 CMOCs for how outcomes are affected by patients receiving discharge letters. "Patient choice" emerged as a key influencer to the success (or not) of the intervention. Important contexts were identified for both "positive" CMOCs (e.g. no new information in letter) and "negative" CMOCs (e.g. letter sent without verifying patient contact details). Two key findings were that patient understanding is possibly greater than clinicians perceive, and that patients tend to express strong preference for receiving letters. Clinician concerns emerged as a barrier to wider sharing of discharge letters with patients, which may need to be addressed through organisational policies and direction.

**Conclusions:** This review forms a starting point for explaining outcomes associated with whether or not patients receive discharge letters. It suggests several ways in which current processes might be modified to support improved practice and patient experience.

**Strengths and limitations of this study:**

- First study to review and develop realist theories about patients receiving discharge letters.
- The engagement of patients, GPs and commissioners in refining the programme theory increased relevance and rigour of the theory.
- The programme theory is likely to be applicable and relevant to multiple healthcare settings.
- The exclusion criteria imposed restrictions on the programme theory such that evidence relating to children, solely to mental health, and those lacking capacity is not considered.
- Only sources written in the English Language were included.



For peer review only - http://bmjopen.bmj.com/site/about/guidelines.xhtml

# INTRODUCTION

## Background

*Discharge communication* may follow an inpatient or outpatient discharge; it typically comprises written discharge information in the form of a discharge letter or summary. It is a well-established practice that the physician who is to follow up patient care, typically the General Practitioner (GP) or equivalent [(1)], should receive written *discharge communication* from the discharging physician; this practice supports continuity of care between specialist services and primary care. Patients are sometimes included in this communication, and while within the UK this is considered to be 'good practice' [(2)], is not standardised.

The *Department of Health* in the UK describes patient copies of letters as a "right" [(3)] and recommend patients should be copied in where appropriate as a "rule", unless there is risk of harm [(2, 3)]. This is intended to support patient understanding and wellbeing, increase patient safety and the quality of information sent, and improve doctor-patient relationships [(2-4)]. More recently, the *Academy of Medical Royal Colleges (AMRC)* released the "please write to me" [(5)] initiative. The initiative encourages doctors to write directly to patients in simple plain English to increase understanding. Despite these initiatives and guidelines, evidence within and outside the UK reports both benefits [(6-10)] (e.g. patient satisfaction), and drawbacks [(11-15)] (e.g. patient confusion) of patients receiving their letters. While patients receive copies of discharge letters inconsistently [(16, 17)], the reasons for this and the subsequent consequences remain unclear.

Hence, the objectives of the current study were to conduct a realist review of patients receiving discharge communication (the intervention); to develop a programme theory (PT); and to make best practice recommendations. The research questions (RQs) were:

RQ1: What positive and negative outcomes have been reported on patients receiving written discharge communication?

RQ2: What are the important contexts which are associated with whether the mechanisms produce the different outcomes, and why?

# METHODS

A realist review is a, 'theory-driven, interpretative approach to the synthesis of evidence' [(18)]. Synthesising evidence involves interrogating data sources to develop, refine and test *context, mechanism,* and *outcome* configurations (CMOCs). "Context" may be conceptualised as external factors that influence mechanisms [(19)]. "Mechanisms" are hidden, context sensitive causal forces that produce "outcomes" [(19)]. Following Pawson [(19-22)], CMOCs should be configured and consolidated to build and develop a realist *programme theory* or theorised explanation of how an intervention *works* or not. The intervention under scrutiny 'patients receiving discharge letters' was defined by the review team as 'the patient being given or sent any form of written (paper or digital) hospital discharge communication; this could be a direct copy, patient-directed letter, or a combination.' The aim of the review is to understand and explain how the different outcomes are produced for adult patients receiving written discharge letters. Outcomes may be simplified into desired/beneficial or 'positive' (e.g. increased satisfaction) and undesired/detrimental or 'negative' (e.g. increased anxiety).

We have previously published the full protocol for this review [(23)] which justifies the rationale for a realist approach and considers each of the methodological steps in detail. The overall review design was informed by previous literature, driven by the research questions,



For peer review only - http://bmjopen.bmj.com/site/about/guidelines.xhtml

consists of six steps [19, 24, 25] and is further described in the protocol paper [23]. This design is summarised in figure 1.

*Figure 1 Review design*

**Programme theory development (step 1)**

The task of locating existing theories to develop an initial rough PT was achieved through a scoping search. Theories and evidence were sought which aided explanation of how and why patients receiving discharge communication results in different positive effects (e.g. drug adherence) and negative effects (e.g. preventable hospital readmissions). Sources were selected based on their "relevance" [19-21] to the PT; where *relevance* concerns 'does the [source] address the theory under test?' [20]. Crucially, the whole source did not need to inform the PT but we considered the relevance and contribution of sections of the document [20].

Search terms were based on the intervention (e.g. patient cop(y)ies). Published resources and healthcare websites were searched to ascertain a range of evidence (see *Supplementary file 1*). During this phase, research team judgement was needed to decide the stopping point for programme theory development as was the need to balance the degree of comprehensiveness and practicalities [23]. As the purpose was to locate existing theories and initial concepts, the search was not intended to be comprehensive and the decision was made to screen no more than 30 documents. During the scoping search, search strategies within articles and article indexing were noted in order to inform a more thorough subsequent search in step 2.

Twenty seven documents were selected from the scoping search (see *Supplementary file 2*). All documents were then interrogated and coded for any CMOCs, concepts, or theories which could inform development of a PT. These were consolidated to form Figure 2, the initial PT.

The initial PT shows two main channels for discharge communication; patient copied into (or not) the hospital to GP letter and patient received a personalised letter. Limited evidence was available for the option of 'patient does not receive copy' as evident in Figure 2. Patients being copied into discharge letters, whether by choice or otherwise, is associated with a large range of mechanisms and outcomes. Contexts such as 'patient literacy level' are likely to influence generation of mechanisms (patient does/does not find letter comprehensible) and outcomes (increase/no increase in patient knowledge), but this was unclear from the evidence reviewed in the scoping search. The scoping search revealed a range of "contextual influences" (e.g. 'time constraints of writer' may affect whether a patient is given a choice about receiving a letter and also the overall letter quality). It was unclear where some CMOCs began and ended (e.g. "is patient given choice about receiving copy of discharge communication" falls between contextual influences and context labels). In Figure 2, f/u stands for "follow up" and the small circled "c" icon refers to an outcome which could also thereafter take the form of a context. In summary, there were clear "gaps" and information missing from the initial PT, confirming that the scoping search in isolation was



For peer review only - http://bmjopen.bmj.com/site/about/guidelines.xhtml

insufficient for realist theory generation; further evidence and searching was needed to clarify details and simplify CMOCs (steps 2-6).

*Figure 2 Initial Programme Theory*

**Search strategy (step 2)**

The electronic searching was purposive and guided by the initial PT, results and indexing from step 1. A search strategy was developed which was piloted and adapted for MEDLINE until a diverse and relevant range of search results were yielded (target 500-3,000). In line with a realist approach, searching was iterative, and the strategy was refined for each database (see *Supplementary file 3*). Sources included electronic databases, healthcare sites, and grey literature.

The search strategy was not intended to be exhaustive, but provided a large enough overview to be meaningful for PT development [20]. Evidence was searched up until September 2017; publications were monitored thereafter but no new evidence affected the PT. In total, 3113 documents were selected for screening.

**Selection and appraisal of documents (step 3)**

Inclusion or exclusion of source evidence for the review were according to the following criteria:

**Inclusion criteria:**

• Full text or section of source had *relevance* [19, 20] to informing the PT
• Relate to inpatients/outpatients discharged from general hospital setting to GP (or equivalent)
• Relate to discharge where ‘written discharge communication’ is sent to GP or referring physician (may also be copied to patient)
• Source written or published in English

**Exclusion criteria:**

• Specific to discharge to units/physicians other than GPs (or equivalent), e.g. another hospital
• Specific to discharge of patients who lack cognitive capacity, e.g. dementia, or where there may be higher risk of harm, e.g. mental health discharge
• Lack of written communication having taken place, e.g. telephone only
• Specifically relate to patients <18 years
• Source not written or published in English

For peer review only



For peer review only - http://bmjopen.bmj.com/site/about/guidelines.xhtml

The exclusion criteria posed limitations on the review; children under 18 (where the parent would often be the letter recipient), patients with particularly specialised communicative needs (e.g. patients without capacity) or where the intervention may have a higher potential risk of causing harm (e.g. psychiatric discharge documents) were excluded. The communication needs of some of these patients may be more complex and variable within and between groups and therefore was not possible within review scope. The first exclusion criterion states patient discharge communication to those other than GPs or equivalent (e.g. family or community physicians) was excluded. This is because the review specifically focussed on discharge communication to GPs and patients rather than referrals or care-handovers. Furthermore, the review aimed to develop a theory for patients receiving discharge communication and inclusion of hospital-hospital discharge may have reduced clarity and produced a less focussed theory.

Once KW had screened the documents by title and abstract, second reviewer EM screened a random 10% test selection; this proportion was selected following Wong et al. [24]. Inter-reviewer agreement was set at kappa measure K≥.8 [26]. A result K<.8 would require all documents to be second screened. Inter-reviewer agreement was calculated as sufficient (K=0.82). In the first screening phase, 611 duplicates were removed and 2,341 documents excluded; this left 161 documents.

The full texts of these 161 documents were then screened, primarily for relevance [19, 20] by KW, with EM screening a random 10% sample. Inter-reviewer agreement was again sufficient (K=0.92). Eighty eight documents were excluded at this stage leaving 73 for inclusion.

In addition, hand-searching of bibliographies, 'cited by' searching, and contacting experts was undertaken. This identified a further 30 relevant documents, creating a total of 103 documents. *Supplementary file 4* provides the final document list. The selection process is summarised in Figure 3.

*Figure 3 PRISMA [27] diagram (document selection process)*

**Data extraction and analysis (step 4)**

A hybrid approach to data extraction was undertaken [24, 28, 29]. This allowed extraction of both descriptive document characteristics and annotation of CMOC ideas for synthesis and integration into the PT [19, 20]. A data extraction form was designed iteratively to record pertinent document details. Final columns included: author(s), year, geographical information, healthcare system, design aim, no. of participants intervention, clinical speciality, inclusion and exclusion criteria, findings/conclusions, rigour/quality assessment [19, 20], topic focus, form of discharge communication e.g. discharge summary, participant mix, staff mix, and relevance score [19-21].

Documents were also annotated in NVivo for CMOCs and PT ideas. Annotations were guided by the initial PT devised in step 1.

**Data synthesis (step 5)**

During step 5, data and annotations of PT ideas and CMOs were consolidated. A realist analytic approach, following the work of Pawson [19-21, 30], was used to interrogate the theory during data synthesis. Pawson [19-21, 30] presents several different frameworks for synthesising data evidence. We selected the framework [20] entitled "synthesis to consider the same theory in comparative settings", which involves five analytical strategy steps. This



For peer review only - http://bmjopen.bmj.com/site/about/guidelines.xhtml

framework was chosen as it assumes theories sometimes "work" and "do not work" according to the particular setting; Pawson et al. [20] describe this as 'aim[ing] to make sense of the patterns of winners and losers'. Hence, this framework is suitable for the research questions which focus on cause and context of positive outcomes "winners" and negative outcomes "losers". Thus, data synthesis was grounded on the assumption that the *outcomes* of the intervention may differ according to *context*.

The following realist analytical strategy steps [20, 28, 31] were undertaken simultaneously:

1. Juxtaposition of data sources – align sources to build upon/clarify each other
2. Reconciliation of data discrepancies – explore reasons for data disparities
3. Adjudication of data – data quality consideration of trustworthiness/relevance
4. Consolidation of data - inference of Mechanisms for outcomes
5. Situation of evidence - consideration of intervention settings

Data synthesis using the analytic strategy 'juxtaposition of data sources' was achieved through utilisation of NVivo 'nodes'. Sections of text were annotated, and coded as nodes. The nodes were named according to ideas or concepts around the programme theory and contained sections of text that were used to build CMOCs. NVivo node coding resulted in 19 nodes seen in Table 1.

*Table 1 Coding nodes*

| Node name | No. of different sources coded | Total no. of sections of text coded |
|---|---|---|
| Autonomy | 5 | 5 |
| Clinician context (views) | 23 | 57 |
| Confidentiality | 12 | 15 |
| Context (when it does not work) | 29 | 46 |
| Context (when it does work) | 54 | 107 |
| Cost/resources | 20 | 33 |
| Dictate in front of patient | 3 | 5 |
| Doctor patient relationship | 5 | 7 |
| GP preference | 4 | 8 |
| NHS policy or contextual standards (international) | 30 | 51 |
| Outcomes (positive) | 58 | 128 |
| Outcomes (negative) | 22 | 28 |
| Patient as delivery method | 2 | 2 |
| Patient harm | 24 | 33 |
| Patient letters | 18 | 34 |
| Patient preference | 37 | 94 |
| Patient recall | 11 | 12 |
| Queries and contact | 10 | 12 |



For peer review only - http://bmjopen.bmj.com/site/about/guidelines.xhtml

For peer review only

| | | |
|---|---|---|
| Understanding | 46 | 88 |

During, ‘reconciliation of data discrepancies’ [19, 20, 24] and ‘adjudication of data’ [19, 20, 24], the data coded within NVivo was used for scanning and comparing data to identify disparities. Adjudicating and situating evidence was important to reconcile discrepancies [19, 20, 24]. We interpreted the data coded within each node and judgements were formed as to which sections of text might be functioning as contexts, mechanisms or outcomes. We then made assessments about what the CMOC might plausibly be for each CMO based on the data within each node. Where relevant, we also drew on data contained within other nodes to build CMOCs. Following this process, a CMOC table was constructed (see *Supplementary file 5)* for consolidation of data.

After table completion, following Pawson’s framework [20], it was important to make sense of the “winners” and “losers”. CMOCs were primarily labelled according to how evidence was reported in the included documents, such as whether the outcomes were described as desirable or beneficial. Where evidence was limited or outcomes were not clearly described or evaluated, the research team interpreted what data were available and formed judgements about these CMOCs based on content expertise in order to generate “positive” and “negative” labels. CMOCs were not limited to one per document or one per patient experience. Thus, multiple outcomes and CMOCs could be annotated for a single experience; this exemplifies the complexity of the intervention under scrutiny.

Notably, there were a greater number of CMOCs relating to positive outcomes than when the intervention does not work.

**Programme theory refinement (step 6): Patient and public involvement**

Review step 6 was to consider stakeholder perspectives to test and refine the PT in light of the synthesised data [20] and to assess whether the PT aligns with real-life experiences [18]. We invited comment from local policy makers and health service commissioners, GPs and a patient and public participation group. Groups were selected according to convenience and accessibility through University links. They were invited to suggest refinements to the PT in an entirely voluntary format, and all comments were anonymised. Formal ethical approval was not required [23] but informed involvement was sought.

## RESULTS

### Document characteristics

The 103 evidence sources were from 16 countries across various continents with most emanating from England (54%), the US (17%), and Australia (7%). Healthcare settings were split between insurance style systems (23%) and publically funded systems (77%), such as the NHS. The date range of the sources was from 1979-2017 and the total number of participants detailed across the research studies was 16,383; this included staff and patient participants but there was not enough detail across all of the studies to quantify the participant type proportions. Most had been published in the 10 years prior to the search: 1970-1979 (1%), 1980-1989 (2%), 1990-1999 (7%), 2000-2009 (40%), and 2010-2017 (50%). The source type was mixed: discussion and opinion pieces (20%), survey-based study (19%), guideline documents (12%), abstracts (7%), review (5%), interview-based study (5%), experimental study (5%), pilot study (5%), randomised controlled trial or



For peer review only - http://bmjopen.bmj.com/site/about/guidelines.xhtml

randomised intervention study (5%), non-randomised intervention study (3%), report document (3%), cohort study (2%), mixed methods (not covered above) (1%) and other e.g. PhD thesis (8%).

The evidence covered a wide range of specialties. Most specified inclusion of adult patients only (over 18 years) but often did not detail the exact patient ages in the write up; a few studies focussed on elderly patients. Information relating to patient demographics e.g. gender, was often not found in the sources and hence these were not summarised. Many sources instead focused on the speciality under consideration in the document and clinical presentations of interest to that speciality e.g. ECGs [32]. Participants who were staff included medical students, doctors of all training grades, nurses, GPs, non-specified hospital staff, and non-clinical staff. However, the majority of documents (66%) either did not provide staff participant details or they were irrelevant e.g. guideline document, no participants. The type of discharge communication varied: direct copies (48%), discharge instructions (13%), pictures (1%), personal discharge packs (1%), personalised letters (13%), information booklets (9%), multiple types of discharge communication (7%), and other (11%). Where the sources came from showed some variation such as Department of Health archive (3%) and conference listing (5%) but the greatest number of sources were from journals (68%).

**Quality and document rigour**

The findings were considered in light of the quality of included documents. During data extraction, documents were quality appraised for *rigour* and evaluated for *relevance* [19, 20]. The concept of *rigour* is defined as 'whether the methods used to generate the relevant data are credible and trustworthy' [18]. *Relevance* and *rigour* were scored on a scale from very low to very high and factors such as document type (e.g. opinion piece or scientific trial paper) were considered. Documents were not excluded solely based on rigour as extracts of documents with a lower quality score may still have valid contributions [20]. The quality of evidence varied, with 53% of sources graded as medium or above for relevance and 80% for rigour. Information relating to setting and context was often limited.

**Context-mechanism-outcome configurations**

The following section provides an overview of theories in the form of a narrative of how patients receiving discharge letters does or does not work, as informed by the evidence reviewed. The sub-headed themes emerged during data interrogation and consolidation although many acted as "nodes" in earlier annotation and coding (see Table 1). Sections contain references to CMOCs, quotations from data texts, and references. Quotations have been chosen which illustrate the described theories and highlight key elements of CMOCs. The full table of 48 CMOCs is found in *Supplementary file 5*.

Evidence relating to some aspects of the PT was limited, particularly in relation to negative outcomes, intervention costs, current clinician views, impact on doctor-patient relationships, personalised patient letters and patients not receiving any intervention. Evidence was also thin in relation to data disparities. Although, context and outcome information was generally well supported, mechanisms were frequently omitted. Where possible, based on the evidence and research team expertise, we inferred reasons for disparities and what the likely mechanism(s) were within any CMOC.

*Patient preference/choice*

Allowing patients to make their own choice for receiving letters may: reduce unnecessary resource strain [33][CMOC14], only take minimal time [9], make patients feel



For peer review only - http://bmjopen.bmj.com/site/about/guidelines.xhtml

more involved in their care [2, 9, 34-38] [CMOC2], increase satisfaction [10, 16, 35, 39-43] [CMOC14, CMOC41, CMOC47] and aid information acceptance [14]:

> ''*I wanted to know as much as possible about what was going on with my body*" [14](p.73)

> "*Sometimes for whatever reason you don't fully take on board what the doctor has told you. I found the letter useful to read over and digest properly what was written*" [2](p.3)

Many patients report that receiving letters is useful [2, 7, 16, 33, 37, 38, 40, 44, 45]. Patients may show friends/family to help them better understand their condition/treatment [14, 16, 35, 37, 43, 46-48]. Patients may use letters as a reference/reminder for the consultation [14, 16, 35, 37, 40, 41][CMOC45]:

> *"My mind went blank after seeing the doctor and the letter reminded me of what had been said"* [37](p.83)

Across a range of specialties and settings, the reported patient preference for receiving copies of their discharge letter is generally high (79%-97%) [7, 8, 14, 35, 36, 41, 44, 45, 49-52]. However, not all patients may find letters helpful [33, 43] or necessary [7, 33-35, 40, 43, 53], and some may not want to be reminded of their diagnosis [35], which could decrease satisfaction, and generate queries if these patients were sent letters without a choice [CMOC40]. Hence, several studies argue in favour of respecting patient choice and suggest the patient's right to "opt out" needs to be addressed [7, 14, 16, 40, 50, 53-55] [CMOC41]. In situations where the patient is not offered a choice, such as third party information or risk of harm [2], the clinician should be able to justify this decision [56]. In relation to sensitive information or social diseases, patients generally do not object to this being included in the letter as long as it has 'some relevance' [56].

*Comprehension, queries and recall*

There was considerable evidence, particularly from patient viewpoints, to support the view that the majority of patients may understand their letters [7, 8, 14, 15, 33-37, 44-46, 56, 57] and hence find the letter beneficial and helpful [33, 44, 58] [CMOC7, CMOC39, CMOC44]. Moreover, a letter copy which is understood can reassure patients they are being listened to [43, 44, 59] [CMOC34, CMOC48]. Patient understanding of discharge instructions may increase their knowledge [43] and this might reduce unnecessary or avoidable hospital readmissions [60-64] and reduce patient anxiety [65] thereby supporting patient wellbeing [16, 51, 56] [CMOC39]:

> *"I found the letter very comforting and reassuring"* [66](p.58)

Although there may be a risk that patients receiving letters is associated with an increase in queries to seek clarification about what has been communicated [55], several sources indicated that this occurs to a minimal extent [8, 34, 44, 52, 56] [CMOC29]. Examples of patients not understanding letters were sometimes described as a "small proportion" [33] or low percentage '7%' [35].

If patients are provided verbal information only, they may fail to retain the information [37, 41] [CMOC18] which can decrease recall and adherence [CMOC21]:

> *"By the time I have got home, I have forgotten half of what was said in clinic."* [44](p.255)

Due to this, combining written and verbal information [67] may improve patient understanding [37, 68-71] [CMOC15, CMOC18], increase patient's involvement in their care [16, 37] and compliance [2, 17, 45, 59, 72, 73] [CMOC11, CMOC43], and improve recall [10, 14, 15, 34, 41, 45, 69, 74, 75] [CMOC5, CMOC15].



For peer review only - http://bmjopen.bmj.com/site/about/guidelines.xhtml

Contexts which may increase likelihood of triggering patient understanding include: letter language translation [39, 76, 77], writing the letter at a 5th or 6th grade reading age level [39, 69, 78, 79] [CMOC12], use of glossary [16, 80], pictures, pictographs or equivalent [69, 80-82] (particularly for low literacy or illiterate patients) [CMOC17], lay explanations for 'technical terms' [16, 56] and writing in plain English with minimal jargon/abbreviations [2, 36, 40, 56, 69, 79, 83-85] [CMOC12].

Two sources with the same lead author suggested training medical students on writing patient letters can help produce letters that are more meaningful to patients [66, 86] [CMOC13]. However, the evidence around training in relation to the intervention was limited and needs further research.

*Personalised or patient-directed discharge letters*

Producing a letter which is comprehensible and useful to both GPs and patients has been recognised to be an issue [17, 48, 78, 87]. Patient-directed or personalised patient letters have been proposed [CMOC24, CMOC36]. Patients often rate these letters positively [6, 43, 88] and this may heighten satisfaction [43], and improve understanding [6, 89]:

> *"Simplifying written communication has also been shown to improve patient comprehension."* [6](p.855)

However, personalised letters have the potential to lead to resource consumption [45] [CMOC25], staff time depletion [33, 45, 89] and patient anxiety that they have been given different information to their GP [89] [CMOC26]. For these reasons, further research which weighs the benefits of personalised patient letters against the drawbacks and costs is needed.

*Patient to deliver letter*

The context of patients delivering letters seems to have few reported positive outcomes. Posting and electronic transferral of letters may be preferable as:

> *"It is not considered good practice to send the discharge summary home with the patient as there is no guarantee that the information will be passed on to the general practitioner"* [90](p.7)[CMOC31].

*Dictating letters in front of patients*

Evidence for this concept was somewhat thin. One study suggested that dictating letters in front of patients can make patients feel less in need of a copy of the letter [8]. Another paper suggested this practice may also provide a context that triggers patients to challenge inaccuracies, improving letter quality [91] [CMOC22, CMOC30]:

> "*The content of letters to GPs is sometimes incorrect and this may be remedied by dictating the letter in front of the patient."* [91]

*Confidentiality*

There are concerns and legal implications surrounding potential confidentiality breaches associated with patients receiving letters, particularly when they are sent out in the post [36, 41, 49, 51, 54, 87, 92]. One recent paper [49] (2013), which looked at confidentiality, continued to stress risks around postal communication and the importance of secure information transfer:

> "*There is a substantial risk of breaching patient confidentiality when distributing correspondence by post. A well-designed security arrangement is therefore required to ensure the safety of confidential information."* [49](p.35)



For peer review only - http://bmjopen.bmj.com/site/about/guidelines.xhtml

Some documents [2, 36, 49, 54, 56] suggested ways to reduce potential risk of confidentiality breach through communication platforms and the processes involved in sending letters e.g. verifying patient contact details before sending letter [36, 59] [CMOC3, CMOC27, CMOC28].

*Patient harm*

Patient anxiety or "harm" in general are often cited as reasons for clinicians not wanting to copy letters, particularly in "bad news" settings [14-17, 37, 45, 48, 57, 93] [CMOC6]. Letter inaccuracies can cause concern leaving patients feeling confused or anxious [33] [CMOC19]. Nevertheless, the letter can reassure the patients their problems are being handled [51][CMOC46] and initial anxiety can settle or be nullified by the usefulness of the letter [7, 42-45, 56, 86, 92] [CMOC37]. Moreover, one study [40] published in the *Lancet* in 1991 suggested patient letters in "bad news" settings may be more useful than "good news":

> "*Patients who had received bad news found the letter significantly more useful in helping them to understand and remember what they had been told during the consultation than did patients receiving good news… almost half the patients receiving bad news found their letter distressing to some extent; however, with 1 exception, all patients were pleased to have received it."* [40] (Pp.924-925)

Although the above paper was published in 1991, we found no recent evidence or system changes to dispute the notion that "bad news" letters may be of particular use to the patient. Hence, despite risk of initial "harm", "bad news" letters should perhaps not be avoided.

Practical and feasible suggestions were found in some documents for minimising harm or anxiety: not copying letters with information not previously disclosed to the patient [2, 3, 14, 56] [CMOC38], abstain from use of value judgements e.g. pleasant lady [37] [CMOC12], potentially avoid or carefully consider copying letters where there are 'problems of privacy at home' and/or where the patient lacks capacity [2] [CMOC20], and checking the patient consents to a letter [56] [CMOC41].

*Clinician views*

GP and hospital clinician views were described both as broadly in favour [9, 34, 48, 59, 89, 94, 95] [CMOC5, CMOC16] and not in favour of patients receiving written discharge communication across a range of specialities [10, 11, 16, 34, 36, 37, 46, 48, 51, 89, 95, 96] [CMOC6, CMOC35]. The response section [9, 11, 87] to a BMJ article [97] (2008) on patient letters demonstrates the clinician view dichotomy as practitioners argue for and against patients receiving letters:

> "*My colleagues and I have had to explain to alarmed and bewildered patients who have received copies of their correspondence the meaning of phrases…"* [87](p.1369)

> "*The purposes of clinic letters are to communicate with general practitioners and keep a legible record in the notes of what is happening and what might happen. It is written in medical speak, and it is fantasy to suggest that letters written like that will ever be meaningful, without further explanation, to most patients.*" [11] (p.1369)

> "*Generally, doctors who are sceptical about copying letters to patients seem not to have tried it, whereas those who send copies routinely are enthusiastic."* [9](p.1370)

Practitioner perceived benefits found in the sources [CMOC5] included: improved patient understanding [48, 89], increased transparency [46] [CMOC33], improved trust/doctor-patient relationship [9, 48, 89], dispelling fears of 'secretive relationships' between clinicians [48] and heightened sense of patient importance [48]. In addition, the patients' right to view the information was noted [89] [CMOC7, CMOC16]. A common practitioner concern of the intervention across specialties was letter comprehensibility and patient understanding [11, 16,



For peer review only - http://bmjopen.bmj.com/site/about/guidelines.xhtml

[34, 36, 37, 46, 48, 51, 87, 89, 98, 99] [CMOC6]. Other concerns included: cost of additional materials/staff time [17, 33, 34, 49, 51, 87, 89] [CMOC23, CMOC32], patient anxiety [16, 17, 36, 37, 48, 51, 89, 96] [CMOC6, CMOC19], increased patient queries [17, 34] [CMOC29], potential confidentiality breaches [48] [CMOC6, CMOC27], and that letters would need to be oversimplified [16, 17, 48, 89, 99, 100]. An attitudinal issue found in two oncology documents [46, 89] published 17 years' apart was the view that letters are tools to be used between doctors only [CMOC6]. Additionally, juniors can learn from and mimic superiors and also not send letters to patients [96].

Confusion around 'letter comprehensibility' and lack of 'patient understanding' were the commonest clinician reservations relating to the intervention [11, 16, 34, 36, 37, 46, 48, 51, 87, 89, 98, 99]. However, as covered in the *comprehension* section, patients are often reported as *understanding* their letters [7, 8, 14, 15, 33-37, 44-46, 56, 57] and furthermore they tend to express strong preference for receiving such letters [7, 8, 14, 35, 36, 41, 44, 45, 49-52]. Thus, it may be inferred from the evidence that patient understanding of letters is possibly higher than clinicians' perceive [34, 45, 57]. The following from a recent (2016) abstract [46] concisely summarises an example of patient and clinician view disparity:

> "*While some oncologists assess the copy letters as inappropriate for supplemental patient-oncologist-communication, breast cancer patients regard this tool as predominantly gainful. Oncologists appear to stick to their traditional perspective which perceives the copy letter mainly as a communication tool from doctor to doctor.*" [46] (p.185)

Notably, much of the evidence reporting clinician views was published from 2002-2008 and current evidence on clinician perspectives remains limited. Moreover, although sources occasionally referred to conflicting clinician views, information on *why* attitudes differ was thin. Overall, better understanding of current clinician views on copying discharge letters to patients is required. Further research should address reasons behind different viewpoints to include patients and practitioners.

*Cost and resources*

The estimated costs associated with the intervention varied [16] but this must be considered in the context that included documents spanned a wide time range and thus factors such as inflation need to be considered. In addition, robust health economic analyses were not found in the included sources. Documents [16, 17, 33-35, 37, 41, 44, 50-52, 54-56, 87, 91, 101] referred to "cost" or financial implications [CMOC25] of sending letters in different ways such as use of consumables [17, 33, 34, 50-52, 55, 101][CMOC10], and secretarial [16, 17, 33, 34, 37, 44, 51, 55, 56, 101] [CMOC10] and clinician time required [17, 33]. A few sources [2, 17, 35, 37, 45, 56, 102], including guideline documents and research papers, suggested that benefits were such that associated costs were minimal, or even reduced by patients being more informed from receiving discharging communication [CMOC7, CMOC25, CMOC42]. However, as many of these views were based on personal comment or studies with weak methodologies, the true cost consequences remain unknown.

*Autonomy*

One source suggested that when patients are not given letters, they may feel less involved in their care, resulting in reduced patient autonomy [42] [CMOC1, CMOC6]:

> *"…to refuse to provide such information if this is the patient's wish is to deny their autonomy."* [42] (p.388)

Conversely, some evidence [16, 42, 103] was found that providing patients with written discharge letters is their "right" [3, 54], may create a sense of involvement, and increase patient autonomy and satisfaction [CMOC2, CMOC4, CMOC5, CMOC8, CMOC14].

*Doctor-patient relationships*



For peer review only - http://bmjopen.bmj.com/site/about/guidelines.xhtml

Few documents [2, 9, 14, 16, 48, 73, 89] were found which considered the intervention in terms of the doctor-patient relationship. However, much of the limited evidence that was found indicated that patients receiving letters has the potential to improve communication, trust and the doctor-patient relationship [CMOC9] [2, 14, 16, 48, 73].

## Stakeholder perspectives

As detailed in step 6, the final review step was to refine the programme theory through stakeholder perspectives. Three groups were consulted: local commissioners, GPs and service-users/patients. Stakeholder involvement took the form of group discussions and email correspondence. As the PT was continually being developed throughout the review process, stakeholders commented on the most recently developed PT at the time of their involvement. Groups were relatively small; due to feasibility it was not possible to achieve diverse and representative group samples.

Group discussions were centred on the programme theory; members were encouraged to critique and feedback on the PT diagram. This included concepts not covered or explored in detail in the PT diagram such as: the importance of comprehensible language and terminology, difficulty and problems retaining verbal information only e.g. following use of anaesthesia, patient choice of receiving letters, illegibility of handwritten discharge communication, critical context of prior patient communication of a high quality to increase likelihood of understanding discharge letters, issues around personalised patient letters considering NHS resource availability, and concerns around writing a letter which meets the needs of both GP and patient. The commissioner and GP representatives emphasised the importance of patient safety and that this should be central to best practice recommendations. In addition, the patient group reported reading a letter about themselves written in third person was peculiar. The patient group felt patient letters were very important for patients taking responsibility for their health in line with the NHS promotion of patient-centred and patient-led care.

Several different members across the various groups commented that in practice, patients do not always receive their letters, despite this process being recognised as best practice. Recommendations were suggested to rectify this by the commissioner members to include: clinicians should assume when writing letters that they could be made available to the patient, early clinician and student training in good letter writing and record keeping, and that hospitals should support the initiative e.g. quality improvement activities and audits.

## Cycling of review steps

As a realist review is an iterative process, steps may be repeated. As described in step two, new publications were followed and consulted for evidence but provided no new or conflicting PT knowledge. Thus, it was deemed that "theoretical saturation" [19, 21] in accordance with Pawson's realist review methodology [19-22] was attained and no further searching or step cycling was required.

## Resultant programme theory

The PT was systematically updated to produce a resultant PT following review steps 1-6 (Figure 4). This still shows two main channels for CMOCs: patient copies of letters and patient personalised letters. There remained limited CMOCs for where patients do not receive letters, due to the paucity of evidence available. Contexts for when the patient does receive their letter(s) were condensed into an aligned grouping of five key contexts for when



For peer review only - http://bmjopen.bmj.com/site/about/guidelines.xhtml

the intervention may be theorised to work and four key contexts for when the intervention may be theorised not to work. The feasibility of providing a personal patient letter was updated on the PT; findings from both stakeholder feedback and data synthesis suggested personalised letters may currently be more feasible in private or insurance-based healthcare settings than in the NHS. In addition, the box of contextual influences was deleted, and the points integrated into the overall diagram. Patient outcomes throughout the PT were simplified and clarified (e.g. the outcomes such as ‘empowered patients’ and ‘reduce patient anxiety’ were simplified to the outcome ‘improved patient well-being’).

In addition, CMOCs have been “grouped” where overlap was apparent, for example, all resources are labelled simply as “resources” as data often concurrently referred to financial, administrative and clinician time resources. “Patient choice” emerges as a key influencer to the success (or not) of the intervention. Notably, there are a range of contexts, mechanisms and outcomes for when the intervention is theorised to “work” (e.g. positive outcome=improve doctor-patient relationship), and when it does “not work” (e.g. negative outcome=harm to patient). It is also evident that contexts can be used in combination to increase the likelihood of beneficial outcomes; this is indicated through wide arrows to show multiple linkages and amalgamations (e.g. a letter could contain no value judgements of patient and be written in simple plain English). Notably, the resultant PT has a higher incidence of CMOCs for when the intervention “does work”; this is considered further in the discussion.



For peer review only - http://bmjopen.bmj.com/site/about/guidelines.xhtml

*Figure 4 Resultant Programme Theory*

For peer review only



For peer review only - http://bmjopen.bmj.com/site/about/guidelines.xhtml

## DISCUSSION

### Statement of principal findings

This review of 103 sources summarises and expands upon existing evidence by moving beyond "benefits" and "drawbacks" of patients receiving letters alone, and considering contexts of *when* as well as *how* the intervention works. Although the review focuses on the UK health system, our use of realist review has enabled identification of findings that may be transferable to other healthcare settings.

RQ1 asked about positive and negative outcomes of the intervention. Positive outcomes include: increased patient satisfaction (10, 16, 35, 39-43), improved doctor-patient relationship and trust (14, 16), heightened patient knowledge (43), improved letter and record quality (2, 56, 91), and reduced anxiety (65). Negative outcomes include patient queries (55), confusion (48, 51), and anxiety (15, 43).

RQ2 enquired after the important contexts for triggering these outcomes. Important contexts for positive outcomes include: letters written in plain English with minimal abbreviations (56), lay explanations or simplified terms in brackets for medical jargon (16, 56, 83) e.g. myocardial infarction (heart attack), written information provided alongside verbal explanation (67), no new information in letter (2, 14) or value judgements (37), letter translation (39, 76, 77) where relevant, training clinicians on letter writing practice (2, 66, 84, 86), use of pictures and glossaries where relevant (16, 69, 80-82), letters only given to patients who choose to have them (16, 33, 56), and where there is no identified risk of harm (2) or confidentiality breach (56). Important contexts for negative outcomes include: patient choice not acknowledged (56), only verbal information provided (44), letter involving terms and a style that is too advanced for patient to comprehend (78), and letter sent without verifying patient contact details (36, 54, 59).

This review has produced two key findings, which are important but not wholly surprising. The first is that the reviewed evidence indicates that patients value their discharge letter and their understanding of them is possibly greater than clinicians perceive (34, 45, 57). However, reasons behind patient and clinician perceived comprehension discrepancies were unclear. It is important to situate the first finding in terms of the study exclusion criteria and participant diversity across the evidence reviewed, for example, it is likely that patients who participate in research on this topic have a greater level of interest and literacy than those who did not participate. One or a number of demographic groups not involved in the studies, either by choice not to participate or by exclusion, may have accounted for a portion of those who clinicians perceive to have low understanding. Thus, evidence for low patient understanding was limited, and this requires further research. The second key finding is that in a number of contexts, patients expressed preference for receiving correspondence (7, 8, 14, 35, 36, 41, 44, 45, 49-52). Patients can continue to use the letter(s) to refer to beyond discharge (16, 35, 37), as a medication list reminder, and to share with friends/relatives as desired (15, 35, 37, 89). Nevertheless, patient *choice* should still be acknowledged as the review did find evidence that not *all* patients want their letters; a practical way of addressing this would be to check with the patient that they want a letter in the first instance (7, 16, 54, 56).

Systems for monitoring patient letters (e.g. the Newcastle Trust Policy for auditing and sharing letters with patients (56)) seems prudent moving forward. This is of particular relevance in the NHS given that guidelines for copying letters have been widely available since 2003 (2) and yet in practice, many patients do not receive letters (16, 49). Given the wider context of a drive for patient-led care and patient-centred communication and decision-making (17, 42, 104), this review is timely and relevant. The review findings have the potential to influence policy and improve practice. The results demonstrate how care can be improved through patient choice and good quality letter provision. However, current clinician views (34, 36, 37, 45, 46, 57, 89) and hierarchical mimicking of practices of seniors (96) pose a barrier to implementation and need addressing.



For peer review only - http://bmjopen.bmj.com/site/about/guidelines.xhtml

## Review limitations

For this review we followed the RAMESES quality and publication standards for realist reviews [105, 106]. Quality assessment and analysis is to a degree dependent on reviewer skills and reflexivity [107, 108]. Furthermore, analysis and inferences were 'subjective and interpretative' [109, 110]. However, because the steps we have taken for this review are transparent, other review teams can see and make judgements on result plausibility.

Due to lack of time, it was not possible to involve all stakeholder groups who may be connected with discharge communication. Nonetheless, the review had a specific focus on "receiving" discharge communication and thus stakeholders were targeted who were closely associated or involved in policy of discharge letter receipt.

The resultant PT is limited by the quality and content of evidence reviewed. Some of the evidence found in sources was markedly thin, particularly in relation to costing information, recent clinician viewpoints, personalised letter copies, and influence on the doctor-patient relationship. Furthermore, there were a greater number of CMOCs relating to positive outcomes than negative outcomes, that is, when the intervention *does* work than *doesn't.* This may be rationalised by publication bias towards positive findings. Additionally, the binary distinctions between positive and negative outcomes, that is, when the intervention does and does not work, may have imposed oversimplified CMOC labels. "Positive" and "negative" labels were based on evidence presented in the documents reviewed; at times a degree of subjectivity was involved in this process. Although these binary labels (positive/negative) may have oversimplified some CMOCs, we felt the usefulness of clear distinctions between when the intervention was interpreted to "work" (and not) outweighed the drawbacks of this method.

CMOCs for patients *not* receiving letters (nil intervention) were thin. Consequently, these evidence limitations constrained the detail available in the resultant PT in these areas. Additionally, not all mechanisms could be inferred from the data resulting in some visible mechanism "gaps" within the CMOC table (*supplementary file 5*).

The review and PT are not exhaustive but this is not the intention of a realist review [111]. Given time and resource constraints, the review was limited to adult patients who had been discharged from general hospital settings, and other patient groups were excluded. Furthermore, the PT is limited by the representativeness and diversity of the patient groups within the sources reviewed. Much of the evidence was drawn from small scale studies conducted in single settings, and even within these there is likely to have been participation bias which will have resulted in the views of ethnic minorities, patients lacking literacy and other hard to reach groups being under-represented.

## Suggestions for future research

The PT offers a useful starting point for future research and should be useful and practicable for informing policy and guidelines. Further research is needed to explore the relevance of the PT to groups, such as children and adults being discharged from mental health services, who were excluded from this review and to those, such as hard-to-reach groups, who may have been under-represented in the evidence included in the review. There is also a need for research to define the cost benefits of copying patients into discharge letters in order that the importance of this topic and the consequences of poor practice are recognised by policymakers, managers and professional bodies. Potential barriers such as clinician views and the current limited available clinician training on letter writing should be addressed; research and evaluation is needed to inform how this can be effectively achieved. Since patient and clinician views were sometimes conflicting, a study which parallels both views alongside the same patient cases to understand reasons for any



For peer review only - http://bmjopen.bmj.com/site/about/guidelines.xhtml

discrepancies would be useful and may provide valuable insights. This is the topic of a PhD that is currently being undertaken by the lead author, and will be reported in due course.

## CONCLUSION

The resultant PT forms a basis for explaining how, when, why and for whom this intervention does and does not work. The resultant PT makes suggestions for how best practice of patients receiving discharge letters may be improved to enhance the provision of patient-centred care. Evidence for some aspects of the PT was rather limited, indicating a need for more research.

The key findings are that the value patients place on discharge letters and their understanding of the letters' content is possibly greater than clinicians' perceive, patient choice is instrumental to increasing the likelihood of desired outcomes, and that clinician views may act as a barrier to wider practice implementation. This could be addressed through clinician training and organisational initiatives which guide, mandate, and monitor the intervention. Without such organisational support, it is unlikely that current processes will be consistently improved given the barriers identified in the review.

In conclusion, this review describes how the intervention of patients receiving their discharge letters may *work* to increase the likelihood of positive effects and reduce potential negative effects.

**Author contributions:** KW was the lead reviewer. EM completed the second reviewer tasks. All authors contributed toward the programme theory through discussions. KW is responsible for the design and drafting of the initial manuscript. GW, ES, SS and JD critically reviewed and edited the final manuscript. All authors read and approved the final manuscript.

**Acknowledgements:** Samantha Johnson, our information specialist, for critically appraising the search strategy. We would also like to thank all stakeholders involved in the review for improving the quality and relevance of the resultant theory.

**Funding statement:** This work is supported by the Economic and Social Research Council (ESRC) and Clinical Commissioning Groups (CCGs) of Coventry & Rugby and South Warwickshire. Funding for the open access charges for publication of this review was provided by the RCUK fund.

**Data sharing statement:** This review presents previously published and publically available data. Please refer to the reference list (below and in supplementary file 4) and their authors for these research data.

**Competing interests:** None known

## References

1.NHS England. Standards for the Communication of Patient Diagnostic Test Results on Discharge from Hospital. 2016 https://improvement.nhs.uk/uploads/documents/discharge-standards-march-16.pdf.
2.*Department of Health*. Copying letters to patients: good practice guidelines. 2003 http://webarchive.nationalarchives.gov.uk/.
3.*Department of Health*. The NHS Plan: A Plan for Investment a Plan for Reform. In: HMSO, editor. London2000. Available from http://webarchive.nationalarchives.gov.uk.
4.Harris E., Rob P., Underwood J., Knapp P., Astin F. Should patients still be copied into their letters? A rapid review. *Patient Education & Counseling*. 2018;101(12):2065-82.



For peer review only - http://bmjopen.bmj.com/site/about/guidelines.xhtml

5.The Academy of medical Royal Colleges. Please, write to me: Writing outpatient clinic letters to patients. *AOMRC*. 2018 https://www.aomrc.org.uk/reports-guidance/please-write-to-me-writing-outpatient-clinic-letters-to-patients-guidance/.
6.Lin R., Gallagher R., Spinaze M., Najoumian H., Dennis C., Clifton-Bligh R., Tofler G. Effect of a patient-directed discharge letter on patient understanding of their hospitalisation. *Internal medicine journal*. 2014;44(9):851-7.
7.O'Driscoll B.R., Koch J., Paschalides C. Copying letters to patients: Most patients want copies of letters from outpatient clinics and find them useful. *Bmj*. 2003;327(7412):451.
8.Pothier D.D., Nakivell P., Hall C.E. What do patients think about being copied into their GP letters? *Annals of the Royal College of Surgeons of England*. 2007;89(7):718-21.
9.Shee C.D. Try it and see. *Bmj*. 2008;337(a2786):p.1370.
10.Saunders N.C., Georgalas C., Blaney S.P., Dixon H., Topham J.H. Does receiving a copy of correspondence improve patients' satisfaction with their out-patient consultation? *Journal of Laryngology & Otology*. 2003;117(2):126-9.
11.Main J. Copying in or copping out? *Bmj*. 2008;337(a2688):p.1369.
12.Marzanski M., Musunuri P., Coupe T. Copying letters to patients: A study of patient's views. *Psychiatric Bulletin*. 2005;29(2):56-8.
13.Holdsworth T. Letters as a record of clinic appointments or for Communication. *Bmj*. 2008;337:a2324.
14.Fenton C., Al-Ani A., Trinh A., Srinivasan A., Marion K., Hebbard G. Impact of providing patients with copies of their medical correspondence: a randomised controlled study. *Internal medicine journal*. 2017;47(1):68-75.
15.Hallowell N. Providing letters to patients. Patients find summary letters useful. *Bmj*. 1998;316(7147):1830.
16.Baxter S., Farrell K., Brown C., Clarke J., Davies H. Where have all the copy letters gone? A review of current practice in professional-patient correspondence. *Patient Education & Counseling*. 2008;71(2):259-64.
17.Boaden R., Harris C. Copying letters to patients—will it happen? : Oxford University Press; 2005. Available from https://academic.oup.com/fampra/article/22/2/141/522310
18.Brennan N., Bryce M., Pearson M., Wong G., Cooper C., Archer J. Understanding how appraisal of doctors produces its effects: a realist review protocol. *BMJ Open*. 2014;4(6).
19.Pawson R., Greenhalgh T., Harvey G., Walshe K. Realist review--a new method of systematic review designed for complex policy interventions. *J Health Serv Res Policy*. 2005;10 Suppl 1:21-34.
20.Pawson R., Greenhalgh, T., Harvey, G. & Walshe, K. . Realist synthesis: an introduction.' *ESRC Research Methods Programme*. 2004 Available at: https://goo.gl/1Rz2Ry.
21.Pawson R. Digging for nuggets: how 'bad' research can yield 'good' evidence. *International Journal of Social Research Methodology*. 2006;9(2):127-42.
22.Pawson R., editor Middle range theory and program theory evaluation: From provenance to practice. Transaction Publishers; 2010.
23.Weetman K., Wong G., Scott E., Schnurr S., Dale J. Improving best practice for patients receiving hospital discharge letters: a realist review protocol. *BMJ Open*. 2017;7(11).
24.Wong G., Brennan N., Mattick K., Pearson M., Briscoe S., Papoutsi C. Interventions to improve antimicrobial prescribing of doctors in training: the IMPACT (IMProving Antimicrobial presCribing of doctors in Training) realist review. *BMJ Open*. 2015;5(10).
25.Ford J.A., Wong G., Jones A.P., Steel N. Access to primary care for socioeconomically disadvantaged older people in rural areas: a realist review. *BMJ Open*. 2016;6(5):e010652.
26.Centre for Reviews and Dissemination. Systematic Reviews: CRD's guidance for undertaking reviews in health care. 2008 https://www.york.ac.uk/media/crd/Systematic_Reviews.pdf.



For peer review only - http://bmjopen.bmj.com/site/about/guidelines.xhtml

27.Moher D., Liberati A., Tetzlaff J., Altman D.G. Preferred reporting items for systematic reviews and meta-analyses: the PRISMA statement. *Bmj*. 2009;339.
28.Wiese A., Kilty C., Bergin C., Flood P., Fu N., Horgan M., Higgins A., Maher B., O'Kane G., Prihodova L., Slattery D., Bennett D. Protocol for a realist review of workplace learning in postgraduate medical education and training. *Systematic Reviews*. 2017;6:10.
29.Mills S.L., Pumarino J., Clark N., Carroll S., Dennis S., Koehn S., Yu T., Davis C., Fong M. Understanding how self-management interventions work for disadvantaged populations living with chronic conditions: protocol for a realist synthesis. *BMJ Open*. 2014;4(7):e005822.
30.Pawson R. *Evidence-based policy: a realist perspective*: Sage; 2006.
31.Brennan N., Bryce M., Pearson M., Wong G., Cooper C., Archer J. Towards an understanding of how appraisal of doctors produces its effects: a realist review. *Med Educ*. 2017;51(10):1002-13.
32.Ackermann S., Heierle A., Bingisser M.B., Hertwig R., Padiyath R., Nickel C.H., Langewitz W., Bingisser R. Discharge Communication in Patients Presenting to the Emergency Department With Chest Pain: Defining the Ideal Content. *Health Communication*. 2016;31(5):557-65.
33.Pinder E., Jefferys S., Loeffler M. Patient Satisfaction: Receiving a copy of the GP letter following fracture or elective orthopaedic clinic. *BMJ Qual Improv Rep*. 2013;2(2).
34.Brockbank K. Copying patient letters - Making it work. *Clinical Governance*. 2005;10(3):231-40.
35.Krishna Y., Damato B.E. Patient attitudes to receiving copies of outpatient clinic letters from the ocular oncologist to the referring ophthalmologist and GP. *Eye (Lond)*. 2005;19(11):1200-4.
36.Treacy K., Elborn J.S., Rendall J., Bradley J.M. Copying letters to patients with cystic fibrosis (CF): letter content and patient perceptions of benefit. *J Cyst Fibros*. 2008;7(6):511-4.
37.Tomkins C.S., Braid J.J., Williams H.C. Do dermatology outpatients value a copy of the letter sent to their general practitioner? In what way and at what cost? *Clinical & Experimental Dermatology*. 2004;29(1):81-6.
38.Perkins P., Jordan A., Prentice W., Regnard C. Copying letters to patients: a survey of patients and GPs views. *Palliative medicine*. 2007;21(4):355-6.
39.Advancing effective communication, cultural competence, and patient- and family-centered care : a roadmap for hospitals. *Joint Commission*. 2014 http://www.jointcommission.org/assets/1/6/aroadmapforhospitalsfinalversion727.pdf.
40.Damian D., Tattersall M.H. Letters to patients: improving communication in cancer care. *Lancet*. 1991;338(8772):923-5.
41.Antoniou A., Saunders M., Bourner R., Crouch L. would you like to see yours? *The Bulletin of the Royal College of Surgeons of England*. 2007;89(2):62-4.
42.Chantler C., Johnson J. Patients should receive copies of letters and summaries. *Bmj*. 2002;325(7360):388.
43.O'Reilly M., Cahill M.R., Perry I.J. Writing to patients: a randomised controlled trial. *Clinical Medicine*. 2006;6(2):178-82.
44.Nixon J., Courtney P. Copying clinic letters to patients. *Rheumatology*. 2005;44(2):255-6.
45.Brodie T., Lewis D. A survey of patient views on receiving vascular outpatient letters. *European Journal of Vascular and Endovascular Surgery*. 2010;39(1):5-10.
46.Baumann W., Schussler L., Bertram M., Benser J., Kumpers S., Hermes-Moll K. Oncologists' letters for breast cancer patients. *Oncology Research and Treatment*. 2016;39:184-5.
47.Sandler D.A., Heaton C., Garner S.T., Mitchell J.R. Patients' and general practitioners' satisfaction with information given on discharge from hospital: audit of a new information card. *Bmj*. 1989;299(6714):1511-3.
48.O'Reilly M., Cahill M., Perry I.J. Writing to patients: 'putting the patient in the picture'. *Ir Med J*. 2005;98(2):58-60.



For peer review only - http://bmjopen.bmj.com/site/about/guidelines.xhtml

49.Somov P., Madden T., Wong K., Hamm R. Security Concerns About Copying Clinical Letters to Patients. *The Bulletin of the Royal College of Surgeons of England*. 2013;95(1):33-4.
50.Rao M., Fogarty P. What did the doctor say? *Journal of Obstetrics & Gynaecology*. 2007;27(5):479-80.
51.Jelley D., van Zwanenberg T., Walker C. Copying letters to patients : Concerns of clinicians and patients need to be addressed first. *Bmj*. 2002;325(7376):1359.
52.Sharma D., O'Brien S., Hardy K. Copying letters to patients: What patients think - A questionnaire survey. *Clinician in Management*. 2007;15(2):75-8.
53.Lepping P., Paravastu S.C., Turner J., Billings P., Minchom P. Copying GP letters to patients: a comprehensive study across four different departments in a district general hospital. *Health informatics journal*. 2010;16(1):58-62.
54.Singh S., Budeda B., Housden P. Do patients want copies of their GP letters?–our experience with 7250 patients. *International journal of clinical practice*. 2007;61(8):1407-9.
55.Liapi A., Robb P.J., Akthar A. Copying clinic letters to patients: a survey of patient attitudes. *The Journal of Laryngology & Otology*. 2006;121(6):588-91.
56.The Newcastle upon Tyne Hospitals NHS Foundation Trust. The Newcastle upon Tyne Hospitals NHS Foundation Trust: Sharing Letters with Patients Policy. 2013.
57.Dooher P., Syed A., Liu J., Chopra A., Bradpiece H., Jenkins S., Patel A. Copying letter to patients-distress or satisfaction? *European Journal of Cancer*. 2012;48:S151.
58.Bench S., Day T., Griffiths P. Effectiveness of critical care discharge information in supporting early recovery from critical illness. *Critical Care Nurse*. 2013;33(3):41-52.
59.Smith P.E.M. Letters to patients: sending the right message. *Bmj*. 2002;324(7338):685.
60.Regalbuto R., Maurer M.S., Chapel D., Mendez J., Shaffer J.A. Joint Commission requirements for discharge instructions in patients with heart failure: is understanding important for preventing readmissions? *Journal of Cardiac Failure*. 2014;20(9):641-9.
61.Saidinejad M., Zorc J. Mobile and web-based education: delivering emergency department discharge and aftercare instructions. *Pediatric Emergency Care*. 2014;30(3):211-6.
62.Lorenzati B., Quaranta C., Perotto M., Tartaglino B., Lauria G. Discharge communication is an important underestimated problem in emergency department. *Internal & Emergency Medicine*. 2016;11(1):157-8.
63.HPOE: A Compendium of Implementation Guides 2011. 2011 http://www.hpoe.org/Reports-HPOE/hpoe_compendium_2011.pdf.
64.Cannaby A.-M. *Improving the process of hospital discharge for medical patients* [Ph.D.]. Ann Arbor: University of Leicester (United Kingdom); 2003.
65.Ackermann S., Bingisser M.B., Heierle A., Langewitz W., Hertwig R., Bingisser R. Discharge communication in the emergency department: physicians underestimate the time needed. *Swiss Medical Weekly*. 2012;142:w13588.
66.Mrduljas-Djujic N., Pavlicevic I., Marusic A., Marusic M. Students letters to patients as a part of education in family medicine. *Acta Medica Academica*. 2012;41(1):52-8.
67.Generic Standards. 2002 http://www.healthcareimprovementscotland.org/his/idoc.ashx?docid=ea8d290a-9504-4c21-a889-fc479d530a51&version=-1.
68.Exploring patient participation in reducing health-care-related safety risks. 2013 http://www.euro.who.int/__data/assets/pdf_file/0010/185779/e96814.pdf.
69.Taylor D.M., Cameron P.A. Discharge instructions for emergency department patients: What should we provide? *Journal of Accident and Emergency Medicine*. 2000;17(2):86-90.
70.Carol Lim K.K., Chan S.K., Chew E.L., Anita Lim A.F., Sararaks S., Ainul H., Roslinah A., Tan L.S., Low L.L., Azman A.B., Maimunah A.H. Handoff communication - Let's do it right. *Medical Journal of Malaysia*. 2010;65:8.
71.Reilly M.M. Let's set the record straight: preparing the discharge summary and the patient's instruction sheet. *Nursing*. 1979;9(1):56-61.



For peer review only - http://bmjopen.bmj.com/site/about/guidelines.xhtml

72. Davies J.M., Batuyong E., Lupichuk S.M., Hilsden R., Eliasziw M., Easaw J.C. Cohort study evaluating the impact of a discharge letter (DL) compared with usual care on adherence to surveillance following treatment for stage II/III colorectal cancer (CRC). *Journal of Clinical Oncology Conference*. 2012;30(4 SUPPL. 1).
73. Making time in general practice : freeing GP capacity by reducing bureaucracy and avoidable consultations, managing the interface with hospitals and exploring new ways of working. *NHS Alliance*. 2015 http://www.nhsalliance.org/wp-content/uploads/2015/10/Making-Time-in-General-Practice-FULL-REPORT-01-10-15.pdf.
74. Hoek A.E., De Ridder M.A., Bayliss A., Patka P., Rood P.P. Effective strategy for improving instructions for analgesic use in the emergency department. *European Journal of Emergency Medicine*. 2013;20(3):210-3.
75. Sandler D.A., Mitchell J.R., Fellows A., Garner S.T. Is an information booklet for patients leaving hospital helpful and useful? *Bmj*. 1989;298(6677):870-4.
76. Perera K.Y., Ranasinghe P., Adikari A.M., Balagobi B., Constantine G.R., Jayasinghe S. Medium of language in discharge summaries: would the use of native language improve patients' knowledge of their illness and medications? *Journal of Health Communication*. 2012;17(2):141-8.
77. Wimsett J., Harper A., Jones P. Review article: Components of a good quality discharge summary: a systematic review. *Emergency Medicine Australasia*. 2014;26(5):430-8.
78. Choudhry A.J., Baghdadi Y.M., Wagie A.E., Habermann E.B., Heller S.F., Jenkins D.H., Cullinane D.C., Zielinski M.D. Readability of discharge summaries: with what level of information are we dismissing our patients? *American Journal of Surgery*. 2016;211(3):631-6.
79. Reddick B., Holland C. Reinforcing discharge education and planning. *Nursing Management*. 2015;46(5):10-4.
80. Brown C.E., Roberts N.J., Partridge M.R. Does the use of a glossary aid patient understanding of the letters sent to their general practitioner? *Clin Med (Lond)*. 2007;7(5):457-60.
81. Fayers T., Abdullah W., Walton V., Wilkins M.R. Impact of written and photographic instruction sheets on patient behavior after cataract surgery. *Journal of Cataract & Refractive Surgery*. 2009;35(10):1739-43.
82. Zeng-Treitler Q., Kim H., Hunter M. Improving patient comprehension and recall of discharge instructions by supplementing free texts with pictographs. *AMIA* 2008;Annual Symposium Proceedings/AMIA Symposium.:849-53.
83. Samuels-Kalow M., Rhodes K., Uspal J., Reyes Smith A., Hardy E., Mollen C. Unmet Needs at the Time of Emergency Department Discharge. *Academic Emergency Medicine*. 2016;23(3):279-87.
84. Thornber M. A simple and effective communication skill. *Bmj*. 2010;337:a2324.
85. Royal College of Physicians. Writing letters to patients – what's the big deal? 2017 https://www.rcplondon.ac.uk/news/writing-letters-patients-what-s-big-deal.
86. Mrduljas Dujic N., Zitnik E., Pavelin L., Bacic D., Boljat M., Vrdoljak D., Pavlicevic I., Dvornik A., Marusic A., Marusic M. Writing letters to patients as an educational tool for medical students. *BMC Medical Education*. 2013;13:114.
87. McKinstry B. Copying patients in is not as simple as it seems. *Bmj*. 2008;337(a2687):p.1369.
88. Lin R., Tofler G., Spinaze M., Dennis C., Clifton-Bligh R., Nojoumian H., Gallagher R. Patient-directed discharge letter (PADDLE)-a simple and brief intervention to improve patient knowledge and understanding at time of hospital discharge. *Heart Lung and Circulation*. 2012;21:S312.
89. McConnell D., Butow P., Tattersall M. Audiotapes and letters to patients: the practice and views of oncologists, surgeons and general practitioners. *British Journal of Cancer*. 1999;79(11-12):1782.
90. Guidelines on regional immediate discharge documentation for patients being discharged from secondary into primary care. *Guidelines and Audit Implementation Network*



For peer review only - http://bmjopen.bmj.com/site/about/guidelines.xhtml

*(GAIN)*. 2011 https://www.rqia.org.uk/RQIA/files/73/734a792f-f9d4-47f0-830f-31f9db51c82a.pdf.

91.Roberts N.J., Partridge M.R. How useful are post consultation letters to patients? *BMC Medicine*. 2006;4:2-.

92.McEniry B., Pillay I. How do patients in a rural setting respond to receiving a copy of their general practitioners letter? *Ir Med J*. 2008;101(3):84-5.

93.Knight A.H., Mayon-White V. Writing to patients--annual review reports. *Diabetic Medicine*. 1991;8(6):591.

94.Adams D.C., Bristol J.B., Poskitt K.R. Surgical discharge summaries: improving the record. *Annals of the Royal College of Surgeons of England*. 1993;75(2):96-9.

95.Bench S.D., Heelas K., White C., Griffiths P. Providing critical care patients with a personalised discharge summary: a questionnaire survey and retrospective analysis exploring feasibility and effectiveness. *Intensive & Critical Care Nursing*. 2014;30(2):69-76.

96.Paravastu S., Lepping P., Billings P. Copying clinic letters to surgical patients. *The Bulletin of the Royal College of Surgeons of England*. 2007;89(8):288-90.

97.Richards T. Copy them in. *Bmj*. 2008;337:a2324.

98.Buurman B.M., Verhaegh K.J., Smeulers M., Vermeulen H., Geerlings S.E., Smorenburg S., de Rooij S.E. Improving handoff communication from hospital to home: the development, implementation and evaluation of a personalized patient discharge letter. *International Journal for Quality in Health Care*. 2016;28(3):384-90.

99.Todhunter S.L., Clamp P.J., Gillett S., Pothier D.D. Readability of out-patient letters copied to patients: can patients understand what is written about them? *J Laryngol Otol*. 2010;124(3):324-7.

100.Partridge M.R., Roberts N.J. Writing to patients. *Clinical Medicine*. 2006;6(3):319.

101.Tattersall R. Writing for and to patients. *Diabetic Medicine*. 1990;7(10):917-9.

102.Rao P., Andrei A., Fried A., Gonzalez D., Shine D. Assessing quality and efficiency of discharge summaries. *American Journal of Medical Quality*. 2005;20(6):337-43.

103.Vaidya G. Copying letters to patients: Are we ready yet? *Hospital Medicine*. 2004;65(8):454-5.

104.Ahmad N., Ellins, J., Krelle H. & Lawrie, M. Person-centred care: from ideas to action. London: The Health Foundation; 2014. Available from https://www.health.org.uk/sites/default/files/PersonCentredCareFromIdeasToAction.pdf

105.RAMSES. Ramses Guidelines. 2013 http://www.ramesesproject.org/.

106.Wong G., Greenhalgh T., Westhorp G., Buckingham J., Pawson R. RAMESES publication standards: realist syntheses. *BMC Medicine*. 2013;11(1):21.

107.Rycroft-Malone J., Burton C., Hall B., McCormack B., Nutley S., Seddon D., Williams L. Improving skills and care standards in the support workforce for older people: a realist review. *BMJ Open*. 2014;4(5).

108.Rycroft-Malone J., McCormack B., Hutchinson A.M., DeCorby K., Bucknall T.K., Kent B., Schultz A., Snelgrove-Clarke E., Stetler C.B., Titler M. Realist synthesis: illustrating the method for implementation research. *Implementation Science*. 2012;7(1):33.

109.Wong G., Greenhalgh T., Pawson R. Internet-based medical education: a realist review of what works, for whom and in what circumstances. *BMC Medical Education*. 2010;10(1):12.

110.McMahon T., Ward P.R. HIV among immigrants living in high-income countries: a realist review of evidence to guide targeted approaches to behavioural HIV prevention. *Systematic Reviews*. 2012;1:56.

111.Wong G. The Internet in Medical Education: A Worked Example of a Realist Review. *Synthesizing Qualitative Research* http://dx.doi.org/10.1002/9781119959847.ch5: John Wiley & Sons, Ltd; 2011. p. 83-112.



For peer review only - http://bmjopen.bmj.com/site/about/guidelines.xhtml

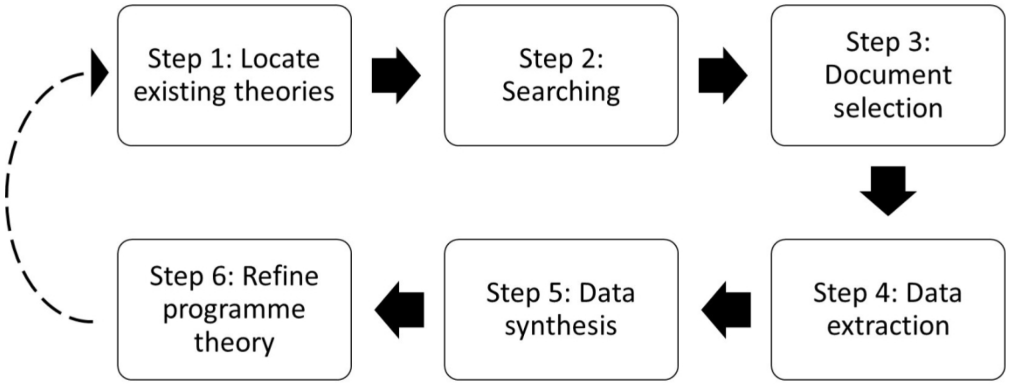
Step 1: Locate existing theories
Step 2: Searching
Step 3: Document selection
Step 4: Data extraction
Step 5: Data synthesis
Step 6: Refine programme theory

Figure 1

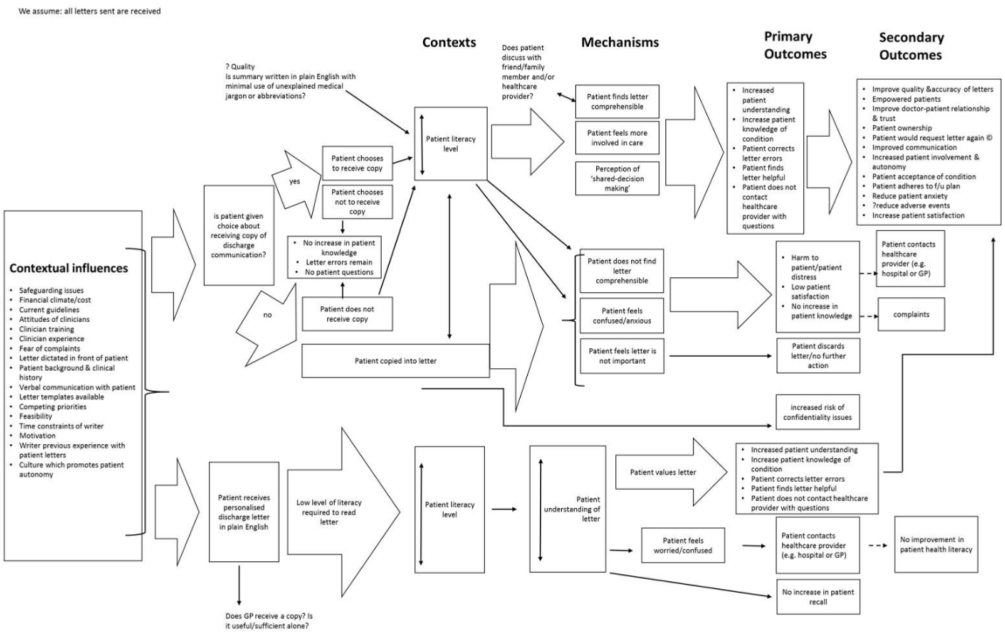
We assume: all letters sent are received
Contexts
Mechanisms
Primary Outcomes
Secondary Outcomes
Contextual influences
• Safeguarding issues
• Financial climate/cost
• Current guidelines
• Attitudes of clinicians
• Clinician training
• Clinician experience
• Fear of complaints
• Letter dictated in front of patient
• Patient background & clinical history
• Verbal communication with patient
• Letter templates available
• Competing priorities
• Feasibility
• Time constraints of writer
• Motivation
• Writer previous experience with patient letters
• Culture which promotes patient autonomy
is patient given choice about receiving copy of discharge communication?
yes
no
Patient chooses to receive copy
Patient chooses not to receive copy
• No increase in patient knowledge
• Letter errors remain
• No patient questions
Patient does not receive copy
Patient copied into letter
? Quality
Is summary written in plain English with minimal use of unexplained medical jargon or abbreviations?
Patient literacy level
Does patient discuss with friend/family member and/or healthcare provider?
Patient finds letter comprehensible
Patient feels more involved in care
Perception of 'shared-decision making'
• Increased patient understanding
• Increase patient knowledge of condition
• Patient corrects letter errors
• Patient finds letter helpful
• Patient does not contact healthcare provider with questions
• Improve quality &accuracy of letters
• Empowered patients
• Improve doctor-patient relationship & trust
• Patient ownership
• Patient would request letter again ©
• Improved communication
• Increased patient involvement & autonomy
• Patient acceptance of condition
• Patient adheres to f/u plan
• Reduce patient anxiety
• ?reduce adverse events
• Increase patient satisfaction
Patient does not find letter comprehensible
Patient feels confused/anxious
Patient feels letter is not important
• Harm to patient/patient distress
• Low patient satisfaction
• No increase in patient knowledge
Patient contacts healthcare provider (e.g. hospital or GP)
complaints
Patient discards letter/no further action
increased risk of confidentiality issues
Patient receives personalised discharge letter in plain English
Low level of literacy required to read letter
Patient literacy level
Patient understanding of letter
Patient values letter
• Increased patient understanding
• Increase patient knowledge of condition
• Patient corrects letter errors
• Patient finds letter helpful
• Patient does not contact healthcare provider with questions
Patient feels worried/confused
Patient contacts healthcare provider (e.g. hospital or GP)
No improvement in patient health literacy
No increase in patient recall
Does GP receive a copy? Is it useful/sufficient alone?

Figure 2

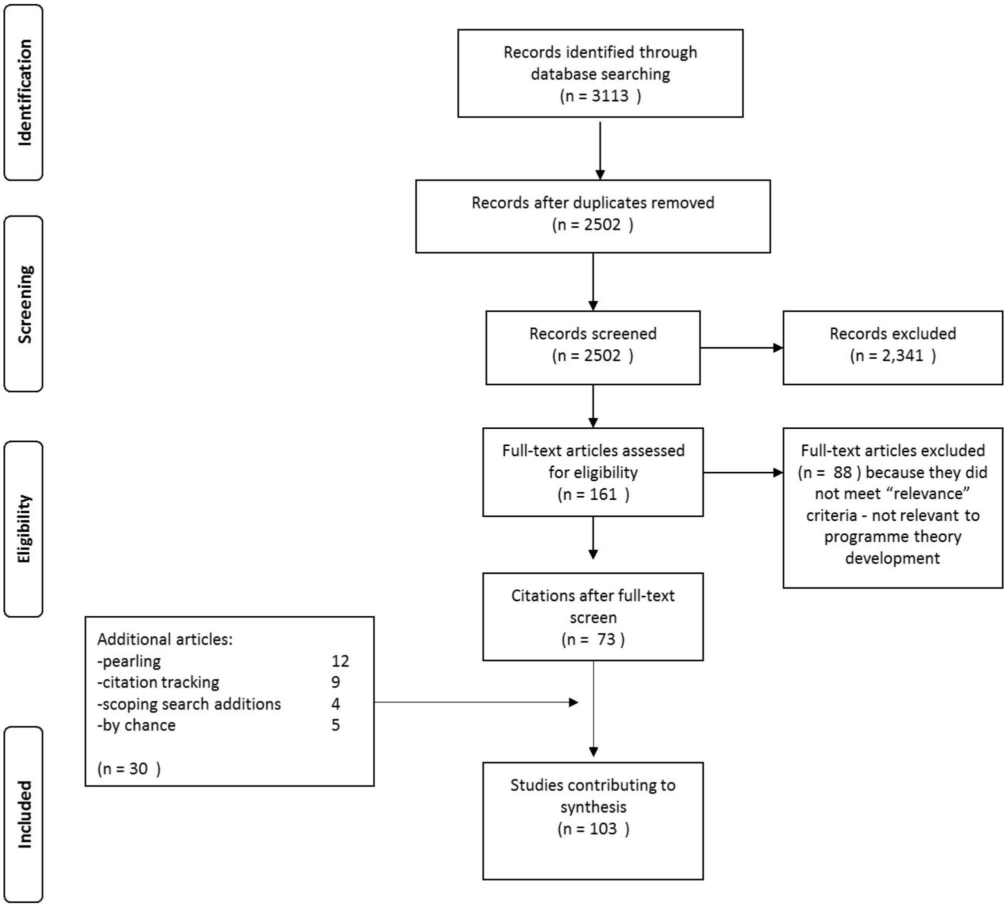
Identification
Screening
Eligibility
Included
Records identified through database searching (n = 3113 )
Records after duplicates removed (n = 2502 )
Records screened (n = 2502 )
Records excluded (n = 2,341 )
Full-text articles assessed for eligibility (n = 161 )
Full-text articles excluded (n = 88 ) because they did not meet "relevance" criteria - not relevant to programme theory development
Citations after full-text screen (n = 73 )
Additional articles:
-pearling 12
-citation tracking 9
-scoping search additions 4
-by chance 5
(n = 30 )
Studies contributing to synthesis (n = 103 )

Figure 3

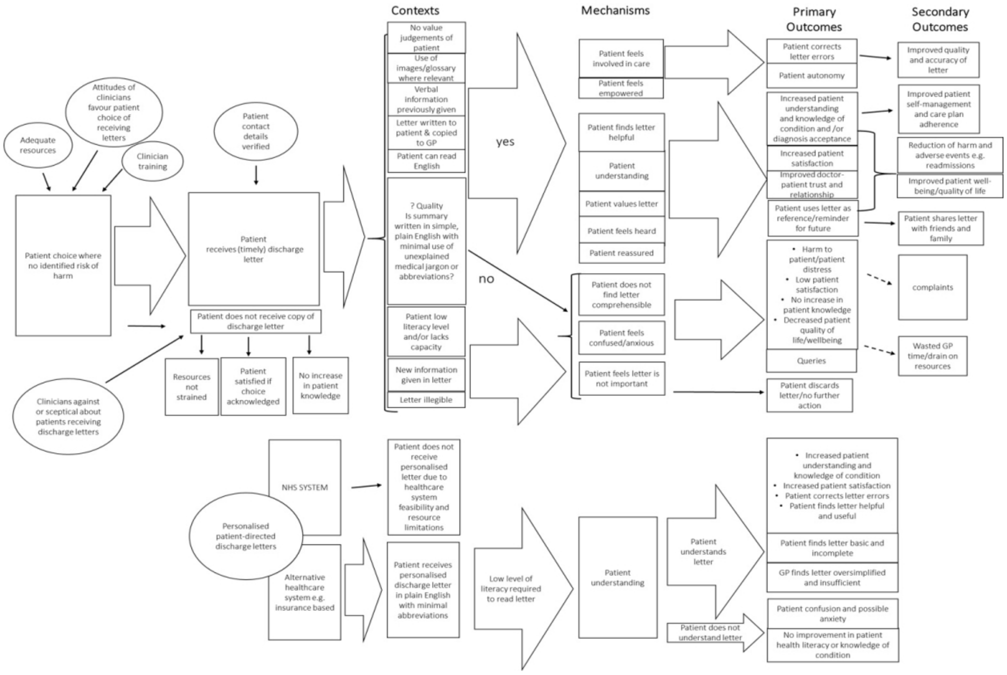
Contexts
Mechanisms
Primary Outcomes
Secondary Outcomes
Adequate resources
Attitudes of clinicians favour patient choice of receiving letters
Clinician training
Patient choice where no identified risk of harm
Patient contact details verified
Patient receives (timely) discharge letter
Patient does not receive copy of discharge letter
Resources not strained
Patient satisfied if choice acknowledged
No increase in patient knowledge
Clinicians against or sceptical about patients receiving discharge letters
No value judgements of patient
Use of images/glossary where relevant
Verbal information previously given
Letter written to patient & copied to GP
Patient can read English
? Quality Is summary written in simple, plain English with minimal use of unexplained medical jargon or abbreviations?
Patient low literacy level and/or lacks capacity
New information given in letter
Letter illegible
yes
no
Patient feels involved in care
Patient feels empowered
Patient finds letter helpful
Patient understanding
Patient values letter
Patient feels heard
Patient reassured
Patient does not find letter comprehensible
Patient feels confused/anxious
Patient feels letter is not important
Patient corrects letter errors
Patient autonomy
Increased patient understanding and knowledge of condition and /or diagnosis acceptance
Increased patient satisfaction
Improved doctor-patient trust and relationship
Patient uses letter as reference/reminder for future
• Harm to patient/patient distress
• Low patient satisfaction
• No increase in patient knowledge
• Decreased patient quality of life/wellbeing
Queries
Patient discards letter/no further action
Improved quality and accuracy of letter
Improved patient self-management and care plan adherence
Reduction of harm and adverse events e.g. readmissions
Improved patient well-being/quality of life
Patient shares letter with friends and family
complaints
Wasted GP time/drain on resources
NHS SYSTEM
Personalised patient-directed discharge letters
Alternative healthcare system e.g. insurance based
Patient does not receive personalised letter due to healthcare system feasibility and resource limitations
Patient receives personalised discharge letter in plain English with minimal abbreviations
Low level of literacy required to read letter
Patient understanding
Patient understands letter
Patient does not understand letter
• Increased patient understanding and knowledge of condition
• Increased patient satisfaction
• Patient corrects letter errors
• Patient finds letter helpful and useful
Patient finds letter basic and incomplete
GP finds letter oversimplified and insufficient
Patient confusion and possible anxiety
No improvement in patient health literacy or knowledge of condition

Figure 4

## Search Terms and Sources Searched

| Source | Search terms |
| --- | --- |
| MEDLINE | 1. written[All Fields] AND ("patient discharge"[MeSH Terms]<br>2. ("patient"[All Fields] AND "discharge"[All Fields])<br>3. ("patient discharge"[All Fields] OR "discharge"[All Fields]) AND ("communication"[MeSH Terms])<br>4. ("communication"[All Fields]) AND ("patient discharge"[MeSH Major Topic]) AND ("patients"[MeSH Terms])<br>5. ("patients"[All Fields] OR "patient"[All Fields]) OR ("letter"[Publication Type] OR "correspondence as topic"[MeSH Terms])<br>6. ("correspondence"[All Fields]) AND ("patients"[MeSH Terms])<br>7. ("patients"[All Fields] OR "patient"[All Fields]) AND "patient discharge"[MeSH Major Topic]) AND ("communication"[MeSH Terms])<br>8. ("communication"[All Fields]) OR (receiving[All Fields]) AND ("letter"[Publication Type]) OR ("correspondence as topic"[MeSH Terms])<br>9. ("letters"[All Fields]) AND ("patients"[MeSH Terms] OR "patients"[All Fields] OR "patient"[All Fields]) AND ("patient discharge"[MeSH Major Topic])<br>10. ("patients"[MeSH Terms] OR "patients"[All Fields] OR "patient"[All Fields]) AND ((copies[All Fields]) AND "patient discharge"[MeSH Major Topic]))<br>11. ((("patient discharge"[MeSH Major Topic] OR "patient discharge"[MeSH Terms]) AND letter[Other Term]) AND ("patients"[MeSH Terms] OR "patients"[All Fields] OR "patient"[All Fields])<br>12. 1 OR 2 OR 3 OR 4 OR 5 OR 6 OR 7 OR 8 OR 9 OR 10 OR 11 |
| Web of Science | 1. Written patient discharge communication<br>2. Patients receiving letters<br>3. Patients receiving discharge letters<br>4. Patient copies of written information<br>5. 1 OR 2 OR 3 OR 4 |
| Department of Health | 1. Discharge communication<br>2. Patient letters |

For peer review only

| | |
|---|---|
| | 3. Patients receiving letters |
| Royal College of Physicians | 1. Discharge communication<br>2. Patient letters<br>3. Patients receiving letters<br>4. Patient copy<br>5. Patient copies<br>6. Patients receiving written information |

For peer review only

**Studies Found from Scoping Search**

| | Author | Year | Title | Document type |
|---|---|---|---|---|
| 1 | D N Wood, A Deshpande, M Wijewardena, and S S Gujral | 2006 | A Study of How Urology Out-Patients like to Receive Clinical Information | Published article |
| 2 | A Liapi, P J Robb, and A Akthar | 2006 | Copying clinic letters to patients: a survey of patient attitudes | Published article |
| 3 | S Baxter, K Farrell, C Brown, J Clarke, and H Davies | 2008 | Where have all the copy letters gone? A review of current practice in professional–patient correspondence | Published article |
| 4 | D D Pothier, P Nakivell, and C E J Hall | 2007 | What do Patients Think about being Copied into their GP Letters? | Published article |
| 5 | S L Todhunter, P J Clamp, S Gillett, and D D Pothier | 2010 | Readability of out-patient letters copied to patients: can patients understand what is written about them? | Published article |
| 6 | Royal College of Physicians | 2013 | Standards for the clinical structure and content of patient records | Guidelines |
| 7 | Royal College of Physicians | 2017 | Writing letters to patients – what's the big deal? | Short website entry |
| 8 | A J Choudhry, Y M K Baghdadi, A E Wagie, E B Habermann, S F Heller, D H Jenkins, D C Cullinane, M D Zielinski | 2016 | Readability of discharge summaries: with what level of information are we dismissing our patients? | Published article |
| 9 | M O'Reilly, M R Cahill, and I J Perry | 2006 | Writing to patients: a randomised controlled trial | Published article |
| 10 | Y Krishna, and B E Damato | 2005 | Patient attitudes to receiving copies of outpatient clinic letters from the ocular oncologist to the referring ophthalmologist | Published article |

For peer review only

| | | | | |
|---|---|---|---|---|
| | | | and GP | |
| 11 | B R O'Driscoll, J Koch, and C Paschalides | 2003 | Most patients want copies of letters from outpatient clinics and find them useful | BMJ letter |
| 12 | H Hadjistavropoulos, H Biem, D Sharpe, M Bourgault-Fagnou, and J Janzen | 2008 | Patient perceptions of hospital discharge: reliability and validity of a Patient Continuity of Care Questionnaire | Published article |
| 13 | M Thornber | 2009 | Copying referral letters | BJGP letter |
| 14 | Department of Health | 2000 | The NHS Plan | Report |
| 15 | P White, A Singleton, and R Jones | 2004 | Copying referral letters to patients: the views of patients, patient representatives and doctors | Published article |
| 16 | NHS England | 2016 | Standards for the communication of patient diagnostic test results on discharge from hospital | Guidance |
| 17 | R Lin, R Gallagher, M Spinaze, H Najoumian, C Dennis, R Clifton-Bligh, and G Tofler | 2014 | Effect of a patient-directed discharge letter on patient understanding of their hospitalisation | Published article |
| 18 | S Vaidyanathan, C A Glass, B M Soni, J Bingley, G Singh, J W H Watt, and P Sett | 2001 | Doctor ± Patient Communication: Do people with spinal cord injury wish to receive written information about their medical condition from the physicians after an outpatient visit or after a readmission in the spinal | Published article |

For peer review only

| | | | | |
|---|---|---|---|---|
| | | | unit? | |
| 19 | J Flacker, W Park, and A Sims, | 2007 | Hospital Discharge Information and Older Patients: Do They Get What They Need? | Published article |
| 20 | J S Albrecht, A L Gruber-Baldini, J M Hirshon, C H Brown, R Goldberg, J H Rosenberg, A C Comer, and J P Furuno, | 2014 | Hospital Discharge Instructions: Comprehension and Compliance Among Older Adults | Published article |
| 21 | B M Buurman, K J Verhaegh, M Smeulers, H Vermeulen, S E Geerlings, S Smorenburg, and S E de Rooij | 2016 | Improving handoff communication from hospital to home: the development, implementation and evaluation of a personalized patient discharge letter | Published article |
| 22 | Department of Health | 2003 | Copying letters to Patients: Good practice guidelines | Guidelines |
| 23 | J Main | 2008 | Copying in or copping out? | BMJ letter |
| 24 | C D Shee | 2008 | Try it and see | BMJ letter |
| 25 | B McKinstry | 2008 | Copying patients in is not as simple as it seems | BMJ letter |
| 26 | D Jelley, and T van Zwanenberg | 2000 | Copying general practitioner referral letters to patients: a study of patients' views | Published article |
| 27 | K Treacy, J S Elborn, J Rendall, and J M Bradley | 2008 | Copying letters to patients with cystic fibrosis (CF): Letter content and patient perceptions of benefit | Published article |

For peer review only

**Sources searched (step 2)**

| | Sources | Results |
|---|---|---|
| 1 | MEDLINE | 1596 |
| 2 | EMBASE | 558 |
| 3 | CINAHL | 100 |
| 4 | DARE | 2 |
| 5 | ASSIA | 47 |
| 6 | Web of Science | 205 |
| 7 | ZETOC | 29 |
| 8 | AMED | 26 |
| 9 | NHS Digital (HSCIC) | 0 |
| 10 | NHS Evidence (public domain only) | 244 |
| 11 | DH | 2 |
| 12 | NICE Guidelines | 0 |
| 13 | Cochrane database of systematic reviews | 21 |
| 14 | EPPI-CENTRE | 20 |
| 15 | SCOPUS | 38 |
| 16 | Google Scholar | 6 |
| 17 | OpenGrey | 3 |
| 18 | Greynet sources | 0 |
| 19 | ProQuest dissertations and theses | 210 |
| 20 | General Medical Council | 0 |
| 21 | Royal College of Physicians | 5 |
| 22 | Local Medical Committees (West Midlands) | 0 |
| 23 | Clinical Commissioning Groups (West Midlands) | 0 |
| 24 | SIGN | 0 |
| 25 | NHS Improvement | 1 |
| | TOTAL RESULTS | 3113 |

For peer review only

**Full List of Search Strategies for each source**

Search strategy for electronic databases (MEDLINE and AMED)

1. letter$
2. summaries$
3. Correspondence
4. patient copies$
5. patient letter
6. communication (MESH term if MEDLINE)
7. patient$ receiving
8. written information
9. discharge document$
10. patient-directed letter
11. personalised letter
12. personal letter
13. personalized letter
14. copy letter
15. 1 OR 2 OR 3 OR 4 OR 5 OR 6 OR 7 OR 8 OR 9 OR 10 OR 11 OR 12 OR 13 OR 14
16. Hospital discharge (MESH if MEDLINE) AND 15
17. Patient discharge (MESH if MEDLINE) AND 15
18. 16 OR 17
19. 18 and patients (MESH HEADING if MEDLINE)
20. Patients adj3 receiving adj3 letter*
21. Patients adj3 receiving adj3 discharge adj letter*
22. patient adj3 cop$ of written adj information
23. written adj3 patient adj discharge adj communication
24. secondary to primary adj care adj3 communication
25. hospital adj3 GP adj3 communication
26. writing adj3 to adj3 patients
27. 20 OR 21 OR 22 OR 23 OR 24 OR 25 OR 26
28. 27 OR 19
29. patient discharge letter
30. discharge communication
31. discharge letter
32. discharge summary
33. discharge summaries
34. 29 OR 30 OR 31 OR 32 OR 33 OR 34 OR 10 OR 11 OR 12 OR 13 OR 14
35. 34 OR 28
36. discharge correspondence
37. copy correspondence
38. doctor letter
39. copy letter
40. 36 OR 37 OR 38 OR 39
41. 40 OR 35

For peer review only

Search strategy for EMBASE and DARE (adapted due to high search results in EMBASE and simpler searching filtration system in DARE):

1. patient directed letter
2. personalised letter
3. personal letter
4. personalized letter
5. copy letter
6. Patients adj3 receiving adj3 letter*
7. Patients adj3 receiving adj3 discharge adj letter*
8. patient adj3 cop$ of written adj information
9. written adj3 patient adj discharge adj communication
10. secondary to primary adj care adj3 communication
11. hospital adj3 GP adj3 communication
12. writing adj3 to adj3 patients
13. patient discharge letter
14. discharge communication
15. discharge letter
16. patient discharge letter
17. discharge correspondence
18. copy correspondence
19. doctor letter
20. copy letter
21. 1 OR 2 OR 3 OR 4 OR 5 OR 6 OR 7 OR 8 OR 9 OR 10 OR 11 OR 12 OR 13 OR 14 OR 15 OR 16 OR 17 OR 18 OR 19 OR 20
22. 21 OR discharge summary (DARE ONLY)

Search strategy for CINAHL (adapted due to high search results):

1. patient discharge letter AND patient discharge from hospital (CINAHL MH "Hand Off (Patient Safety)")
2. personalised letter AND patient discharge from hospital (CINAHL MH "Hand Off (Patient Safety)")
3. copy letter AND patient discharge from hospital (CINAHL MH patient discharge summaries)
4. discharge communication AND patient discharge from hospital (CINAHL MH patient discharge summaries)
5. discharge correspondence AND patient discharge from hospital (CINAHL MH patient discharge summaries)
6. copy correspondence AND patient discharge from hospital (CINAHL MH patient discharge summaries)
7. doctor letter AND patient discharge from hospital (CINAHL MH patient discharge summaries)
8. copy letter
9. 1 OR 2 OR 3 OR 4 OR 5 OR 6 OR 7 OR 8

ASSIA and Web of Science and ZETOC and NHS evidence search strategy and NHS improvement and Cochrane database

1. (patient discharge letter) AND (discharge from hospital) AND "patient discharge" (*for NHS EVIDENCE & improvement & SCOPUS- AND written)

For peer review only

2. (personalised letter) AND (discharge from hospital) AND "patient discharge" (*for NHS EVIDENCE & improvement & SCOPUS - AND written)
3. (copy letter) AND (discharge from hospital) AND "patient discharge" (*for NHS EVIDENCE & improvement - AND written)
4. (doctor letter) AND (discharge from hospital) AND "patient discharge" (*for NHS EVIDENCE & improvement - AND written)
5. 1 OR 2 OR 3 OR 4
6. (FOR NHS EVIDENCE & improvement ONLY) copying hospital discharge letters to patients

ProQuest very high results (adapted terms)

1. personalised patient discharge letter AND "patient discharge" AND written AND patient information AND copy AND personalised AND copy letter AND doctor letter AND discharge communication copy OR letter "discharge from hospital"Google Scholar search: (many thousands of results when using above terms)

1. (personalised patient discharge letter) AND (discharge from hospital) AND "patient discharge" AND written AND patient information AND copy AND personalised AND copy letter AND doctor letter AND discharge communication AND written AND patient copy

Broad searches for X, Y, Z (websites and sources without indexing or electronic searching) e.g. Department of health

Used for: HSCIC and EPPI-CENTRE And Open Grey

The below search terms were entered into GMC yielding several hundred results. As results from GMC must be exported singularly these were screened on the webpage. The searches found no relevant results.

1. Discharge communication
2. Discharge
3. Patient discharge
4. Discharge letter
5. Discharge letters
6. Discharge summary
7. Discharge summaries
8. Patient letters
9. Patients receiving letters
10. Patients receiving letter
11. Patient copy
12. Copying patients
13. Patient copies
14. Patients receiving written information
15. Hospital discharge
16. Discharge correspondence
17. Discharge document
18. Discharge information
19. Patient discharge information

For peer review only

20. Copy letter
21. Doctor letter
22. Personal letter
23. Copy correspondence
24. Patient involvement
25. Patient access to records
26. Health informatics

*searches adapted in NHS digital due to huge amount of irrelevant results around admission statistics. No relevant results could be found.

For peer review only

## List of Included Texts (full)


1. Ackermann S, Bingisser MB, Heierle A, Langewitz W, Hertwig R, Bingisser R. Discharge communication in the emergency department: physicians underestimate the time needed. Swiss Med Wkly. 2012;142:w13588.
2. Adams DC, Bristol JB, Poskitt KR. Surgical discharge summaries: improving the record. Ann R Coll Surg Engl. 1993;75(2):96-9.
3. Advancing effective communication, cultural competence, and patient- and family-centered care : a roadmap for hospitals. Joint Commission. 2014 http://www.jointcommission.org/assets/1/6/aroadmapforhospitalsfinalversion727.pdf.
4. Aguayo-Albasini JL, Garcia Garcia ML, Flores-Pastor B, Liron-Ruiz R. The importance of the discharge summary reports. Cir Esp. 2014;92(8):574-5.
5. Allan K, Ribbons B. Nurses combine IT and nursing skills to improve discharge communication. Aust Nurs J. 2006;14(1):30.
6. Antoniou A, Saunders M, Bourner R, Crouch L. would you like to see yours? The Bulletin of the Royal College of Surgeons of England. 2007;89(2):62-4.
7. Baumann W, Schussler L, Bertram M, Benser J, Kumpers S, Hermes-Moll K. Oncologists' letters for breast cancer patients. Oncology Research and Treatment. 2016;39:184-5.
8. Baxter S, Farrell K, Brown C, Clarke J, Davies H. Where have all the copy letters gone? A review of current practice in professional-patient correspondence. Patient Educ Couns. 2008;71(2):259-64.
9. Bench S, Day T, Griffiths P. Effectiveness of critical care discharge information in supporting early recovery from critical illness. Crit Care Nurse. 2013;33(3):41-52.
10. Bench SD, Heelas K, White C, Griffiths P. Providing critical care patients with a personalised discharge summary: a questionnaire survey and retrospective analysis exploring feasibility and effectiveness. Intensive Crit Care Nurs. 2014;30(2):69-76.
11. Bench S, Day T, Heelas K, Hopkins P, White C, Griffiths P. Evaluating the feasibility and effectiveness of a critical care discharge information pack for patients and their families: a pilot cluster randomised controlled trial. BMJ Open. 2015;5(11):e006852.
12. Boaden R, Harris C. Copying letters to patients—will it happen? : Oxford University Press; 2005.
13. Brockbank K. Copying patient letters - Making it work. Clinical Governance. 2005;10(3):231-40.
14. Brodie T, Lewis D. A survey of patient views on receiving vascular outpatient letters. European Journal of Vascular and Endovascular Surgery. 2010;39(1):5-10.
15. Brown CE, Roberts NJ, Partridge MR. Does the use of a glossary aid patient understanding of the letters sent to their general practitioner? Clinical medicine (London, England). 2007;7(5):457-60.
16. Buurman BM, Verhaegh KJ, Smeulers M, Vermeulen H, Geerlings SE, Smorenburg S, et al. Improving handoff communication from hospital to home: the development, implementation and evaluation of a personalized patient discharge letter. International Journal for Quality in Health Care. 2016;28(3):384-90.
17. Cannaby A-M. Improving the process of hospital discharge for medical patients [Ph.D.]. Ann Arbor: University of Leicester (United Kingdom); 2003.
18. Carol Lim KK, Chan SK, Chew EL, Anita Lim AF, Sararaks S, Ainul H, et al. Handoff communication - Let's do it right. Medical Journal of Malaysia. 2010;65:8.
19. Chantler C, Johnson J. Patients should receive copies of letters and summaries. BMJ : British Medical Journal. 2002;325(7360):388-.


For peer review only

20. Charlett SD, Bajaj Y, Kelly G. Writing to patients with the results of routine tests: A measure to improve access to outpatient clinics. Otorhinolaryngologist. 2009;2(3):73-4.
21. Choudhry AJ, Baghdadi YM, Wagie AE, Habermann EB, Heller SF, Jenkins DH, et al. Readability of discharge summaries: with what level of information are we dismissing our patients? Am J Surg. 2016;211(3):631-6
22. Damian D, Tattersall MH. Letters to patients: improving communication in cancer care. Lancet. 1991;338(8772):923-5.
23. Davies JM, Batuyong E, Lupichuk SM, Hilsden R, Eliasziw M, Easaw JC. Cohort study evaluating the impact of a discharge letter (DL) compared with usual care on adherence to surveillance following treatment for stage II/III colorectal cancer (CRC). Journal of Clinical Oncology Conference. 2012;30(4 SUPPL. 1).
24. Department of Health. The NHS Plan. 2000 http://webarchive.nationalarchives.gov.uk.
25. Department of Health. Copying letters to patients: good practice guidelines. 2003 http://webarchive.nationalarchives.gov.uk/
26. Discharge planning : best practice in transitions of care. The Queen's Nursing Institute. 2016 https://www.qni.org.uk/wpcontent/uploads/2016/09/discharge_planning_report_2015.pdf.
27. Dooher P, Syed A, Liu J, Chopra A, Bradpiece H, Jenkins S, et al. Copying letter to patients- distress or satisfaction? Eur J Cancer. 2012;48:S151.
28. Exploring patient participation in reducing health-care-related safety risks. 2013 http://www.euro.who.int/__data/assets/pdf_file/0010/185779/e96814.pdf.
29. Fayers T, Abdullah W, Walton V, Wilkins MR. Impact of written and photographic instruction sheets on patient behavior after cataract surgery. J Cataract Refract Surg. 2009;35(10):1739-43.
30. Fenton C, Al-Ani A, Trinh A, Srinivasan A, Marion K, Hebbard G. Impact of providing patients with copies of their medical correspondence: a randomised controlled study. Intern Med J. 2017;47(1):68-75.
31. Generic Standards Mar 2002. 2002 http://www.healthcareimprovementscotland.org/his/idoc.ashx?docid=ea8d290a-9504-4c21-a889-fc479d530a51&version=-1.
**32.** Guidelines on regional immediate discharge documentation for patients being discharged from secondary into primary care. Guidelines and Audit Implementation Network (GAIN). 2011 https://www.rqia.org.uk/RQIA/files/73/734a792f-f9d4-47f0-830f-31f9db51c82a.pdf**.**
33. Hahn-Goldberg S, Okrainec K, Damba C, Huynh T, Lau D, Maxwell J, et al. Implementing Patient-Oriented Discharge Summaries (PODS): A Multisite Pilot Across Early Adopter Hospitals. Healthc Q. 2016;19(1):42-8.
34. Hallowell N. Providing letters to patients. Patients find summary letters useful. Bmj. 1998;316(7147):1830.
35. Hayes KS. Literacy for health information of adult patients and caregivers in a rural emergency department. Clin Excell Nurse Pract. 2000;4(1):35-40.
36. HPOE: A Compendium of Implementation Guides 2011. 2011 http://www.hpoe.org/Reports-HPOE/hpoe_compendium_2011.pdf.
37. Hoek AE, De Ridder MA, Bayliss A, Patka P, Rood PP. Effective strategy for improving instructions for analgesic use in the emergency department. Eur J Emerg Med. 2013;20(3):210-3.
38. Holm H, Viktil KK. Patient satisfaction with drug reconciliation at discharge from hospital. Int J Clin Pharm. 2013;35 (6):1284.

For peer review only

39. Jelley D, van Zwanenberg T, Walker C. Copying letters to patients : Concerns of clinicians and patients need to be addressed first. BMJ : British Medical Journal. 2002;325(7376):1359-.
40. Knight AH, Mayon-White V. Writing to patients--annual review reports. Diabet Med. 1991;8(6):591.
41. Krishna Y, Damato BE. Patient attitudes to receiving copies of outpatient clinic letters from the ocular oncologist to the referring ophthalmologist and GP. Eye (London, England). 2005;19(11):1200-4.
42. Lepping P, Paravastu SC, Turner J, Billings P, Minchom P. Copying GP letters to patients: a comprehensive study across four different departments in a district general hospital. Health Inform J. 2010;16(1):58-62.
43. Liapi A, Robb PJ, Akthar A. Copying clinic letters to patients: a survey of patient attitudes. The Journal of Laryngology & Otology. 2006;121(6):588-91.
44. Lim CKK, Lim AAF, Ainul Nadziha MH, Roslinah A, Sararaks S, Chan SK, et al. Boleh balik! Medical Journal of Malaysia. 2010;65:69.
45. Lin R, Tofler G, Spinaze M, Dennis C, Clifton-Bligh R, Nojoumian H, et al. Patient-directed discharge letter (PADDLE)-a simple and brief intervention to improve patient knowledge and understanding at time of hospital discharge. Heart Lung and Circulation. 2012;21:S312.
46. Lin R, Gallagher R, Spinaze M, Najoumian H, Dennis C, Clifton-Bligh R, et al. Effect of a patient-directed discharge letter on patient understanding of their hospitalisation. Internal Medicine Journal. 2014;44(9):851-7.
47. Lin MJ, Tirosh AG, Landry A. Examining patient comprehension of emergency department discharge instructions: Who says they understand when they do not? Intern. 2015;10(8):993-1002.
48. Lorenzati B, Quaranta C, Perotto M, Tartaglino B, Lauria G. Discharge communication is an important underestimated problem in emergency department. Intern. 2016;11(1):157-8
49. Main J. Copying in or copping out? Bmj. 2008;337:a2688.
50. Making time in general practice : freeing GP capacity by reducing bureaucracy and avoidable consultations, managing the interface with hospitals and exploring new ways of working. NHS Alliance. 2015 http://www.nhsalliance.org/wp-content/uploads/2015/10/Making-Time-in-General-Practice-FULL-REPORT-01-10-15.pdf.
51. McConnell D, Butow P, Tattersall M. Audiotapes and letters to patients: the practice and views of oncologists, surgeons and general practitioners. British Journal of Cancer. 1999;79(11-12):1782.
52. McEniry B, Pillay I. How do patients in a rural setting respond to receiving a copy of their general practitioners letter? Ir Med J. 2008;101(3):84-5.
53. McKinstry B. Copying patients in is not as simple as it seems. Bmj. 2008;337:a2687.
54. Menon GJ, Dutton GN. Writing to our patients. Br J Ophthalmol. 1999;83(7):765.
55. Mrduljas Dujic N, Zitnik E, Pavelin L, Bacic D, Boljat M, Vrdoljak D, et al. Writing letters to patients as an educational tool for medical students. BMC Med Educ. 2013;13:114.
56. Mrduljas-Djujic N, Pavlicevic I, Marusic A, Marusic M. Students letters to patients as a part of education in family medicine. Acta Med. 2012;41(1):52-8.
57. NHS England . Standards for the communication of patient diagnostic test results on discharge from hospital. 2016. https://improvement.nhs.uk/uploads/documents/discharge-standards-march-16.pdf.
58. Nixon J, Courtney P. Copying clinic letters to patients. Rheumatology (Oxford). 2005;44(2):255-6.


For peer review only

59. O'Driscoll BR, Koch J, Paschalides C. Copying letters to patients: Most patients want copies of letters from outpatient clinics and find them useful. BMJ : British Medical Journal. 2003;327(7412):451-.
60. O'Reilly M, Cahill MR, Perry IJ. Writing to patients: a randomised controlled trial. Clin Med. 2006;6(2):178-82.
61. Paravastu S, Lepping P, Billings P. Copying clinic letters to surgical patients. The Bulletin of the Royal College of Surgeons of England. 2007;89(8):288-90.
62. Partridge MR, Roberts NJ. Writing to patients. Clin Med. 2006;6(3):319.
63. Perera KY, Ranasinghe P, Adikari AM, Balagobi B, Constantine GR, Jayasinghe S. Medium of language in discharge summaries: would the use of native language improve patients' knowledge of their illness and medications? J Health Commun. 2012;17(2):141-8
64. Perkins P, Jordan A, Prentice W, Regnard C. Copying letters to patients: a survey of patients and GPs views. Palliat Med. 2007;21(4):355-6.
65. Physicians RCo. Standards for the clinical structure and content of patient records. 2013 https://www.rcplondon.ac.uk/projects/outputs/standards-clinical-structure-and-content-patient-records.
66. Physicians RCo. Writing letters to patients – what's the big deal? 2017 https://www.rcplondon.ac.uk/news/writing-letters-patients-what-s-big-deal.
67. Pierce L. How to choose and develop written educational materials. Rehabilitation Nursing. 2010;35(3):99-105.
68. Pinder E, Jefferys S, Loeffler M. Patient Satisfaction: Receiving a copy of the GP letter following fracture or elective orthopaedic clinic. BMJ Quality Improvement Reports. 2013;2(2).
69. Polster D. Patient discharge information. Nursing. 2015;45(5):42-9.
70. Pothier DD, Nakivell P, Hall CE. What do patients think about being copied into their GP letters? Ann R Coll Surg Engl. 2007;89(7):718-21.
71. PRSB. Outpatient letter standard Consultation survey. 2017.
72. Rao M, Fogarty P. What did the doctor say? J Obstet Gynaecol. 2007;27(5):479-80.
73. Reddick B, Holland C. Reinforcing discharge education and planning. Nurs Manage. 2015;46(5):10-4.
74. Regalbuto R, Maurer MS, Chapel D, Mendez J, Shaffer JA. Joint Commission requirements for discharge instructions in patients with heart failure: is understanding important for preventing readmissions? J Card Fail. 2014;20(9):641-9.
75. Reilly MM. Let's set the record straight: preparing the discharge summary and the patient's instruction sheet. Nursing. 1979;9(1):56-61.
76. Reilly MO, Cahill M, Perry IJ. Writing to patients: 'putting the patient in the picture'. Ir Med J. 2005;98(2):58-60.
77. Roberts NJ, Partridge MR. How useful are post consultation letters to patients? BMC Medicine. 2006;4:2-.
78. Saidinejad M, Zorc J. Mobile and web-based education: delivering emergency department discharge and aftercare instructions. Pediatr Emerg Care. 2014;30(3):211-6.
79. Samuels-Kalow M, Rhodes K, Uspal J, Reyes Smith A, Hardy E, Mollen C. Unmet Needs at the Time of Emergency Department Discharge. Acad Emerg Med. 2016;23(3):279-87
80. Sandler DA, Heaton C, Garner ST, Mitchell JR. Patients' and general practitioners' satisfaction with information given on discharge from hospital: audit of a new information card. Bmj. 1989;299(6714):1511-3.
81. Sandler DA, Mitchell JR, Fellows A, Garner ST. Is an information booklet for patients leaving hospital helpful and useful? Bmj. 1989;298(6677):870-4.

For peer review only

82. Saunders NC, Georgalas C, Blaney SP, Dixon H, Topham JH. Does receiving a copy of correspondence improve patients' satisfaction with their out-patient consultation? J Laryngol Otol. 2003;117(2):126-9.
83. Sharma D, O'Brien S, Hardy K. Copying letters to patients: What patients think - A questionnaire survey. Clinician in Management. 2007;15(2):75-8.
84. Shee CD. Try it and see. Bmj. 2008;337:a2786.
85. Singh S, Budeda B, Housden P. Do patients want copies of their GP letters?–our experience with 7250 patients. Int J Clin Pract. 2007;61(8):1407-9.
86. Smith PEM. Letters to patients: sending the right message. BMJ : British Medical Journal. 2002;324(7338):685-.
87. Somov P, Madden T, Wong K, Hamm R. Security Concerns About Copying Clinical Letters to Patients. The Bulletin of the Royal College of Surgeons of England. 2013;95(1):33-4.
88. Sparkler 1 - Transitions of care in elderly patients. 2014 http://emahsn.org.uk/images/Section%208%20-%20Resource%20hub/Sparks%20and%20Sparklers/Sparkler_1_v6_SP1V1_FINAL_pdf_01-08-14.pdf.
89. Synthesis and conceptual analysis of the SDO programme's research on continuity of care. National Institute for Health Research (NIHR). 2010 http://www.netscc.ac.uk/hsdr/files/project/SDO_FR_08-1813-248_V01.pdf.
90. Tattersall R. Writing for and to patients. Diabet Med. 1990;7(10):917-9.
91. Taylor DM, Cameron PA. Discharge instructions for emergency department patients: What should we provide? Journal of Accident and Emergency Medicine. 2000;17(2):86-90.
92. Thornber M. Copy them in. Bmj. 2008;337.
93. Todhunter SL, Clamp PJ, Gillett S, Pothier DD. Readability of out-patient letters copied to patients: can patients understand what is written about them? J Laryngol Otol. 2010;124(3):324-7.
94. Tomkins CS, Braid JJ, Williams HC. Do dermatology outpatients value a copy of the letter sent to their general practitioner? In what way and at what cost? Clin Exp Dermatol. 2004;29(1):81-6.
95. Treacy K, Elborn JS, Rendall J, Bradley JM. Copying letters to patients with cystic fibrosis (CF): letter content and patient perceptions of benefit. Journal of cystic fibrosis : official journal of the European Cystic Fibrosis Society. 2008;7(6):511-4.
96. The Newcastle upon Tyne Hospitals NHS Foundation Trust Sharing Letters with Patients Policy. 2013.
97. Vaidya G. Copying letters to patients: Are we ready yet? Hospital Medicine. 2004;65(8):454-5.
98. Verhaegh KJ, Buurman BM, Veenboer GC, de Rooij SE, Geerlings SE. The implementation of a comprehensive discharge bundle to improve the discharge process: a quasi-experimental study. Neth J Med. 2014;72(6):318-25.
99. Walji M, Loeffelholz J, Valenza JA. A human-centered design of a dental discharge summary (DDS) for patients. AMIA Annu Symp Proc. 2007;Annual Symposium Proceedings/AMIA Symposium.:1146.
100. Warren J, Adnan M, Orr M. Iterative refinement of SemLink to enhance patient readability of discharge summaries. Stud Health Technol Inform. 2013;188:128-34.
101. Wimsett J, Harper A, Jones P. Review article: Components of a good quality discharge summary: a systematic review. Emerg Med Australas. 2014;26(5):430-8.


For peer review only

102. Zavala S, Shaffer C. Do patients understand discharge instructions? J Emerg Nurs. 2011;37(2):138-40.

103. Zeng-Treitler Q, Kim H, Hunter M. Improving patient comprehension and recall of discharge instructions by supplementing free texts with pictographs. AMIA Annu Symp Proc. 2008;Annual Symposium Proceedings/AMIA Symposium.:849-53.

For peer review only

## CMO Table

| | Context | Mechanism | Outcome | Effect assessment | Does it "work" or not? |
|---|---|---|---|---|---|
| CMOC1 | patient not offered letter | patient feels less involved in care | reduced patient autonomy | negative | does not work |
| CMOC2 | patient offered opportunity to receive letter(s)/patient choice respected | patient feels more informed and involved in care | increased patient autonomy and increased involvement of patients in treatment, care and communications | positive | does work |
| CMOC3 | large clear posters displaying patients right to choose and importance of correct contact information | patient realises they should inform hospital of address changes and preferences | lowered risk of confidentiality breach | positive | does work |
| CMOC4 | NHS drive for patient-led care (influence or context) | clinicians increasingly offering patient choice of receiving letter/sharing information with patients | increased patient empowerment | positive | does work |
| CMOC5 | clinician views letters to patients are beneficial e.g. increases transparency, compliance, trust, patient satisfaction, patient understanding and recall | clinician feels patient should be offered letter | potential increase in patient autonomy & satisfaction | positive | does work |
| CMOC6 | Clinicians views letters to patients as not beneficial e.g. letter not comprehensible to patient, medico-legal issues, increased cost and staff workload, patient harm (anxiety, distress, and confusion) and issues around confidentiality | clinician feels patient should not be offered letter | no patient autonomy | N/A | unclear |
| CMOC7 | NHS guidance that all hospital-GP correspondence should be copied to patient as a "right" where appropriate and if patients agree (unless risk of serious harm or legal issues) | clinicians increasingly offering patient choice of receiving letter | increased use of NHS resources to send letters but patient benefits through increased understanding & potential reduction in patient queries (costs balanced) | positive | does work |
| CMOC8 | Data Protection Act 1998 (UK) | Patients may become aware of their right to know what is written & stored about them | Patients informed of their stored electronic information (increased transparency) | positive | does work |
| CMOC9 | doctors copy patients letters | patient trusts doctor more | improved doctor-patient relationship | positive | does work |
| CMOC10 | patients offered choice of receiving letters | increased no. of patients choosing to receive letters | Increased administrative staff workload and costs of printing & posting letters | negative | unclear |

For peer review only

| | Context | Mechanism | Outcome | Effect assessment | Does it "work" or not? |
|---|---|---|---|---|---|
| CMOC11 | patients offered choice of receiving letters | increased no. of patients choosing to receive letters | reduced queries and GP visits and reduced hospital re-admissions (limited evidence) | positive | does work |
| CMOC12 | structured discharge letters written clearly in plain English (pref. 5th grade level) with medical jargon defined, no value judgements of patients and minimal abbreviations | patients understand letter | increased patient knowledge | positive | does work |
| CMOC13 | doctors provided training in letter writing & record keeping (contextual influence) leading to doctors write letters of higher quality and more appropriate for patients | patients understand letter | Increased patient knowledge/potential increase in doctor confidence in letter writing | positive | does work |
| CMOC14 | patients preference for letter copies acknowledged and patients offered choice of receiving letter | patients feel able to express their preference | decreased strain on resources & increased patient autonomy & satisfaction | positive | does work |
| CMOC15 | patients provided written & verbal information | patients reflect on written record of information for reference | increased patient knowledge of care plan, recall and acceptance of illness or condition | positive | does work |
| CMOC16 | Human Rights Act (1998) and Race Revelations Act (2000) - clinicians equally offer all patients letter copies regardless of background | clinician feels all patients should be offered letter | increased equality and accessibility of information to patients | positive | does work |
| CMOC17 | Use of pictures/pictographs/cartoons with written information | patients understand letter | Patient benefits from improved understanding e.g. adherence to agreed care plan | positive | does work |
| CMOC18 | verbal information only | patient may not be able to retain information | reduced patient recall | negative | does not work |
| CMOC19 | professionals who are not involved/limited involvement with patient writes letter | professional does not understand patient plan | letter quality reduced/increased risk of harm | negative | does not work |
| CMOC20 | patient hospital visit of sensitive nature and/or patient lacks capacity e.g. psychotic episode, dementia | patient finds letter distressing and/or confusing | harm to patient | negative | does not work |
| CMOC21 | Patient letter written above patient educational level or in a language the patient does not read | patient finds letter difficult to understand | patient is confused with no increased knowledge of care/possible misinterpretation of care instructions | negative | does not work |
| CMOC22 | letter contains inaccurate information | patient identifies inaccuracies | patient notifies hospital/GP of inaccuracies and corrections are made leading to improved record keeping | positive | does work |

For peer review only

| | Context | Mechanism | Outcome | Effect assessment | Does it "work" or not? |
|---|---|---|---|---|---|
| CMOC23 | patients receives discharge letter | patient does not understand entirety of letter | patient sources answers (internet, GP, friend or relative) | positive | does work |
| CMOC24 | Patient specific letter sent to patient | patient finds letter clear | improved patient comprehension/patient may use letter as aid to explain condition to family and friends | positive | does work |
| CMOC25 | Patient specific letter sent to patient | | increased staff workload and costs | negative | does not work |
| CMOC26 | Patient specific letter sent to patient | Patient identifies information sent to GP and patient is different | medico-legal concerns could be raised over letter discrepancies and any withheld information | negative | does not work |
| CMOC27 | hospital sends patient discharge letter without verifying patient contact details without notifying patient | hospital worker does not identify and correct incorrect information | potential breach of patient confidentiality | negative | does not work |
| CMOC28 | hospital routinely checks patient addresses and sends discharge letters to patients marked confidential using full name | hospital worker identifies and corrects incorrect information | patient receives letter, minimal risk of patient confidentiality breach | positive | does work |
| CMOC29 | patient receives discharge letter | patient may feel they have questions relating to letter | patient contacts health provider with queries (evidence suggests minimal impact and queries) | positive | unclear |
| CMOC30 | discharge letter/summary dictated in front of patient | patients query any inaccuracies | letter less likely to contain inaccuracies | positive | does work |
| CMOC31 | Hospital gives patient discharge letter/summary to deliver to GP | patient may find they are unable to make delivery | GP does not always receive letter/summary | negative | does not work |
| CMOC32 | Patient receives letter not written at appropriate level for them | patient feels confused and does not understand letter | GP spends time reassuring patient and explaining letter to ease patient upset | negative | does not work |
| CMOC33 | Patients have anxiety that doctors talk about things behind their backs | patients who receive letter feel reassured that there is no hidden information | decreased patient anxiety and improved doctor-patient relationship through transparency | positive | does work |
| CMOC34 | patients receives discharge letter | Patients feel they are important to clinician | patient is impressed with letter and feels clinician has an interest in them | positive | does work |
| CMOC35 | choice about whether letter is sent to patient | clinician feels letters would be a disaster and inappropriate for patients | patients do not receive letters | N/A | unclear |
| CMOC36 | patients receives discharge letter | | no impact on patient | N/A | unclear |
| CMOC37 | patients receives discharge letter with bad news | Patient finds letter initially distressing | letter causes initial distress but final outcome that patient finds letter helpful and aids recall and acceptance of condition | positive | does work |

For peer review only

| | Context | Mechanism | Outcome | Effect assessment | Does it "work" or not? |
|---|---|---|---|---|---|
| CMOC38 | letter sent to patient containing information not discussed with patient or abnormal results | patient feels distressed and anxious reading letter | patient harm/unethical practice | negative | does not work |
| CMOC39 | patient worried about diagnosis and receives letter | patient understanding helped by letter | patient feels less anxious due to being more informed | positive | does work |
| CMOC40 | patients preference for letter copies not acknowledged | | patient may receive letter who didn't want one leading to decreased patient satisfaction | negative | does not work |
| CMOC41 | (best practice) patients offered choice of receiving letters/opt-in system | patients enabled to decide on letter preference | patients may or may not receive letter depending on their preference resulting in higher patient satisfaction | positive | does work |
| CMOC42 | patients who feel copies of letters are not necessary for themselves | | patient not given letter so patient satisfied, secondary outcomes: costs and time saved | positive | does work |
| CMOC43 | patients receives discharge letter where appropriate | patient understands letter (high evidence) | patient finds letter informative and helpful | positive | does work |
| CMOC44 | patients receives discharge letter where appropriate | patient feels involved in care plan | patient ensures follow up plan is followed and books any necessary tests etc. | positive | does work |
| CMOC45 | patients receives discharge letter where appropriate | patient feels letter is important | letter forms permanent record of hospital visit and kept for future reference | positive | does work |
| CMOC46 | patients receives discharge letter for breaking good news | patient reminded of discussion | patient feels reassured and has "peace of mind" | positive | does work |
| CMOC47 | patients receives discharge letter where appropriate (patient choice) | patient likes receiving letter | patient satisfaction increased | positive | does work |
| CMOC48 | patients receives copy of discharge letter where appropriate | patient becomes aware of what GP knows | Patient reassured that GP knows about visit | positive | does work |

For peer review only
